# The impact of past HIV interventions and diagnosis gaps on new HIV acquisitions, transmissions, and HIV-related deaths in Côte d’Ivoire, Mali, and Senegal

**DOI:** 10.1097/QAD.0000000000003974

**Published:** 2024-07-01

**Authors:** Romain Silhol, Mathieu Maheu-Giroux, Nirali Soni, Arlette Simo Fotso, Nicolas Rouveau, Anthony Vautier, Clémence Doumenc-Aïdara, Olivier Geoffroy, Kouassi Noël N’Guessan, Younoussa Sidibé, Odé Kanku Kabemba, Papa Alioune Gueye, Pauline Dama Ndeye, Christinah Mukandavire, Peter Vickerman, Abdelaye Keita, Cheikh Tidiane Ndour, Eboi Ehui, Joseph Larmarange, Marie-Claude Boily

**Affiliations:** a MRC Centre for Global Infectious Disease Analysis, School of Public Health, Imperial College London, London, UK; b Department of Epidemiology and Biostatistics, School of Population and Global Health, Faculty of Medicine and Health Sciences, McGill University, Montréal, QC, Canada; c Centre Population & Développement, Université Paris Cité, IRD, Inserm, Paris; d Institut National d’Études Démographiques, INED, Aubervilliers; e Solidarité Thérapeutique et Initiatives pour la Santé, Solthis, Paris, France; f Solidarité Thérapeutique et Initiatives pour la Santé, Solthis, Dakar, Sénégal; g Solidarité Thérapeutique et Initiatives pour la Santé, Solthis, Abidjan, Côte d’Ivoire; h Solidarité Thérapeutique et Initiatives pour la Santé, Solthis, Bamako, Mali; i Department of Epidemiology and Data Science, Coalition for Epidemic Preparedness and Innovations, London, UK; j School of Mathematics and Data Science, Emirates Aviation University, Dubai, United Arab Emirates; k Population Health Sciences, Bristol Medical School, University of Bristol, Bristol, UK; l Institut National de Santé Publique (INSP), Bamako, Mali; m Division de Lutte contre le Sida et les IST, Ministère de la Santé et de l’Action Sociale Institut d’Hygiène Sociale, Dakar, Sénégal; n Programme National de Lutte contre le Sida, Abidjan, Côte d’Ivoire.

**Keywords:** female sex workers, HIV diagnosis, HIV epidemiology, HIV incidence, key and vulnerable populations, men who have sex with men, Western Africa

## Abstract

**Objectives:**

To estimate the epidemiological impact of past HIV interventions and the magnitude and contribution of undiagnosed HIV among different risk groups on new HIV acquisitions in Côte d’Ivoire, Mali and Senegal.

**Design:**

HIV transmission dynamic models among the overall population and key populations [female sex workers (FSW), their clients, and MSM].

**Methods:**

Models were independently parameterized and calibrated for each set of country-specific demographic, behavioural, and epidemiological data. We estimated the fraction of new HIV infections over 2012–2021 averted by condom use and antiretroviral therapy (ART) uptake among key populations and non-key populations, the direct and indirect contribution of specific groups to new infections [transmission population-attributable fraction (tPAF)] over 2012–2021 due to prevention gaps, and the distribution of undiagnosed people with HIV (PWH) by risk group in January 2022 and their tPAF over 2022–2031.

**Results:**

Condom use and ART may have averted 81–88% of new HIV infections over 2012–2021 across countries, mostly due to condom use by key population. The tPAF of all key populations combined over 2012–2021 varied between 27% (Côte d’Ivoire) and 79% (Senegal). Male key populations (clients of FSW and MSM) contributed most to new infections (>60% in Mali and Senegal) owing to their higher HIV prevalence and larger prevention gaps. In 2022, men represented 56% of all PWH with an undiagnosed infection in Côte d’Ivoire (male key populations = 15%), 46% in Mali (male key populations = 23%), and 69% in Senegal (male key populations = 55%). If HIV testing and ART initiation rates remain at current levels, 20% of new HIV infections could be due to undiagnosed key populations living with HIV in Côte d’Ivoire over 2022–2031, 53% in Mali, and 65% in Senegal.

**Conclusion:**

Substantial HIV diagnosis gaps remain in Western Africa, especially among male key populations. Addressing these gaps is key to impacting the HIV epidemics in the region and achieving the goal of ending AIDS by 2030.

## Introduction

Almost a third of people with HIV (PWH) living in Western Africa had an undiagnosed infection in 2020 [1]. This is still far from the 95% diagnosis coverage target by 2025 set forth by the *Joint United Nations Programme on HIV/AIDS* (UNAIDS) [2]. In this region, diagnostic gaps are particularly pronounced for key populations, which include female sex workers (FSW), their clients, and MSM. Structural factors such as poverty, discrimination, and stigma are important barriers to uptake of HIV services [1,3–6] that create unmet HIV needs for key populations [7,8]. Until at least 2008, and apart from several successful research projects among FSW in the 1990s (e.g. Benin, Côte d’Ivoire, Senegal) [9,10], national HIV prevention programs in Western African countries have generally placed a strong emphasis on the general population, rather than key populations [11,12].

Côte d’Ivoire, Mali, and Senegal are three Western African countries where members of key populations are important to the overall HIV transmission dynamics [10,12,13]. The levels of discrimination and stigma that key populations experience are generally high but vary across key populations and countries [3,14,15]. Sex work is legal in all three countries and even regulated by the authorities in Senegal [16], whereas sex between men is legal in Côte d’Ivoire and Mali, but criminalized and severely punished in Senegal [17–19]. The three countries have progressively integrated HIV prevention programs, including condom distribution, among vulnerable groups in their national plans, initially among FSW, MSM only from the mid-2000s [20,21], but few programs and reliable surveys exist for FSW clients.

Although overall HIV prevalence has declined to relatively low levels in Western Africa (1% in 2023), it remains nine-fold higher among key populations compared to the overall population [8,12,22]. HIV prevalence among FSW has sharply decreased in these countries [23–25], partly due to successful condom promotion [25–27]. Nevertheless, higher HIV-acquisition risks persist among key populations compared to non-key populations. For example, HIV prevalence among MSM in Senegal appears to have increased from 18% in 2014 [28] to 28% in 2017 [29] (compared to 0.3% among all adult men in 2023 [30]). Quantifying HIV diagnosis gaps among different risk groups help identify among whom to strengthen the HIV care continuum to help achieve epidemic control [31–33].

We conducted a mathematical modelling analysis estimating the population-level impact of condom use and antiretroviral therapy (ART) on HIV acquisitions and deaths in Côte d’Ivoire, Mali and Senegal over 2012–2021, the contribution of different groups to new HIV acquisitions and transmissions over successive decades, and the gaps in HIV diagnosis among selected groups in 2022 and their contribution to new acquisitions over 2022–2031.

## Methods

### Mathematical model of HIV transmission and control

A previously published deterministic compartmental model of HIV transmission in Côte d’Ivoire [10,34] was adapted for our analysis and parametrized and calibrated to country-specific demographic, behavioural, HIV epidemiological and intervention data over time for Côte d’Ivoire, Mali, and Senegal separately. The model simulates the HIV epidemic over 1980–2031. Details of model, data sources, and fitting are given in Supplementary File 1.

The model represents an open and growing population stratified in eight risk groups including FSW, their clients (‘clients’), men who have sex with male and female partners (MSMW) and with male partners exclusively (MSME), and the remaining non-key population female and male populations stratified in low risk/intermediate risk (based on their number of partners). Despite experiencing lower levels of stigma and vulnerability compared with FSW and MSM, clients were classified as key population in our study because of their disproportionate contribution to new HIV transmissions in sub-Saharan Africa [10,35–37]. Each risk group is further divided into four age groups: 15–19, 20–24, 25–49, and 50–59 years old. People enter the model at age 15 years as sexually naive or active, or to the 24–49-years-old age group (migration). Three types of partnership (heterosexual non-commercial and commercial, and between men) are explicitly modelled alongside risk and age sexual mixing. FSW can only form partnerships with clients (Table S1b and Figure S1a).

People acquire HIV at an annual per-capita rate force of infection, which depends on per-sex-act transmission probabilities by sex (we assumed higher male-to-female and male-to-male HIV transmission risks than female-to-male) and disease stage, number of sexual partners, age/risk group mixing, number and types of sex acts per partnership type (time-specific, risk-specific, and age-specific) fraction of acts involving condoms, HIV prevalence, and levels of HIV viral suppression among partners. People with an untreated HIV infection progress through five infection stages (Figure S1c). HIV testing of both people not living with HIV and PWH, and treatment of PWH are explicitly modelled (Figure S1d), with diagnosed PWH initiating ART, increasing their life expectancy and reducing infectivity. Condom use and HIV testing rates reflected empirical trends over time and were assumed to remain constant after the last data point available, which generally resulted in slow increases in the fraction of PWH on ART and virally suppressed (who cannot transmit HIV) in most risk and age groups until 2031. We assumed that condom use reduced per-act HIV transmission risk by 75–94% [38] and up to 25% overreporting of condom use by FSW [38–40]. The marginal use of HIV preexposure prophylaxis (PrEP) in the countries was not modelled [41].

### Data sources and model calibration

The demographic, epidemiological, and HIV intervention parameters and fitting outcomes were informed by available country-specific data sources (Tables S2a,b,c). Fitting outcomes, stratified by age, sex, and groups, included: HIV prevalence and incidence, annual number of new HIV infections and HIV-related deaths, fraction of people ever tested, fraction of all PWH with a diagnosed infection, on ART, with a suppressed viral load, and the annual number of total and positive HIV tests. Demographic information (growth rate, age distribution, deaths rates) was sourced from the United Nations Population Division (2019 revision) [42]. The size of each key population reflected available county-specific empirical estimates (Table S1a). Sexual behaviours (including condom use), HIV testing rates, and HIV prevalence of overall and non-key populations over time were informed by nationally representative household-based surveys from 1992 to 2018 (e.g. Demographic Health Surveys [43], Multiple-Indicator Cluster Surveys [44], Population-based HIV Impact Assessment (PHIA) [45]). UNAIDS’ Shiny90 [46] estimates informed the fraction of people ever tested (by HIV status) and the fraction of PWH diagnosed. Finally, biological parameters reflecting HIV transmissibility, disease progression, and the efficacy of condom use were abstracted from published literature (Table S1c).

For each country, the model was fitted using a Bayesian framework, which triangulated all data sources while considering uncertainties in both parameters and outcome data (Supplementary File 1 p34). We selected a first subset of parameter sets from 50 million simulations (generated from a Latin Hypercube sample) that produced predictions agreeing with predefined ranges of the different fitting outcomes. Then, the final posterior parameter sets (used as the base case scenario) included the 100 simulations with the highest likelihood from the first subset of simulations.

### Impact of condom use and antiretroviral therapy uptake on HIV infections and deaths

We calculated the fractions of new HIV infections and deaths directly and indirectly averted by condom use, ART uptake, and both among the overall population and specific risk groups over different time periods [*t*_0_, *t*_1_] (indicator $AF_{t_{0},t_{1}}$) [31] as the relative difference between the cumulative number of infections between the base-case and a counterfactual scenario without one or both interventions over [*t*_0_, *t*_1_] (details in Supplementary File 1, p49).

### Contributions to HIV acquisitions, to direct HIV transmissions only, and to both direct and indirect HIV transmissions (transmission population-attributable fraction)

We derived the distribution of acquired infections and of directly transmitted infections as the fractions of all new cumulative infections over time period [*t*_0_, *t*_1_] which are acquired by a specific risk group and which are directly transmitted by a specific risk group, respectively. Finally, the transmission population-attributable fraction ($\text{tPA}{\text{F}}_{t_{0},t_{1}}$) [31], which captures both direct and indirect transmissions, is the relative difference between the cumulative number of new infections in the base-case scenario and a counterfactual scenario where the specific relevant risk group cannot transmit HIV to any partners (equations in Supplementary File 1). The tPAF can be interpreted as the fraction of all new infections averted by an intervention blocking all transmissions from the relevant risk group (but not HIV acquisitions by this group).

### Magnitude of HIV diagnosis/treatment gaps and associated transmission population-attributable fractions

We estimated the fractions (i.e. prevalence) and population distributions in January 2022, as well as 10-year tPAFs of PWH in each risk group with an undiagnosed infection, a diagnosed but untreated infection, and a treated infection. We also estimated the 10-year tPAFs of PWH who have never tested for HIV (even before living with HIV).

All indicators were calculated over successive decades between 1992 and 2031. We report median and 90% uncertainty interval (UI, 5th and 95th percentiles of the distribution) of model predictions across the 100 posterior parameter sets.

## Results

### Model fits to empirical data

Our models reproduced available empirical demographic, epidemiological, and HIV intervention data across time, age, and risk groups (Tables S2a-c, Figures S2-4), although data triangulation highlighted several discrepancies between low fractions of PWH reporting ever having tested [47], and high fractions of PWH virally suppressed for the same population. Similar to the UNAIDS estimates, modelled national HIV prevalence was highest in Côte d’Ivoire (2.0% in 2022, 0.8% in Mali, and 0.3% in Senegal, Table 1), whereas prevalent infections were more concentrated among key populations in Mali and Senegal (Table 1, Figures S2–S4). Predicted HIV prevalence has decreased overall and among all risk groups except among MSM in Senegal where it remained stable (Figure S4g). ART coverage in 2022 was generally lower among key populations PWH than non-key populations PWH, especially among MSM in Senegal (Table 1).

**Table 1:** Summary of key model estimates of (a) size of key populations in 2022 relative to the total sex-specific population, (b) HIV prevalence in 2012 and 2022, (c) coverage of ART in 2012 and 2022, and (d) fractions of sex acts across all partnerships modelled that involved condoms in 2022. Median and 90% UI (5th and 95th percentiles) of estimates are shown.

Risk groups	Côte d’Ivoire		Mali		Senegal	
(a) Relative population size in 2022						
FSW (% of all women)	1.4% (0.9–1.9)		0.6% (0.4–0.9)		0.6% (0.5–0.8)	
Clients of FSW (% of all men)	13.9% (8.4–18.4)		10.8% (5.1–18.1)		4.2% (2.6–7.2)	
MSM (% of all men)	1.3% (0.9–1.6)		0.4% (0.2–0.5)		0.5% (0.3–0.9)	
Key populations^a^	8.8% (5.4–11.3)		5.8% (2.9–9.4)		2.6% (1.8–4.2)	
Non-key populations	91.2% (88.7–94.6)		94.2% (90.6–97.1)		97.4% (95.8–98.2)	
(b) HIV prevalence						
	2012	2022	2012	2022	2012	2022
All	3.4% (2.9–4)	2.1% (1.7–2.5)	1% (0.7–1.2)	0.6% (0.4–0.8)	0.5% (0.4–0.5)	0.3% (0.3–0.4)
FSW	12% (9–16)	8% (6–11)	15% (13–19)	8% (7–11)	7% (5–11)	4% (2–6)
Clients of FSW	4% (3–6)	2% (2–4)	4% (2–6)	2% (1–3)	3% (2–5)	2% (1–3)
MSM	15% (10–21)	7% (5–11)	22% (17–27)	13% (9–16)	19% (14–26)	22% (15–28)
Key populations^a^	6% (4–8)	3% (2–4)	5% (3–8)	3% (2–4)	5% (3–8)	4% (2–6)
Non-key populations	3.4% (2.9–4.0)	2.1% (1.7–2.5)	1% (0.7–1.2)	0.6% (0.4–0.8)	0.5% (0.4–0.5)	0.3% (0.3–0.4)
(c) ART coverage among people with HIV						
	2012	2022	2012	2022	2012	2022
All	39% (35–42)	67% (64–69)	27% (25–31)	46% (43–51)	51% (46–55)	68% (63–71)
FSW	38% (31–44)	64% (57–75)	40% (32–55)	49% (37–63)	30% (23–37)	50% (41–59)
Clients of FSW	35% (31–42)	56% (52–61)	26% (20–33)	38% (32–46)	49% (37–58)	70% (61–77)
MSM	26% (18–36)	52% (40–60)	47% (36–55)	56% (44–67)	20% (13–24)	28% (20–36)
Key populations^a^	34% (29–40)	57% (53–61)	31% (27–37)	44% (38–51)	35% (29–40)	45% (37–51)
Non-key populations	40% (36–43)	68% (66–70)	26% (23–29)	47% (44–52)	58% (52–63)	78% (75–82)
(d) Fraction of sex acts involving condom use in 2022						
Partnerships between non-key populations^b^	16% (9–24)		3% (1–5)		1% (1–2)	
During sex work^c^	75% (63–88)		82% (69–96)		78% (64–90)	
During sex between men^c^	74% (68–81)		76% (70–81)		77% (71–83)	

ART, antiretroviral therapy; FSW, female sex workers.

a Key populations, female sex workers, their clients (‘clients’), and MSM combined.

b Here, shown for the case where both partners are aged between 25 and 49 years old.

c Condom use during sex between MSM and their female partners and between clients and their non-FSW partners are assumed to be similar than between non-key population partners.

### Impact of interventions on HIV outcomes averted fractions

We estimated that the combined use of condoms and ART may have directly and indirectly averted 81–88% of all new HIV infections and 45–64% of all HIV deaths over 2012–2021 across countries. An estimated 74% (90% UI: 65–82%) of all new HIV infections may have been averted by all condom use in Côte d’Ivoire, 75% (66–85%) in Mali, and 82% (74–87%) in Senegal (Table 2, Figures S5a and b of Supplementary File 2), mainly due to high condom use by FSW and clients with all their partners (who report >90% of condom use at last sex [25–27,48–50] although we assumed a 25% overreporting, Table S1d). Comparatively, ART may have averted 45% (30–50%) of all new HIV infections in Côte d’Ivoire over 2012–2021, 27% (22–34%) in Mali, and 48% (43–54%) in Senegal, reflecting the slower increases in ART coverage over the same period (Table 1). The estimated population-level impacts of condom use and ART are presented with more details in the supplement.

**Table 2:** Impact of condom use and ART uptake on HIV outcomes over 2012–2021. Estimated fractions of (a) new HIV infections and (b) HIV-related deaths over 2012–2021 averted by the intervention used by all or by specific risk groups (with all their partners for condoms). Median and 90% UI (5th and 95th percentiles) of estimates are shown.

	Averted fractions by condom use			Averted fractions by ART			Averted fractions by condom use and ART		
Risk groups	Côte d’Ivoire	Mali	Senegal	Côte d’Ivoire	Mali	Senegal	Côte d’Ivoire	Mali	Senegal
(a) New HIV infections over 2012–2021									
All	74% (65–82)	75% (66–85)	82% (74–87)	45% (39–50)	27% (22–34)	48% (43–54)	82% (78–87)	81% (74–89)	88% (83–91)
FSW	71% (61–80)	74% (64–84)	79% (68–86)	3% (1–7)	8% (5–13)	7% (3–12)	74% (64–83)	78% (69–87)	82% (73–87)
Clients	72% (62–81)	75% (64–84)	79% (69–86)	7% (4–12)	9% (5–14)	20% (13–28)	75% (64–83)	76% (67–85)	83% (75–88)
MSM	10% (6–17)	15% (9–23)	43% (29–54)	2% (0.7–3)	4% (2–7)	10% (6–16)	15% (9–23)	21% (12–30)	47% (32–58)
KP	73% (63–82)	75% (66–85)	82% (74–87)	11% (7–18)	19% (13–26)	32% (25–40)	78% (69–85)	81% (72–88)	87% (82–90)
Non-KP	14% (11–18)	1% (0.9–2)	0.7% (0.5–1)	40% (32–46)	12% (9–19)	29% (19–38)	49% (42–54)	14% (10–20)	30% (20–39)
(b) New HIV deaths over 2012–2021									
All	22% (14–33)	19% (13–32)	30% (19–44)	40% (31–48)	29% (21–37)	48% (35–55)	55% (46–62)	45% (37–53)	64% (54–71)
FSW	19% (12–31)	18% (12–31)	22% (12–38)	2% (0.9–3)	3% (2–4)	4% (2–6)	23% (15–34)	23% (17–36)	28% (18–44)
Clients	20% (12–32)	18% (12–31)	22% (12–38)	5% (3–8)	6% (4–11)	11% (6–17)	27% (19–39)	25% (18–37)	35% (25–50)
MSM	1% (0.7–3)	2% (0.8–3)	12% (7–20)	1% (0.6–3)	2% (1–4)	6% (3–11)	3% (2–7)	5% (3–7)	19% (12–30)
KP	21% (13–32)	19% (13–32)	30% (19–44)	8% (4–12)	11% (8–17)	20% (11–26)	33% (24–45)	32% (26–43)	50% (38–59)
Non-KP	2% (1–3)	0.2% (0.1–0.3)	0.1% (0.1–0.1)	38% (28–45)	22% (14–30)	38% (28–47)	39% (30–47)	22% (14–30)	38% (28–47)

ART, antiretroviral therapy; Clients, clients of female sex workers; FSW, female sex workers; KP, key populations: FSW, their clients, and MSM combined; MSM, men who have sex with men.

### Fraction of new HIV infections acquired, directly transmitted and transmission population-attributable fraction

**Fig. 1:**
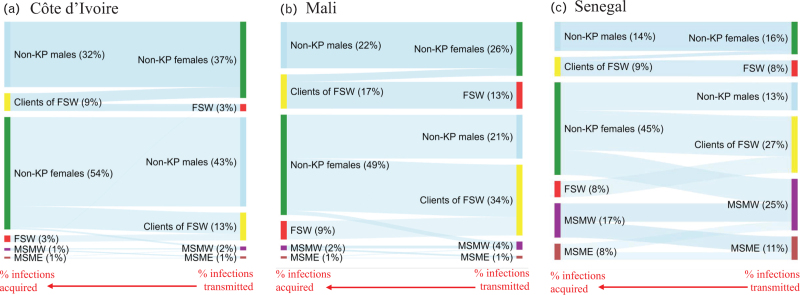
Median fraction of new HIV infections which were acquired (left side of panels) and directly transmitted (right side of panels) by different risk groups in (a) Côte d’Ivoire, (b) Mali, and (c) Senegal over 2012–2021.

Our model predicted that women acquired more new infections (median: 52–58% across countries) than they directly transmitted (22% in Senegal, and ∼40% in Côte d’Ivoire and Mali) over 2012–2021 (Fig. 1, Table S3).

The width of the light blue bands is proportional to the fraction of HIV infections acquired and transmitted over the period. Estimates for Mali should be interpreted with caution as no estimates of HIV prevalence or antiretroviral therapy (ART) coverage was available for clients of female sex workers (see discussion). FSW, female sex workers; MSMW, men who have sex with men and women; MSME, men who have sex with men exclusively; MSM, all men who have sex with men; KP, FSW and their clients, and MSM combined.

In Côte d’Ivoire, key populations (including clients) acquired a median 14% (9–20%) and directly transmitted 20% (12–32%) of all new infections over 2012–2021 and had a 10-year tPAF of 27% (16–40%; Table 3 and Fig. 1). The tPAF over 2012–2021 was highest for clients of FSW (20%; 12–35%) than FSW (7%; 3–13%) and MSM (3%; 2–6%). In Mali, key populations acquired an estimated 29% (17–42%) of all new HIV infections over 2012–2021, directly transmitted 54% (38–70%) of all infections, and had a 10-year tPAF of 63% (45–77%). The 10-year tPAF was particularly high for FSW and their clients, 24% (13–39%) and 50% (35–63%), respectively. Key populations in Senegal acquired 43% (32–55%) of all new HIV infections over 2012–2021, directly transmitted 72% (61–84%) of all new infections and had a 10-year tPAF of 79% (69–89%). The tPAF was highest for MSM and clients of FSW, 39% (24–58%) and 36% (20–52%), respectively, compared to FSW (17%; 8–31%). We estimated that MSMW directly or indirectly transmitted (10-year tPAF) a quarter of new infections occurring among all women in Senegal (Table S4c). The 10-year tPAFs of key populations remained relatively stable since the 2000s in the three countries, except in Senegal where they slightly increased (Figure S6).

**Table 3:** HIV diagnosis among PWH. Estimated fraction of PWH with an undiagnosed infection in January 2022, distribution of PWH with an undiagnosed infection in January 2022, and 10-year transmission population-attributable fraction by specific risk groups of PWH with an undiagnosed HIV infection over 2022–2031. Median and 90% UI (5th and 95th percentiles) of outcomes are shown. The sum of median estimates for different risk groups may not sum to 100% exactly.

	Fraction of PWH with an undiagnosed infection (2022)	Distribution of PWH with undiagnosed infections by risk group (2022)^a^	Transmission PAF by PWH with undiagnosed infection (2022–2031)
Côte d’Ivoire			
All	25% (22–28)	100% (100–100)	67% (60–75)
All women	18% (15–21)	44% (38–51)	36% (30–41)
All men	37% (32–41)	56% (49–62)	55% (49–64)
FSW	31% (22–39)	3% (2–5)	5% (2–11)
Clients of FSW	37% (32–42)	11% (7–18)	15% (8–26)
MSM	44% (37–57)	4% (2–7)	2% (1–5)
Key populations	38% (33–42)	18% (13–27)	20% (11–31)
Non-key populations women	18% (14–21)	40% (35–48)	31% (24–39)
Non-key populations men	36% (32–41)	40% (33–48)	39% (27–51)
Mali			
All	48% (43–51)	100% (100–100)	83% (78–88)
All women	42% (38–44)	54% (46–61)	44% (39–54)
All men	58% (50–63)	46% (39–54)	69% (62–74)
FSW	45% (33–60)	4% (2–8)	18% (9–35)
Clients of FSW	58% (49–64)^b^	20% (12–33)	42% (26–56)
MSM	40% (30–51)	3% (2–6)	6% (3–12)
Key populations	51% (46–58)	29% (17–41)	53% (35–67)
Non-key populations women	42% (37–44)	49% (41–57)	27% (19–35)
Non-key populations men	62% (54–68)	23% (16–30)	23% (10–38)
Senegal			
All	22% (18–27)	100% (100–100)	74% (66–81)
All women	11% (9–15)	31% (21–40)	15% (10–23)
All men	38% (31–43)	69% (60–79)	70% (61–78)
FSW	28% (18–41)	5% (2–9)	7% (2–14)
Clients of FSW	23% (16–33)	11% (5–21)	14% (6–29)
MSM	57% (41–71)	44% (25–62)	50% (31–64)
Key populations	42% (34–51)	60% (46–74)	65% (54–75)
Non-key populations women	10% (7–13)	25% (17–35)	8% (5–13)
Non-key populations men	24% (17–33)	15% (8–23)	6% (3–13)

FSW, female sex workers; key populations, FSW, their clients, and MSM combined; MSM, men who have sex with men; PWH, people with HIV

a Takes into account the size of the risk population as well as the fraction of PWH in the relevant subgroup with undiagnosed infection.

b Estimates for Mali should be interpreted with caution as no estimates of HIV prevalence or coverage of treatment among clients of FSW was available in the country (see discussion).

Finally, key populations and their sexual partners acquired 25% (15–40%) of new HIV infections over 2012–2021 in Côte d’Ivoire, 58% (41–74%) in Mali, and 74% (63–85%) in Senegal (not shown).

### HIV diagnosis gaps in 2022

We estimated heterogeneous diagnostic gaps across countries in 2022. The highest overall median fraction of PWH with undiagnosed infection was in Mali (48%), followed by Côte d’Ivoire (25%) and Senegal (22%, Table 3). Women had smaller fractions of undiagnosed PWH then men (median differences ranged between 16%-points in Mali and 27%-points in Senegal). MSM in Senegal and Côte d’Ivoire had the largest fraction of undiagnosed infection in 2022 (median: 57 and 44%, respectively) compared with non-key population men (24 and 36%). In Mali, the fraction of undiagnosed infections among FSW and FSW clients were similar than among all women and all men, respectively.

The reduction in the fractions of undiagnosed infections between 2012 and 2022 was more pronounced for non-FSW women (from 47 to 18% in Côte d’Ivoire, from 26 to 10% in Senegal, and from 67 to 42% in Mali, Tables S5 and S6) and least pronounced for MSM in Mali and Senegal (from 47 to 40% and from 65 to 57%, respectively).

### Population distribution of people with HIV with an undiagnosed infection

Men accounted for medians of 69, 56, and 46% of all PWH with an undiagnosed infection in 2022 in Senegal, Côte d’Ivoire, and in Mali, respectively (Table 3). Key populations accounted for most PWH with an undiagnosed infection in Senegal (60%, including 44% from MSM) compared with 18 and 29% for Côte d’Ivoire and Mali, respectively, mostly from clients of FSW. The estimated fractions of PWH with a diagnosed infection who were on ART were similar across countries and risk groups (87–90%, Table S5).

### Transmission population-attributable fraction (tPAF) of people with undiagnosed infection

**Fig. 2:**
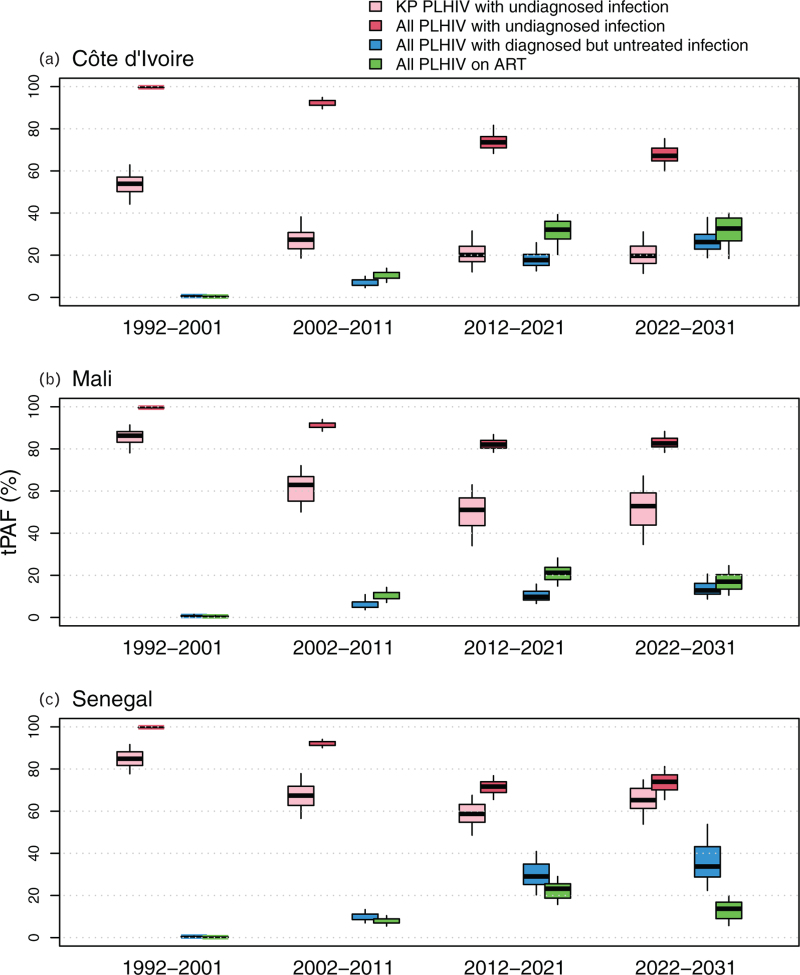
Transmission population-attributable fraction (direct and indirect transmission) estimated for people with HIV of different diagnosis and treatment stages.

If HIV testing and ART initiation rates remain at the current levels over 2022–2031, the predicted median tPAF of PWH with an undiagnosed infection will be 67% (60–75%) in Côte d’Ivoire, 83% (78–88%) in Mali, and 74% (66–81%) in Senegal over 2022–2031, which is comparable to the 2012–2021 estimates (Fig. 2, Table S6). In particular, the tPAF of PWH who have never been tested (even before living with HIV) over 2022–2031 was 47% (39–57%) in Côte d’Ivoire, 65% (59–73%) in Mali, and 36% (27–47%) in Senegal (not shown).

Estimated transmission population-attributable fraction (tPAFs) of members of key populations (KP) living with HIV with undiagnosed infection (pink boxes and intervals), All PWH with undiagnosed infection (red), with diagnosed infection but not on antiretroviral therapy (ART) (blue), and on ART (green) over successive decades. Boxes represent median and interquartile range, whereas error bars represent 90% uncertainty interval (UI) (5th and 95th percentiles) of tPAF estimates across all posterior parameter sets. KP include female sex workers, their clients, and MSM.

The tPAF of key populations with an undiagnosed infection over 2022–2031 were higher in Mali (53%; 35–67%) and Senegal (65%; 54–75%) than Côte d’Ivoire (20%; 11–31%) (Fig. 2, Table S6). Clients of FSW with an undiagnosed infection had the highest predicted tPAF over 2022–2031 in Côte d’Ivoire (15%; 8–26%) and Mali (42%; 26–56%), compared with 50% (31–64%) for MSM in Senegal.

The tPAF of PWH with a diagnosed but untreated infection increased over time, up to 26% (19–38%) in Côte d’Ivoire, 13% (9–21%) in Mali, 34% (22–54%) in Senegal over 2022–2031 (Fig. 2) as the relative size of this population among all untreated PWH increased over time. Finally, as the fraction of PWH on ART and with a suppressed viral load increased over time, the tPAFs of treated PWH with an unsuppressed viral load also increased over time, and plateaued in 2020s onward between a median of 14% (in Senegal) and 33% (in Côte d’Ivoire, Fig. 2). The Figure S7 illustrates the dependence of the contribution to future new HIV infections on our UNAIDS-informed fraction of PWH with a suppressed HIV viral load.

## Discussion

Our analysis highlights the importance of addressing the current gaps in HIV diagnosis, especially among FSW, their clients, and MSM, to further decrease HIV incidence and mortality in Western Africa. Over two-thirds of new HIV infections over the next decade could be contributed by PWH with an undiagnosed infection, including around half by PWH who have never tested for HIV. However, the risk groups contributing the most to new acquisitions and transmissions varied across countries, stressing the importance of country-specific information (among key populations and non-key populations) to tailor interventions improving HIV treatment coverage and reducing HIV incidence in the region.

We found substantial gaps in HIV diagnosis in the three countries, especially in Mali where barely half of PWH were estimated to have a diagnosed infection in 2022. Median diagnosis coverage was consistently higher among women than men across countries (by at least 15%-points). PWH with an undiagnosed infection include a disproportionate number of members of key populations, especially in Senegal (almost half) despite their relatively small population size.

The estimated tPAFs of risk groups differed from the distributions of PWH with an undiagnosed infection. Transmission probabilities between men and from men to women are higher than from women to men whereas tPAFs of key populations were relatively large also because they have higher number of partners and are often less frequently virally suppressed. Importantly, our study suggests that the distributions of acquired and transmitted infections for the last decade may remain qualitatively similar for the next decade unless HIV prevention/treatment gaps are reduced among key populations.

Our analysis underscores differences in HIV epidemics across the three countries studied. Fewer new HIV infections in Côte d’Ivoire are contributed by FSW and MSM than in Mali and Senegal, although clients of FSW still contribute to a fifth of new HIV infections in Côte d’Ivoire. This was qualitatively consistent with a study from Maheu-Giroux [10] where the tPAF of FSW clients over 2005–2015 was 45%.

In Mali, the HIV epidemic appeared to still be strongly influenced by sex work, with large fractions of new HIV infections being contributed by FSW and their clients. However, our estimates should be interpreted with caution given the lack of specific HIV prevalence and ART coverage estimates for FSW clients.

Our tPAF estimates of MSM and clients of FSW (39 and 36% over 2012–2021) for Senegal, were similar to recent estimates for Dakar only, where the tPAF of sex between men over 2005–2015 was 42%, and 20% for non-commercial sex for FSW and clients [13]. The large fractions (over 1/3) of new HIV infections estimated to have been acquired by MSM, despite the relatively small size of the population, suggests that scaling-up PrEP in this population could meaningfully accelerate the decrease in HIV incidence in the country.

Similar to other modelling studies of HIV in the region [9,13,37], our analysis underlines the success of past interventions against HIV. Approximately three-quarters of new HIV infections over the last decade may have been prevented only by condom use by key populations. Maintaining outreach and high levels of condom use among key populations remains important, even in Côte d’Ivoire where the epidemic appears to have progressed among non-key populations. These findings concur with recent modelling studies [36,37,51].

Our study has limitations, which highlights key data gaps on the HIV prevalence and levels of treatment among clients of FSW. Few surveys have been carried out in the countries modelled, and none in Mali, which required additional assumptions. Data from Côte d’Ivoire and non-key populations data from Mali were used to inform the number of paying and non-paying partners of clients in Mali and the rates of HIV testing and treatment initiation, respectively (Table S1e). Despite being challenging to collect [52–54], such data is needed as clients may contribute to large fractions (30–40%) of new HIV infections in sub-Saharan Africa [10,36,37]. Desirability bias in reporting of sexual–behavioural data or condom use was addressed by accounting for uncertainty in data and over-reporting of condom use by FSW, reducing the risk of overestimating the impact of condom use by FSW [39]. However, no quantification of potential overreporting of condom use by MSM was available and our analysis might slightly overestimate the impact of condom use by MSM and slightly underestimate the tPAFs of MSM.

Our study is based on exhaustive reviews of country-specific data since the 1980s, including recent integrated biological and behavioural assessment (IBBS) surveys among key populations (2020) [23,24,55,56], allowing to model the plausible past and future dynamics of the HIV epidemic. Models were fitted to all available data on levels of HIV diagnosis and treatment among key and non-key populations including the fraction of PWH who have ever been tested for HIV, with a diagnosed infection, or with a suppressed viral load, which improved model predictions. Our Bayesian fitting method reflected uncertainties in both parameter and fitting data and allowed to triangulate different data sources. We identified discrepancies between different empirical HIV cascade estimates such as larger fractions of PWH suppressed HIV viral load than reporting ever being diagnosed by the same risk group because of under-reporting of HIV status (often influenced by HIV stigma) [47]. Our analysis distinguished and highlighted differences between indicators of HIV acquisition, direct HIV transmission, and of direct and secondary transmission (10-year tPAFs) when measuring contributions of risk groups. This is important because diagnosing and treating PWH prevent further transmission from them, which is difficult to observe and measure empirically.

There is an urgent need to address gaps in HIV diagnosis in West Africa, particularly among clients of FSW and MSM, which would not only improve equity in HIV treatment but also further curb HIV incidence in the region. Maintaining access and distribution of condoms, particularly for key populations, is still relevant. Scaling-up up the access to well tolerated and anonymous HIV testing through non-conventional modalities such as HIV self-tests and empowered community organizations (alongside national care providers) [57,58] may be an effective way to improve the coverage of HIV diagnosis in the context of stigma towards PWH and key populations.

## Acknowledgements

The authors would like to thank August Eubanks and Bruno Spire from the Aix Marseille University, and Christian Laurent from the French National Research Institute for Sustainable Development for providing insights on the CohMSM cohort in Côte d’Ivoire, Mali, Burkina Faso, and Togo, as well as the CohMSM cohort participants.

Authors’ contributions: M.M.G. and R.S. co-developed the HIV transmission model used for this study, with input from M.-C.B. and J.L. M.M.G. and N.S. performed data reviews for each country, with additional support from each country's representatives. C.D.-A., A.V., E.E., P.V., and C.M. contributed to additional data used in model parameterization and calibration. R.S. wrote the first manuscript version, accessed, and verified the data reported in the study. M.M.G., M.-C.B., A.S.F., A.V., P.V., and J.L. made substantial contributions to the interpretation of the results and edited the manuscript. All authors reviewed manuscript drafts, read, and approved the final version of the manuscript.

Composition of the ATLAS team study group:

Elvis Georges Amani, Kéba Badiane, Céline Bayac, Anne Bekelynck, Marie-Claude Boily, Sokhna Boye, Guillaume Breton, Marc d’Elbée, Alice Desclaux, Annabel Desgrées du LoÛ, Papa Moussa Diop, Eboi Ehui, Graham Medley, Kévin Jean, Abdelaye Keita, Arsène Kra Kouassi, Odette Ky-Zerbo, Joseph Larmarange, Mathieu Maheu-Giroux, Raoul Moh, Rosine Mosso, Cheikh Tidiane Ndour, David Paltiel, Dolorès Pourette, Nicolas Rouveau, Romain Silhol, Arlette Simo Fotso, Fern Terris-Prestholt, Métogara Mohamed Traoré, Clémence Doumenc-Aïdara, Olivier Geoffroy, Odé Kanku Kabemba, Anthony Vautier, Armand Abokon, Camille Anoma, Annie Diokouri, Blaise Kouamé, Venance Kouakou, Odette Koffi, Alain Kpolo, Josiane Tety, Yacouba Traore, Jules Bagendabanga, Djelika Berthé, Daouda Diakité, Mahamadou Diakité, Youssouf Diallo, Minta Daouda, Septime Hessou, Saidou Kanambaye, Abdul Karim Kanouté, Bintou Dembélé Keita, Dramane Koné, Mariam Koné, Almoustapha Maiga, Aminata Saran Keita, Fadiala Sidibé, Madani Tall, Adam Yattassaye Camara, Abdoulaye Sanogo, Idrissa Bâ, Papa Amadou Niang Diallo, Fatou Fall, NDèye Fatou NGom Guèye, Sidy Mokhtar Ndiaye, Alassane Moussa Niang, Oumar Samba, Safiatou Thiam, Nguissali M.E. Turpin, Seydou Bouaré, Cheick Sidi Camara, Brou Alexis Kouadio, Sophie Sarrassat, Souleyman Sow, Agnes Eponon Ehua, Amélé Kouvahe, Marie-Anne Montaufray, Pauline Dama Ndeye.

Funding: this work was supported by Unitaid (Grant Number: 2018–23 ATLAS) through a collaborative agreement with Solthis. R.S. and M.C.B. acknowledge funding from the MRC Centre for Global Infectious Disease Analysis (reference MR/X020258/1), funded by the UK Medical Research Council (MRC). This UK-funded award is carried out in the frame of the Global Health EDCTP3 Joint Undertaking. P.V., M.C.B. and M.M.G. acknowledge funding from the Wellcome Trust (WT 226619/Z/22/Z). M.M.G.'s research program is funded by a *Canada Research Chair* (Tier 2) in *Population Health Modeling*. For the purpose of open access, the author has applied a Creative Commons Attribution (CC BY) license to any author-accepted manuscript version arising.

### Conflicts of interest

There are no conflicts of interest.

## Supplementary Material
