# Supplementary material for: The impact of past HIV interventions and diagnosis gaps on new HIV acquisitions, transmissions, and HIV-related deaths in Côte d’Ivoire, Mali, and Senegal
Source: AIDS. 2024 Jul 1;38(12):1783–93. doi: 10.1097/QAD.0000000000003974 (PMC11356671; doi:10.1097/QAD.0000000000003974)
Supplement: Supplementary file 1 [file aids-38-1783-s001.pdf]

The impact of past HIV interventions and diagnosis gaps on new HIV acquisitions, transmissions, and HIV-related deaths in Côte d'Ivoire, Mali, and Senegal. Model description.

Contents

Methods: model description ... ***3***

Overview ... **3**

Model equations ... **4**

Model initiation (1970) ... 4

Seeding of HIV in the population ... 6

Population recruitment of sexually naïve populations ... 6

Recruitment of sexually active population ... 7

Turnover in sex-work ... 12

Non-HIV mortality ... 12

Population ageing ... 12

HIV infection ... 14

HIV disease progression and mortality ... 26

HIV conventional testing and diagnosis ... 27

HIV treatment initiation and drop-out ... 33

Model fitting overview ... **34**

Model fitting data ... **34**

Fitting data (Côte d'Ivoire) ... 34

Fitting data (Mali) ... 40

Fitting data (Senegal) ... 44

Definition of indicators ... **49**

Results: model fits (Côte d'Ivoire) ... ***50***

Demography ... **50**

HIV epidemiology ... **52**

HIV treatment cascade ... **56**

Results: model fits (Mali) ... ***63***

Demography ... **63**

HIV epidemiology ... **65**

HIV treatment cascade ... **68**

Results: model fits (Senegal) ... ***74***

Demography ... **74**

HIV epidemiology ... **77**

HIV treatment cascade ... **81**

References ***..*** ***88***

Methods: model description

This model description was adapted from Maheu-Giroux et al. [1] and from Silhol et al.[2].

Overview

The deterministic compartmental model used for this analysis was adapted from a previously published model of HIV transmission in Côte d'Ivoire[1,3,4] and coded in C++. Our model was fitted to country-specific demographic, behavioural, HIV epidemiological and intervention data in Côte d'Ivoire, Mali and Senegal over 1980-2020.

The sexually active modelled population is noted $X_{i,u}^{r,a}(t)$, where

- The subscript "*r*" corresponds to gender-risk groups combinations: low-risk females (*r*=0), intermediate-risk females (*r*=1), female sex worker (FSW, *r*=2), low-risk males (*r*=3), intermediate-risk males (*r*=4), clients of FSW (*r*=5), men who have sex with men and women (MSMW) (*r*=6), and men who have sex with men exclusively (MSME) (*r*=7). See **Figure S1a**.
- The subscript "*a*" corresponds to age group: 15-19 years olds (*a*=0), 20-24 years olds (*a*=1), 25-49 years olds (*a*=2), and 50-59 years olds (*a*=3). See **Figure S1b**.
- The subscript "*i*" corresponds to HIV infection status: susceptible (*i*=0), acute infection (*i*=1), chronic infection with CD4>500 cells/µL (*i*=2), with CD4 between 350 and 500 cells/µL (*i*=3), with CD4 between 200 and 350 cells/µL (*i*=4), and with CD4 <200 cells/µL (*i*=5). See **Figure S1c.**
- The subscript "*u*" corresponds to HIV testing/diagnosis/treatment status: never tested (*u*=0), ever tested and undiagnosed if PLHIV (*u*=1), had a reactive self-test but is not diagnosed (*u*=2), diagnosed by conventional test and without having had a reactive self-test) (*u*=3), diagnosed via a confirmatory test following a reactive self-test (*u*=4), treated (*u*=5), ever treated but dropped-out from treatment (*u*=6). See **Figure S1d**.
- Our equations also use the subscript "*g*", which corresponds to the population sex, with females (*g*=0), and males (*g*=1).

The sexually naïve population is noted $V_r(t)$, with everyone being assumed to be aged 15-19 year old, HIV uninfected, and never having tested for HIV.

Model equations

The model can be expressed as a set of ordinary differential equation reflecting changes in modelled number of sexually naïve (V_r) and sexually active individuals ($X_{i,u,s}^{r,a}$).

$$\frac{dV_r}{dt} = L_r(t) - G'_r(t) - Y'_r(t)$$

$$\frac{dX_{i,u}^{r,a}}{dt} = E_{i,u}^{r,a}(t) + J_{i,u}^{r,a}(t) + Y_{i,u}^{r,a}(t) + G_{i,u}^{r,a}(t) + D_{i,u}^{r,a}(t) + Q_{i,u}^{r,a}(t) + T_{i,u}^{r,a}(t) + U_{i,u}^{r,a}(t)$$

Where

- L_r represents recruitment of sexually naïve populations into the model
- $E_{i,u}^{r,a}$ represents the recruitment of sexually active population through sexual debut of sexually naïve populations or migration of 25-49 years old adults
- $J_{i,u}^{r,a}$ represents the turnover in sex-work between female sex workers and intermediate-risk females
- $Y_{i,u}^{r,a}$ represents non-HIV mortality among sexually active, and Y'_r among sexually naïve
- $G_{i,u}^{r,a}$ represents ageing among sexually active, and G'_r among sexually naïve
- $D_{i,u}^{r,a}$ represents HIV acquisition
- $Q_{i,u}^{r,a}$ represents HIV infection progression and mortality
- $T_{i,u}^{r,a}$ represents HIV conventional testing and diagnosis
- $U_{i,u}^{r,a}$ represents HIV treatment (ART) initiation and drop-out

Each of the components of the two equations above is described in the section that follows the characterization of the population at model initiation

Model initiation (1970)

The model starts in 1970 with a population of size N_0 (estimated by the United Nations Population Division (UNPD)) [5], and using the age-distribution for the year 1970. The initial population is assumed to be HIV uninfected (*i*=0) and having never tested for HIV (*u*=0, *s*=0).

The relative sizes of the KPs are constant over time from simulation start, and reflect estimates from country-specific surveys spanning over 1995-2020. As in [1], the risk-distribution of the sexually naïve population mirrors the one of the sexually active populations to keep the size of KP constant over time.

$X_{i,u}^{r,a}(1970) = 0$ if *i*=0 or *u*=0 or *s*=0

In 1970, the female population is distributed as follows:

1) Lower-risk females (*r*=0) of age *a* (sexually active and naïve):

$$\begin{cases} X_{0,0}^{0,a}(1970) = N_0\, PR_{Fem} PR_{A_a}(1 - PR_{FSW})(1 - PR_{IRF})\big(1 - Vir_0(1970)\big) \\ V_0(1970) = N_0\, PR_{Fem} PR_{A_a}(1 - PR_{FSW})(1 - PR_{IRF}) Vir_0(1970) \end{cases} \text{ if } a=0$$

$X_{0,0}^{0,a} = N_0\ PR_{Fem}PR_{A_a}(1 - PR_{FSW})(1 - PR_{IRF})$ if a>0

2) Intermediate-risk females (r=1) of age a (sexually active and naïve):

$$\begin{rcases} \begin{cases} X_{0,0}^{1,a}(1970) = N_0\ PR_{Fem}PR_{A_a}(1 - PR_{FSW})PR_{IRF}\big(1 - Vir_0(1970)\big) \\ V_1(1970) = N_0\ PR_{Fem}PR_{A_a}(1 - PR_{FSW})PR_{IRF}Vir_0(1970) \end{cases} \end{rcases} \text{ if } a=0$$

$X_{0,0}^{1,a} = N_0\ PR_{Fem}PR_{A_a}(1 - PR_{FSW})PR_{IRF}$ if a>0

3) Female sex workers (r=2) of age a (sexually active and naïve):

$$\begin{rcases} \begin{cases} X_{0,0}^{2,a}(1970) = N_0\ PR_{Fem}PR_{A_a}PR_{FSW}\big(1 - Vir_0(1970)\big) \\ V_2(1970) = N_0\ PR_{Fem}PR_{A_a}PR_{FSW}Vir_0(1970) \end{cases} \end{rcases} \text{ if } a=0$$

$X_{0,0}^{2,a}(1970) = N_0\ PR_{Fem}PR_{A_a}PR_{FSW}$ if a>0

Where PR_{Fem} is the proportion of females in the model, PR_{A_a} is the relative size of the age group a at model start, $Vir_0(1970)$ the fraction of females aged 15-19 years old that are sexually naïve in 1970 (this fraction being assumed to be equal to the fraction in the first data point), PR_{FSW} the fraction of FSW among all females, PR_{IRF} the fraction of intermediate-risk females among all non-KP females. Empirical studies suggested slightly higher proportions of FSW among adult females in Côte d'Ivoire, (e.g. > 1% in [6,7]) compared to Mali and Senegal.

In 1970, the male population is distributed as follows:

4) Lower-risk males (r=3) of age a (sexually active and naïve):

$$\begin{rcases} \begin{cases} X_{0,0}^{3,a}(1970) = N_0\ (1 - PR_{Fem})PR_{A_a}(1 - PR_{Cli})(1 - PR_{MSM})(1 - PR_{IRM})\big(1 - Vir_1(1970)\big) \\ V_3(1970) = N_0\ (1 - PR_{Fem})PR_{A_a}(1 - PR_{Cli})(1 - PR_{MSM})(1 - PR_{IRM})Vir_1(1970) \end{cases} \end{rcases} \text{ if } a=0$$

$X_{0,0}^{3,a}(1970) = N_0\ (1 - PR_{Fem})PR_{A_a}(1 - PR_{Cli})(1 - PR_{MSM})(1 - PR_{IRM})$ if a>0

5) Intermediate-risk males (r=4) of age a (sexually active and naïve):

$$\begin{rcases} \begin{cases} X_{0,0}^{4,a}(1970) = N_0\ (1 - PR_{Fem})PR_{A_a}(1 - PR_{Cli})(1 - PR_{MSM})PR_{IRM}\big(1 - Vir_1(1970)\big) \\ V_4(1970) = N_0\ (1 - PR_{Fem})PR_{A_a}(1 - PR_{Cli})(1 - PR_{MSM})\ PR_{IRM}Vir_1(1970) \end{cases} \end{rcases} \text{ if } a=0$$

$X_{0,0}^{4,a}(1970) = N_0\ (1 - PR_{Fem})PR_{A_a}(1 - PR_{Cli})(1 - PR_{MSM})PR_{IRM}$ if a>0

6) Clients of FSW (r=5) of age a (sexually active and naïve):

$$\begin{rcases} \begin{cases} X_{0,0}^{5,a}(1970) = N_0\ (1 - PR_{Fem})PR_{A_a}PR_{Cli}\big(1 - Vir_1(1970)\big) \\ V_5(1970) = N_0\ (1 - PR_{Fem})PR_{A_a}\ PR_{Cli}Vir_1(1970) \end{cases} \end{rcases} \text{ if } a=0$$

$X_{0,0}^{5,a}(1970) = N_0\ (1 - PR_{Fem})PR_{A_a}PR_{Cli}$ if a>0

7) Men who have sex with men and women (MSMW, r=6) of age a (sexually active and naïve):

$$\left\{\begin{matrix} X_{0,0}^{6,a}(1970) = N_0\ (1 - PR_{Fem})PR_{A_a}PR_{MSM}PR_{Bi}\big(1 - Vir_1(1970)\big) \\ V_6(1970) = N_0\ (1 - PR_{Fem})PR_{A_a}PR_{MSM}PR_{Bi}Vir_1(1970) \end{matrix}\right\} \text{ if } a\text{=0}$$

$X_{0,0}^{6,a}(1970) = N_0\ (1 - PR_{Fem})PR_{A_a}PR_{MSM}PR_{Bi}$ if a>0

7) Men who have sex with men exclusively (MSME, r=7) of age a (sexually active and naïve):

$$\left\{\begin{matrix} X_{0,0}^{7,a}(1970) = N_0\ (1 - PR_{Fem})PR_{A_a}PR_{MSM}(1 - PR_{Bi})\big(1 - Vir_1(1970)\big) \\ V_7(1970) = N_0\ (1 - PR_{Fem})PR_{A_a}PR_{MSM}(1 - PR_{Bi})Vir_1(1970) \end{matrix}\right\} \text{ if } a\text{=0}$$

$X_{0,0}^{7,a}(1970) = N_0\ (1 - PR_{Fem})PR_{A_a}PR_{MSM}(1 - PR_{Bi})$ if a>0

Where $(1 - PR_{Fem})$ is the proportion of males in the model, PR_{A_a} is the relative size of the age group a at model start, $Vir_1(1970)$ the fraction of males aged 15-19 years old that are sexually naïve in 1970 (again, this fraction being assumed to be equal to the estimate from each country first data point), PR_{IRM} the fraction of intermediate-risk males among all non-KP males, PR_{Cli} the fraction of FSW clients among all males, PR_{MSM} the fraction of MSM among all males, PR_{Bi} the fraction of MSM that ever had a female partner.

The fraction PR_{Cli} is calculated using the multiplier method[8], accounting for partner change rates reported by FSW and their clients, as well as the size of the FSW population[1]. As in [1,4], simulations were discarded when the estimated fraction PR_{Cli} was over 20%.

Seeding of HIV in the population

HIV is assumed to start spreading into a very small fraction of the modelled population between 1975 and 1979 (using a parameter HIV_{Start}). At that particular time point, fractions $Prev_{FSW}$, $Prev_{Cli}$, and $Prev_{MSM}$ of FSW, clients and MSM respectively are assumed to become infected by HIV (and all with a CD4>500 cells/µL) (see **Table S1a**).

Population recruitment of sexually naïve populations L_r

Recruitment of sexually naïve populations of each risk group (L_r) is determined at each time step using the following formula:

$$L_r(t) = \Bigg(Y'_r(t) + \sum\nolimits_{a,i,u} \mu_a X_{i,u}^{r,a}(t) + \sum\nolimits_{a,i>0,u\neq 5} \gamma_4 X_{i,u}^{r,a}(t) + \sum\nolimits_{a,i>0,s} \frac{\gamma_4}{RR_\omega} X_{i,5}^{r,a}(t) + \varepsilon'\left(\sum\nolimits_{a,i,u} X_{i,u}^{r,a}(t) + V_r(t)\right) + \sum\nolimits_{i,u} G_{i,u}^{r,3}(t)\Bigg) Vir_g(t)$$

Where Y'_r $(= \mu_0 V_r)$ and $\mu_a X_{i,u}^{r,a}$ are the number of sexually naïve and sexually active individuals exiting the model due to non-HIV mortality at each time step, γ_4 the rate at which PLHIV in the last stage of infection (<200 CD4 cells/μL) die from AIDS. The parameter RR_ω reflects the increase in survival among PLHIV on ART compared to PLHIV not on ART[9]. The term ε' is the population growth due to fertility and is calculated as $\varepsilon' = \varepsilon - (\chi PR\_A_2)$, where ε is the total population growth rate, χ the migration rate and PR_A_2 the fraction of people aged 25-49 years old in the model. As suggested by census data for Côte d'Ivoire, the large majority of immigrants are aged between 25 and 49 years[10]. The term $G_{i,u}^{r,3}$ is the number of people leaving the model having reached the age of 60 years old. Finally, the time-dependant parameter $Vir_g(t)$ is the fraction of female or male entering the 15-19 years old age group as sexually naïve and was informed using data from the countries successive DHS's.

Recruitment of sexually active population $E_{i,u}^{r,a}$

Sexually active individuals of each risk group are recruited at each time step through ageing of sexually naïve 15-19 years old or migration of sexually active 25-49 years old (which are assumed to have the same HIV prevalence as adults in Côte d'Ivoire but are assumed to have never tested for HIV).

$$E_{i=0,u=0}^{r,a=0} = \left(Y'_r(t) + \sum_{a,i,u} \mu_a X_{i,u}^{r,a}(t) + \sum_{a,i>0,u\neq 5} \gamma_4 X_{i,u}^{r,a}(t) + \sum_{a,i>0} \frac{\gamma_4}{RR_\omega} X_{i,5}^{r,a}(t) + \varepsilon'\left(\sum_{a,i,u} X_{i,u}^{r,a}(t) + V_r(t)\right) + \sum_{i,u} G_{i,u}^{r,3}(t)\right)\left(1 - Vir_g(t)\right)$$

$$E_{i=0,u=0}^{r,a=1} = Age_0 V_r(t)$$

$$E_{i,u=0}^{r,a=2} = \chi PR\_A_2 \sum_{a,u} X_{i,u}^{r,a}(t)$$

Where χ is the migration rate and PR_A_2 the fraction of people aged 25-49 years old in the model

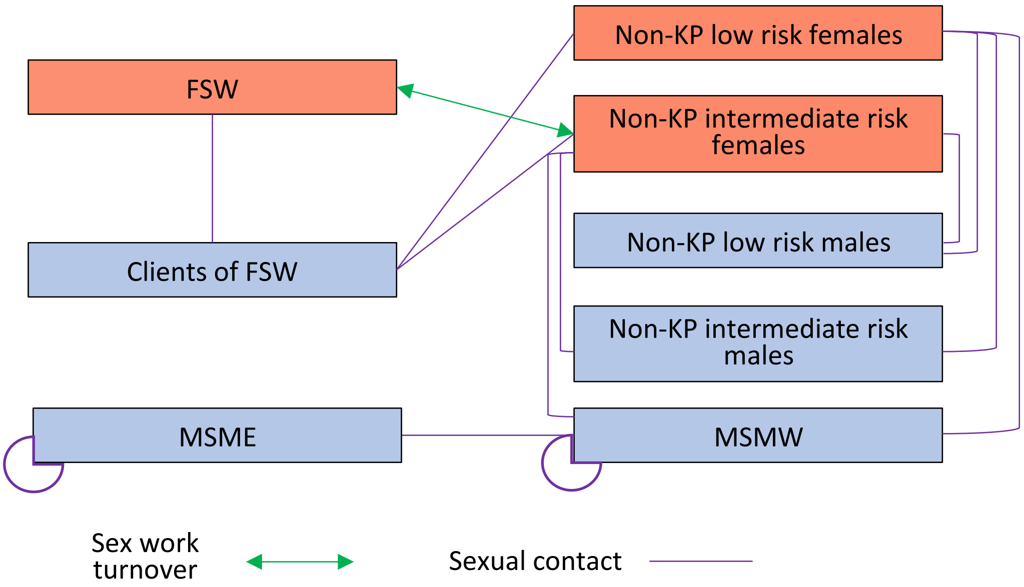

Figure S1a: modelled risk populations and their sexual contacts in the model, adapted from Maheu-Giroux et al. [1]. Female sex workers (FSW) only have sexual contacts with clients but can retire from sex work and move into the compartment of non-KP women with intermediate risk of infection. Clients of FSW have sexual contacts with all women risk groups. Non-KP women (low- and intermediate-risk women) have sexual contacts with all male risk groups, except MSME. The latter are assumed to form partnerships with other male exclusively, whereas MSMW form partnerships with both men and women.

**Table S1a:** Model parameters related to demography and population structure					
**Population**	**Symbol**	**Value/ Prior distribution**			**References**
		**Côte d'Ivoire**	**Mali**	**Senegal**	
**Demography**					
Total population aged 15-59 years in 1970	N_0	2,583,135	3,152,660	2,176,151	[5]
Population growth rate (year^{-1})	ε	3.40%	2.30%	2.85%	[5]
Immigration rate of 25-49 years old (year^{-1})	χ	U(0.0, 0.025)	U(0.0, 0.025)	U(0.0, 0.025)	[5]. Widened range around country estimates. This parameter was fitted to population age distributions in 2020.
Mortality rate (year^{-1})	$\mu_{0:1}$<br>μ_2<br>μ_3	U(0.0201, 0.0207)<br>U(0.0240, 0.0252)<br>U(0.0455, 0.0475)	U(0.0186, 0.0198)<br>U(0.0220, 0.0240)<br>U(0.0425, 0.0440)	U(0.0172, 0.0183)<br>U(0.0205, 0.0218)<br>U(0.0398, 0.0406)	(1/life expectancy at 15, 25, and 50 years)
Proportion of females in population	PR_{Fem}	47.3%	51.1%	51.8%	[5] (for the year 2000)
Age distribution (15-19, 20-24, 25-49, 50-59 years old) in 1970	PR_{A_0}<br>PR_{A_1}<br>PR_{A_2}<br>PR_{A_3}	18.2%<br>14.7%<br>56.5%<br>13.2%	19.7%<br>15.7%<br>51.4%<br>13.2%	19.9%<br>15.8%<br>53.4%<br>10.9%	[5] (for the year 1970)
Rate of ageing in an older risk group or exiting the model at age 60	Age_0<br>Age_1<br>Age_2<br>Age_3	$Age_0 = \frac{1}{5};\ Age_1 = \frac{1}{5};\ Age_2 = \frac{1}{25};\ Age_3 = \frac{1}{10}$			Based on the pre-defined modelled age groups (15-19, 20-24, 25-49, 50-59 years old)
**Population risk-structure**					

Proportion of sexually naïve among 15-19 years old females	$Vir_{g=0}(t)$	Start = 27.4%<br>1994 = 27.4%<br>1999 = 35.9%<br>2004.5 = 34.2%<br>2012 = 35.3%<br>End = 35.3%	Start = 27.4%<br>1995.5 = 27.4%<br>2001 = 36.5%<br>2006 = 45.2%<br>2012.5 = 35.3%<br>2018 = 32.2%<br>End = 32.2%	Start = 64.8%<br>1992.5 = 64.8%<br>1997 = 66.0%<br>2005 = 71.2%<br>2011 = 72.1%<br>2013 = 74.8%<br>2014 = 72.9%<br>2015 = 74.8%<br>2016 = 74.4%<br>2017 = 74.1%<br>2018 = 75.8%<br>2019 = 77.2%<br>End = 77.2%	DHS surveys in Côte d'Ivoire[11-14], Mali[15-19], and Senegal[20-29]. Proportion is assumed to be constant from the last data point.
Proportion of sexually naïve among 15-19 years old males	$Vir_{g=1}(t)$	Start = 44.3%<br>1994 = 44.3%<br>1999 = 44.3%<br>2004.5 = 48.9%<br>2012 = 57.3%<br>End = 57.3%	Start = 63.3%<br>1995.5 = 63.3%<br>2001 = 66.1%<br>2006 = 75.8%<br>2012.5 = 81.3%<br>2018 = 77.5%<br>End = 77.5%	Start = 69.0%<br>1992.5 = 69.0%<br>1997 = 69.0%<br>2005 = 69.0%<br>2011 = 80.9%<br>2013 = 89.0%<br>2014 = 89.0%<br>2015 = 86.5%<br>2016 = 87.8%<br>2017 = 84.6%<br>2018 = 89.3%<br>2019 = 92.4%<br>End = 92.4%	As above
Fraction of FSW among females (assumed constant over time)	PR_{FSW}	U(0.8, 2.1%)	U(0.4, 1.1%)	U(0.5, 0.9%)	Ranges selected using minimum and maximum study point estimates.<br>Côte d'Ivoire: [6,7,30-32]<br>Mali: [7,33]<br>Senegal: [7,34,35]
Fraction of MSM among males (assumed constant over time)	PR_{MSM}	U(0.8, 1.7%)	U(0.2, 0.5%)	U(0.3, 1.2%)	Ranges selected using minimum and maximum study point estimates.<br>Côte d'Ivoire: [36-39]<br>Mali: [7,33]<br>Senegal: [7,40]

Fraction of MSMW among all MSM (assumed constant over time)	PR_{Bi}	U(54.0, 75.5%)	U(53.5, 86.0%)	U(62.0, 85.0%)	Range for Mali selected using minimum and maximum study point estimates. Ranges for Côte d'Ivoire and Senegal were obtained from the pooled estimate of country-specific estimates.<br>Côte d'Ivoire: [39,41-47]<br>Mali: [46,48-50]<br>Senegal: [51-57]
Turnover of sex work (year^{-1})	tur	U(0.067, 0.2)	U(0.067, 0.2)	U(0.067, 0.2)	Assumed as in [1,4]
Fraction of intermediate-risk females (>1 partner/yr) among all non-KP females	PR_{IRF}	U(5, 10%)	U(1, 5%)	U(1, 5%)	Based on estimates from population surveys.<br>Côte d'Ivoire: [11-13,32,58]<br>Mali: [16-18,59]<br>Senegal: [22,23,25-28,60]
Fraction of intermediate-risk males (>2 partner/yr) among all non-KP males	PR_{IRM}	U(5, 10%)	U(1, 5%)	U(1, 5%)	As above
**HIV epidemic seeding**					
Year of epidemic start	HIV_{Start}	U(1975, 1979)	U(1975, 1979)	U(1975, 1979)	Wide range used due to estimates not being available. This parameter was fitted to HIV prevalence data.
HIV prevalence among FSW at epidemic start	$Prev_{FSW}$	U(0.1, 2%)	U(0.1, 2%)	U(0.1, 2%)	As above
HIV prevalence among FSW clients at epidemic start	$Prev_{Cli}$	U(0.1, 1%)	U(0.1, 1%)	U(0.1, 1%)	As above
HIV prevalence among MSM clients at epidemic start	$Prev_{MSM}$	U(0.1, 2%)	U(0.1, 2%)	U(0.1, 2%)	As above

FSW: female sex workers; MSM: men who have sex with men; MSMW: men who have sex with men and women; U: uniform distribution (min, max)

Turnover in sex-work $J_{i,u}^{r,a}$

Female sex-workers (r=2) are assumed to start and cease sex work over their life course (see **Figure S1a** and **Table S1a**).

$J_{i,u}^{r,a}(t) = 0$ if r≠1 and r≠2

$J_{i,u}^{r=1,a}(t) = tur\, X_{i,u}^{r=2,a}(t) - tur'(t)\, X_{i,u}^{r=1,a}(t)$

$J_{i,u}^{r=2,a}(t) = tur'(t)\, X_{i,u}^{r=1,a}(t) - tur\, X_{i,u}^{r=2,a}(t)$

Where tur is the rate at which FSW (r=2) cease forming commercial partnership and transition to the risk group of intermediate-risk females (r=1). This parameter varies across simulations but is fixed over time. Conversely, the parameter $tur'(t)$ represent the rate at which intermediate-risk females initiate sex work and transit into the compartment of FSW. This parameter is calculated at each time step so that the number of women initiating sex work is always equal to the number of women ceasing sex work.

$$tur'(t) = \frac{tur \sum_{r=2,a,i,u} X_{i,u}^{r,a}(t)}{\sum_{r=1,a,i,u} X_{i,u}^{r,a}\,(t)}$$

Non-HIV mortality $Y_{i,u}^{r,a}$

The rates of non-HIV mortality (μ_a) vary by age group a and are sourced from the United Nations Population Division (2019 revision of World Population Prospects) [5].

$Y_{i,u}^{r,a}(t) = -\mu_a X_{i,u}^{r,a}(t)$

Non-HIV mortality rates also apply to the sexually naïve population, with $Y'_r(t) = -\mu_0 V_r(t)$

Population ageing $G_{i,u}^{r,a}$

The rates of ageing of sexually active populations into older age groups (or to exit the model when reaching 60 years old) ($G_{i,u}^{r,a}$), are obtained as the inverse of the number of years covered by the age groups: $Age_0 = \frac{1}{5}$; $Age_1 = \frac{1}{5}$; $Age_2 = \frac{1}{25}$; $Age_3 = \frac{1}{10}$.

The term G'_r correspond to the ageing of sexually naïve populations (all assumed to be 15-19 years old) age into a compartment of 20-24 years of sexually active.

$G'_r(t) = Age_0 V_r(t)$

Whereas,

$G_{i,u}^{r,0}(t) = -Age_0 X_{i,u}^{r,0}(t)$

$G_{i,u}^{r,1}(t) = Age_0 X_{i,u}^{r,0}(t) - Age_1 X_{i,u}^{r,0} + Age_0 V_r(t)$ if (i+u)=0 , and $G_{i,u}^{r,1}(t) = Age_0 X_{i,u}^{r,0}(t) - Age_1 X_{i,u}^{r,0}(t)$ otherwise

$G_{i,u}^{r,2}(t) = Age_1 X_{i,u}^{r,1}(t) - Age_2 X_{i,u}^{r,2}(t)$

$G_{i,u}^{r,3}(t) = Age_2 X_{i,u}^{r,2}(t) - Age_3 X_{i,u}^{r,3}(t)$

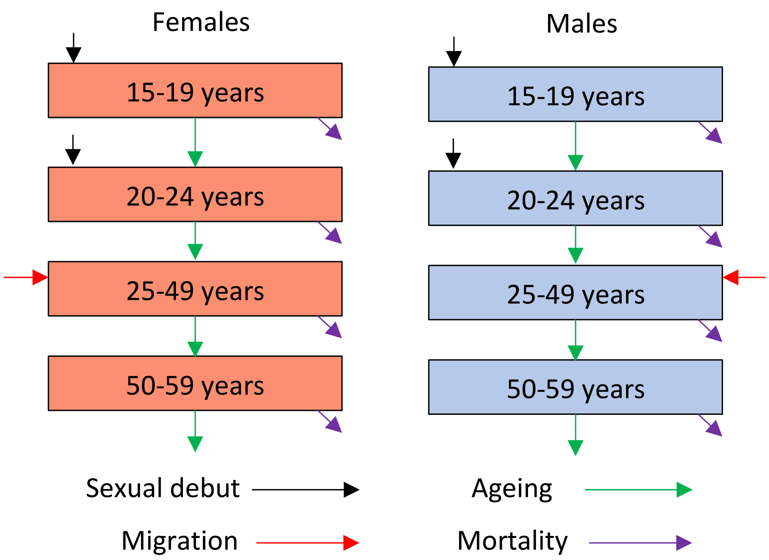

Figure S1b: modelled population age structure, migration, ageing and mortality, adapted from Maheu-Giroux et al. [1]

HIV infection $D_{i,u}^{r,a}$

The force of HIV infection, or rate at which people acquire HIV, vary over time and depend on individual and partner risk factors. We first describe how sexual mixing is represented in the model, then how the force of infection is derived.

Overall sexual mixing

As in [1], sexual mixing was modelled as a function of the gender, age, and risk group of individuals, and informed with data whenever possible. We define $p_{rar'a'}$ as the probability of a sexual partnership between someone of risk group *r* and age *i* with someone of risk group *r'* and age i' , and estimated this quantity using the following equation:

$$p_{rir'i'} = WMW_{rr'}(M_{rr'})\Lambda_{raa'}$$

$$WMW_{rr'} = \begin{pmatrix} & F_{LR} & F_{IR} & F_{FSW} & M_{LR} & M_{IR} & M_{Cli} & M_{MSMW} & M_{MSME} \\ F_{LR} & 0 & 0 & 0 & 1 & 1 & 1 & 1 & 0 \\ F_{IR} & 0 & 0 & 0 & 1 & 1 & 1 & 1 & 0 \\ F_{FSW} & 0 & 0 & 0 & 0 & 0 & 1 & 0 & 0 \\ M_{LR} & 1 & 1 & 0 & 0 & 0 & 0 & 0 & 0 \\ M_{IR} & 1 & 1 & 0 & 0 & 0 & 0 & 0 & 0 \\ M_{Cli} & 1 & 1 & 1 & 0 & 0 & 0 & 0 & 0 \\ M_{MSMW} & 1 & 1 & 0 & 0 & 0 & 0 & 1 & 1 \\ M_{MSME} & 0 & 0 & 0 & 0 & 0 & 0 & 1 & 1 \end{pmatrix}$$

The binary matrix $WMW_{rr'}$ represent the types of partnerships allowed in the model for the risk groups of lower-risk females (F_{LR}), intermediate-risk female (F_{IR}), and female sex worker (F_{FSW}), as well as lower-risk males (M_{LR}), intermediate-risk males (M_{IR}), clients of female sex workers (M_{Cli}), men who have sex with men and women (M_{MSMW}), and men who have sex with men exclusively (M_{MSME}).

As in [1], the sizes of the low- and intermediate-risk groups were based on the number of sexual partners during the last 12 months in the countries DHS surveys (F_{IR} defined as >1 partner per year; M_{IR} defined as >2 partners per year; excluding those that reported selling or buying sex).

Risk-mixing

Sexual mixing by risk group was calculated using sexual behaviour data for low and intermediate-risk individuals. Due to data limitation, mixing by risk group was only available for couples living in the same household who both agreed to be interviewed and reported complete data on their sexual partners in Côte d'Ivoire, and was analysed from the female perspective. A DHS-reported matrix M was expanded to include the other risk groups and their associated parameters: Bi_{Pref} being the fraction of partnerships that are with females for men having sex with men and women (MSMW), Pr_{MSMW} the fraction of MSM also having sex with women (assuming proportional mixing between MSMW and MSME), and Cli_{Mix} the fraction of partnerships that are with FSW for clients of FSW $Cli_{Mix} = \frac{c_5}{c_5+c_{5b}}$, where c_5 and c_{5b} are the clients reported number of commercial partners and non-commercial partners, respectively.

$$M_{rr'} = \begin{pmatrix} & F_{LR} & F_{IR} & F_{FSW} & M_{LR} & M_{IR} & M_{Cli} & M_{MSMW} & M_{MSME} \\ F_{LR} & 0 & 0 & 0 & 0.91 & \frac{0.09*M_{IR}}{M_{IR}+M_{Cli}+M_{MSMW}} & \frac{0.09*M_{Cli}}{M_{IR}+M_{Cli}+M_{MSMW}} & \frac{0.09*M_{MSMW}}{M_{IR}+M_{Cli}+M_{MSMW}} & 0 \\ F_{IR} & 0 & 0 & 0 & 0.88 & \frac{0.12*M_{IR}}{M_{IR}+M_{Cli}+M_{MSMW}} & \frac{0.12*M_{Cli}}{M_{IR}+M_{Cli}+M_{MSMW}} & \frac{0.12*M_{MSMW}}{M_{IR}+M_{Cli}+M_{MSMW}} & 0 \\ F_{FSW} & 0 & 0 & 0 & 0 & 0 & 1 & 0 & 0 \\ M_{LR} & 0.91 & 0.09 & 0 & 0 & 0 & 0 & 0 & 0 \\ M_{IR} & 0.88 & 0.12 & 1 & 0 & 0 & 0 & 0 & 0 \\ M_{Cli} & 0.88(1-Cli_{Mix}) & 0.12(1-Cli_{Mix}) & Cli_{Mix} & 0 & 0 & 0 & 0 & 0 \\ M_{MSMW} & 0.88(Bi_{Pref}) & 0.12(Bi_{Pref}) & 0 & 0 & 0 & 0 & Pr_{MSMW}(1-Bi_{Pref}) & (1-Pr_{MSMW})(1-Bi_{Pref}) \\ M_{MSME} & 0 & 0 & 0 & 0 & 0 & 0 & Pr_{MSMW} & 1-Pr_{MSMW} \end{pmatrix}$$

.

Mixing between risk groups was calculated using data from the 2011-2012 DHS in Côte d'Ivoire. See [1] for further details.

Age-mixing

Mixing patterns by age *(Λ)* were informed using data from the latest DHS surveys available for each country. This survey data reports the age of the most recent sexual partner among the population that was sexually active in the 12 months preceding the survey interview. The age-mixing matrices differ for males and females. As in [1], we assumed that age-mixing between FSW and their clients would correspond to that reported by males in the DHSs. For MSM, it was assumed that age mixing could range between completely assortative and proportional using the tuning parameter MSM_{AgeMix}, which was given a uniform distribution between 0 and 1.

For Côte d'Ivoire:

$$\Lambda_{raa'} = \begin{pmatrix} 0.130 & 0.130 & 0.719 & 0.021 \\ 0.130 & 0.130 & 0.719 & 0.021 \\ 0.003 & 0.003 & 0.753 & 0.241 \\ 0 & 0 & 0.187 & 0.813 \end{pmatrix} \text{ for } r\text{=0,1}$$

$$\Lambda_{raa'} = \begin{pmatrix} 0.483 & 0.483 & 0.035 & 0 \\ 0.483 & 0.483 & 0.035 & 0 \\ 0.194 & 0.194 & 0.609 & 0.004 \\ 0.125 & 0.125 & 0.187 & 0.167 \end{pmatrix} \text{ for } r\text{=2:6}$$

$$\Lambda_{raa'} = (1 - MSM_{AgeMix}) \begin{pmatrix} 1 & 0 & 0 & 0 \\ 0 & 1 & 0 & 0 \\ 0 & 0 & 1 & 0 \\ 0 & 0 & 0 & 1 \end{pmatrix} + MSM_{AgeMix} \begin{pmatrix} 0.182 & 0.147 & 0.565 & 0.106 \\ 0.182 & 0.147 & 0.565 & 0.106 \\ 0.182 & 0.147 & 0.565 & 0.106 \\ 0.182 & 0.147 & 0.565 & 0.106 \end{pmatrix} \text{ for } r\text{=7:8}$$

For Mali:

$$\Lambda_{raa'} = \begin{pmatrix} 0.076 & 0.076 & 0.820 & 0.028 \\ 0.076 & 0.076 & 0.820 & 0.028 \\ 0.001 & 0.001 & 0.674 & 0.324 \\ 0 & 0 & 0.112 & 0.888 \end{pmatrix} \text{ for } r\text{=0,1}$$

$$\Lambda_{raa'} = \begin{pmatrix} 0.496 & 0.496 & 0.008 & 0 \\ 0.496 & 0.496 & 0.008 & 0 \\ 0.175 & 0.175 & 0.645 & 0.004 \\ 0.024 & 0.024 & 0.876 & 0.077 \end{pmatrix} \text{ for } r\text{=2:6}$$

$$\Lambda_{raa'} = \left(1 - MSM_{AgeMix}\right)\begin{pmatrix} 1 & 0 & 0 & 0 \\ 0 & 1 & 0 & 0 \\ 0 & 0 & 1 & 0 \\ 0 & 0 & 0 & 1 \end{pmatrix} + MSM_{AgeMix}\begin{pmatrix} 0.197 & 0.157 & 0.514 & 0.132 \\ 0.197 & 0.157 & 0.514 & 0.132 \\ 0.197 & 0.157 & 0.514 & 0.132 \\ 0.197 & 0.157 & 0.514 & 0.132 \end{pmatrix} \text{ for } r\text{=7:8}$$

For Senegal:

$$\Lambda_{raa'} = \begin{pmatrix} 0.043 & 0.043 & 0.886 & 0.028 \\ 0.043 & 0.043 & 0.886 & 0.028 \\ 0.001 & 0.001 & 0.638 & 0.361 \\ 0 & 0 & 0.088 & 0.912 \end{pmatrix} \text{ for } r\text{=0,1}$$

$$\Lambda_{raa'} = \begin{pmatrix} 0.494 & 0.494 & 0.012 & 0 \\ 0.494 & 0.494 & 0.012 & 0 \\ 0.182 & 0.182 & 0.632 & 0.003 \\ 0.026 & 0.026 & 0.865 & 0.103 \end{pmatrix} \text{ for } r\text{=2:6}$$

$$\Lambda_{raa'} = \left(1 - MSM_{AgeMix}\right)\begin{pmatrix} 1 & 0 & 0 & 0 \\ 0 & 1 & 0 & 0 \\ 0 & 0 & 1 & 0 \\ 0 & 0 & 0 & 1 \end{pmatrix} + MSM_{AgeMix}\begin{pmatrix} 0.199 & 0.158 & 0.534 & 0.109 \\ 0.199 & 0.158 & 0.534 & 0.109 \\ 0.199 & 0.158 & 0.534 & 0.109 \\ 0.199 & 0.158 & 0.534 & 0.109 \end{pmatrix} \text{ for } r\text{=7:8}$$

As in [1], probabilities of sexual partnerships are calculated separately for each partnership type *(rar'a')*. Imbalances between sexual partnerships demand and offer of the groups are likely (e.g. males typically reporting higher number of partners than females). The balance between supply of and demand for sexual partnership was obtained using a modified partner change rate *($c^*_{rar'a'}$)* using the method described by Garnett and Anderson [61], and below:

$$\Delta_{rar'a'} = \frac{c_{r'a'} p_{r'a'ra} N_{r'a'}}{c_{ra} p_{rar'a'} N_{ra}}$$

$$c^*_{rar'a'} = c_{ra} \Delta^{\eta_k}_{rar'a'}$$

$$c^*_{r'a'ra} = c_{r'a'} \Delta^{-(1-\eta_k)}_{rar'a'}$$

Here, the parameter $\Delta_{rar'a'}$ measures the degree of imbalance between supply and demand for sexual partnerships of type *rar'a'*, while η_k is the balance parameter which determines the degree to which partners alter their demand/offer of sexual partnerships. We assumed that clients of FSW would drive demand, whereas the balance parameter was assumed to be equal to 0.5 for MSM.

HIV force of infection

As in [1], we defined the force of infection (i.e. annual probability of HIV transmission) from an individual of risk group *r'* and age class *a'* to an individual of risk group *r* and age class *a* [62]. This probability partly depends on a "base" per-sex-act probability of transmission β which has different

values depending on the sex of both partners, with 1) female-to-male transmission probabilities ($= \beta_{fm}$) being lower than 2) male-to-female ($= \beta_{fm}RR_{\beta mf}$) and 3) male-to-male ($= \beta_{fm}RR_{\beta mm}$) transmission probabilities. Per-act transmission probabilities are further altered by a term of cofactors $Cof^{iu}_{gg'a'}$ (see next page and **Table S1c**).

The matrix $\lambda_{rar'a'}(t)$ reflects the transmission probabilities for each partnership combination of category *r/r'* and age *a/a'*, where the susceptible partner is the one indexed by ra. The terms g' and g refers to the susceptible partner and infectious partner sex (=0 if female, =1 if male)

$$\lambda_{rar'a'}(t) = c^*_{rar'a'}p_{rar'a'}\left[\sum_{i,t}\left(\left(\frac{I^{iu}_{r'a'}(t)}{\sum_{i,t}I^{iu}_{r'a'}(t)+S_{r'a'}(t)}\right)\left(1-\left(\left(1-\beta Cof^{iu}_{gg'a'}(t)\right)^{\alpha_{rar'a'}(1-\nu_{ra}(t))}\left(1-\beta Cof^{iu}_{gg'a'}(t)(1-\varsigma)\right)^{\alpha_{rar'a'}(\nu_{ra}(t))}\right)\right)\right)\right]$$

Where $Cof^{iu}_{gg'a'}(t)$ represent a combination of cofactors for HIV acquisition (related to the susceptible individual gender g' and age a') and transmissions (related to the infectious individual disease stage *i* and treatment status *u*). Here, the model accounts for the elevated risk of acquisition among young women ($RR_{\beta YF}$) (compared to older women) [63,64], the elevated risk of HIV transmission of individuals in the acute stage of HIV infection ($RR_{\beta Acute}$) [65]. We also assume that an increasing fraction of PLHIV on ART ($VLS_g(t)$) have a suppressed viral load and can't transmit HIV [66]. Estimates of this fraction are available over time and by sex from UNAIDS[67]. PLHIV on ART that don't have a suppressed viral load $\left(1-(VLS_g(t)\ VLSPtl)\right)$ are assumed to transmit HIV at the same rate as those not on ART.

The cofactor term $Cof^{iu}_{gg'a'}(t)$ is described below under three specific cases:

1) when both susceptible and infected partners don't have any specific risk factor for HIV acquisition/transmission (if $(g' = 1\ or\ a' > 1)\ and\ i > 1\ and\ u \neq 5$):

1.1.: $Cof^{iu}_{gg'a'}(t) = 1$

2) when the susceptible individual is not a young woman (i.e. if $g' = 1\ or\ a' > 1$) and the infected partner has a modified risk of HIV transmission because he/she is in the acute stage of infection or in on ART (i.e. if $i = 1\ or\ u = 5$):

2.1.: $Cof^{iu}_{gg'a'}(t) = RR_{\beta Acute}\ if\ i = 1\ and\ u \neq 5$

2.2.: $Cof^{iu}_{gg'a'}(t) = \left(1-\left(VLS_g(t)\ VLSPtl\right)\right)\ if\ i \neq 1\ and\ u = 5$

2.3.: $Cof^{iu}_{gg'a'}(t) = RR_{\beta Acute}\left(1-\left((VLS_g(t)\ VLSPtl\right)\right)\ if\ i = 1\ and\ u = 5$

3) when the susceptible individual is a young woman (i.e. if $g' = 0 \; and \; a' \leq 1$) and the infected partner has a modified risk of HIV transmission because it is in the acute stage of infection or in on ART (if $i = 1 \; or \; u = 5$):

2.1.: $Cof_{gg'a'}^{iu}(t) = RR_{\beta Acute} RR_{\beta YF} \; if \; i = 1 \; and \; u \neq 5$

2.2.: $Cof_{gg'a'}^{iu}(t) = \left(1 - \left(VLS_g(t) \; VLSPtl\right)\right) RR_{\beta YF} \; if \; i \neq 1 \; and \; u = 5$

2.3.: $Cof_{gg'a'}^{iu}(t) = RR_{\beta Acute} \left(1 - \left(VLS_g(t) \; VLSPtl\right)\right) RR_{\beta YF} \; if \; i = 1 \; and \; u = 5$

**Table S1b:** Model parameters related to sexual behaviours					
**Parameter**	**Symbol**	**Prior distribution**			**References**
		**Côte d'Ivoire**	**Mali**	**Senegal**	
**Number of sexual partners of sexually active risk groups**					
Lower-risk females	c_0	U(0.8, 0.9)	U(0.79, 0.84)	U(0.63, 0.66)	Range selected as minimum and maximum values across DHS surveys in Côte d'Ivoire [11-14], Mali[15-19], and Senegal[20-29]
Intermediate-risk females	c_1	U(2.4, 9.4)	U(2.0, 2.8)	U(2.0, 2.2)	As above
Lower-risk males	c_3	U(1.0, 1.2)	U(0.86, 0.92)	U(0.61, 0.73)	As above
Intermediate-risk males	c_4	U(4.7, 6.8)	U(4.2, 8.2)	U(3.2, 7.0)	As above
FSW	c_2	U(216.0, 360.0)	U(200.0, 1007.0)	U(182.0, 273.0)	Range selected as minimum and maximum estimates from studies among FSW in Côte d'Ivoire [68-71], Mali [72], and Senegal [73-76]
Clients of FSW with FSW	c_5	U(23.0, 37.0)	U(23.0, 37.0)	U(23.0, 42.0)	Surveys in Côte d'Ivoire [77] and Senegal (personal communication of estimated from an unpublished IBBS client survey[78]). No data for Mali (used Côte d'Ivoire data)
Clients of FSW with non-KP females	c_{5b}	U(1.0, 6.8)	U(1.0, 6.8)	U(2.5, 4.5)	Surveys in Côte d'Ivoire[68-71] and Senegal[78]. No data for Mali (used Côte d'Ivoire data)
MSMW	c_6	U(1.0, 10.0)	U(1.0, 10.0)	U(1.0, 10.0)	Conservative assumption
MSME	c_7	U(1.0, 10.0)	U(1.0, 10.0)	U(1.0, 10.0)	Conservative assumption
**Number of sex acts per partner-year**					
Lower-risk partners (r=0,3)	$\alpha_{rir'i'}$	U(40.0, 48.0)	U(40.0, 48.0)	U(40.0, 48.0)	[11]
Intermediate-risk partners (r=1,4)	$\alpha_{rir'i'}$	U(33.0, 66.0)	U(33.0, 66.0)	U(33.0, 66.0)	[11]
Clients-FSW partners (r=3,5)	$\alpha_{rir'i'}$	U(1.0, 4.0)	U(1.0, 4.0)	U(1.0, 4.0)	[11]
MSM partners (r=6,7)	$\alpha_{rir'i'}$	U(33.0, 66.0)	U(33.0, 66.0)	U(26.4, 39.6)	Data from MSM survey in Senegal [79]. Conservative assumption for Côte d'Ivoire and Mali
Increase in numbers of sex acts of MSM from 2016 compared to before 2007	$RR\alpha_{MSM2016}$	No increase assumed	No increase assumed	U(1.5, 2.0)	Assuming a linear trend between 2007 [53] and 2016 [56]

Sexual balance parameter as per Garnett et al. 1994 [61]	η	U(0, 1)	U(0, 1)	U(0, 1)	Assumption
Proportion of partnerships that are with females for MSMW	Bi_{Pref}	U(32.0, 44.3%)	U(30.0, 45.0%)	U(34.7, 42.0%)	Surveys among MSM in Côte d'Ivoire [37] and Senegal [79]. Range for Mali expanded from Côte d'Ivoire and Senegal estimates (as no Mali data available)
Tuning parameter between assortative and proportional mixing by age among MSM	MSM_{AgeMix}	U(0, 1)	U(0, 1)	U(0, 1)	Assumption

FSW: female sex workers; MSM: men who have sex with men; MSMW: men who have sex with men and women; MSME: men who have sex with men exclusively; U: uniform distribution (min, max)

**Table S1c:** Model parameters related to HIV infection and transmission (prior ranges assumed similar across countries)			
**Parameter**	**Symbol**	**Prior distribution**	**References**
**Natural history progression (PLHIV not on ART)**			
Average duration of acute infection (years)	$1/\gamma_0$	U(0.11, 0.18)	Uncertainty around the point estimate of 1.7 month from [65].
Average time from seroconversion to 350 CD4 cells/µL	$1/\gamma_0 + 1/\gamma_1 + 1/\gamma_2$	U(2.2, 4.6)	Ranges selected from the 50%UI of estimates of [80], as in [1]
Average time from 350 CD4 to 200 CD4 cells/µL	$1/\gamma_3$	U(3.9, 5.0)	As above
Average time from 200 CD4 cells/µL to death	$1/\gamma_4$	U(1.9, 3.9)	As above
**HIV transmission**			
Female-to-male transmission probability per sex act	β_{fm}	U(0.001, 0.017)	Range around study point estimates from [81,82]
RR of HIV transmission from male to female compared to from female to male	$RR_{\beta mf}$	U(1, 3)	Range around study point estimates from [81,83]
RR of HIV transmission between males compared to from female to male	$RR_{\beta mm}$	U(2, 6)	Range around study point estimates from [83,84]
RR of HIV acquisition of females aged <25 years compared to females aged ≥25 years	$RR_{\beta YF}$	U(1.25, 2.5)	Range around study point estimates from [63,64]
Excess hazard-months of HIV transmission attributable to the acute stage	$EHM_{\beta Acute}$	U(4.2, 16.8)	Conservative range over the point estimate of [65]. This parameter is used to calculate the RR of HIV transmission during acute HIV infection $RR_{\beta Acute}$ using the formula $RR_{\beta Acute} = \left(\frac{EHM_{\beta Acute}}{12/\gamma_0}\right) + 1$
RR of HIV transmission among when a condom is used during a sex act (vs during a condomless sex act)	ς	U(0.75, 0.942)	Assumption based on [85]
**ART and viral suppression**			
ART initiation rate in the AIDS stage (<200 CD4 cells/µL)	ρ_4	U(0.5, 4.0)	Conservative assumption taken from [1]
Slope cofactor shaping linear relation between CD4 stages and ART initiation	ϖ	U(0, 1.0)	As above
RR of ART initiation among diagnosed PLHIV in 2000 compared to 2020	$RR_{\rho 2000}$	U(0, 1.0)	As above
RR of ART initiation among KP compared to non-KP	$RR_{\rho KP}$	U(0.2, 5.0)	As above
RR survival extension cofactor by HIV diagnosis/treatment status	$RR\omega_u$	U(2.2, 6.3) if *u*=5, and 0 otherwise (only PLHIV on ART experience a reduced HIV mortality)	Range selected from minimum and maximum effects across ALPHA study sites in [9]
ART drop-out rate prior to 2015	φ	U(0.15, 0.27)	As in [1], the lower bound selected as median of national estimates for Cote d'Ivoire over 2008-2013[86-92], whereas the upper bound is taken from a collaboration of 11 cohorts of HIV-infected adult patients in Western Africa [93].

RR of ART drop-out for FSW and MSM (vs non-KP or clients)	$RR_{\varphi KP}$	U(1.25, 1.75)	Uncertainty around estimate from [94] (FSW data)
RR of ART drop-out after 2015 compared to before 2015	$RR_{\varphi 2015p}$	U(0.75, 1.00)	Assumption

FSW: female sex workers; MSM: men who have sex with men; MSMW: men who have sex with men and women; MSME: men who have sex with men exclusively; RR: relative risk; U: uniform distribution (min, max)

**Table S1d:** Model parameters related to condom use					
**Parameter**	**Symbol**	**Prior distribution**			**References**
		**Côte d'Ivoire**	**Mali**	**Senegal**	
**During sex acts between non-KP groups**					
Among 15-24 years old	$Condom_{0:1}(t)$	Start = (0-5%)<br>$YrCond_{NonKP}$= (3-5%)<br>1995 = (9.9-47.2%)<br>1999 = (11.5-55.7%)<br>2006 = (20.9-53.5%)<br>2012 = (20.7-59.5%)<br>End = (20.7-59.5%)	Start = (0-1%)<br>$YrCond_{NonKP}$= (1-2%)<br>2001 = (2.8-20.5%)<br>2006 = (20.6-30.5%)<br>2012 = (4.1-26.4%)<br>2018 = (2.5-20.7%)<br>End = (2.5-20.7%)	Start = (0-1%)<br>$YrCond_{NonKP}$= (1-2%)<br>2005 = (1.2-5.6%)<br>2011 = (0.0-1.8%)<br>2014 = (2.8-4.8%)<br>2016 = (1.7-3.8%)<br>2018 = (1.3-2.4%)<br>End = (1.3-2.4%)	[95,96] For estimates in the early 1980's. Range from DHS surveys in Côte d'Ivoire [11-14], Mali[15-19], and Senegal[20-29], using levels reported by females as minimum, and reported by males as maximum
Among 25-49 years old	$Condom_{2}(t)$	Start = (0-2.5%)<br>$YrCond_{NonKP}$= (1-2.5%)<br>1995 = (2.5-21.7%)<br>1999 = (3.1-21.8%)<br>2006 = (4.7-23.8%)<br>2012 = (7.3-24.2%)<br>End = (7.3-24.2%)	Start = (0-1%)<br>$YrCond_{NonKP}$= (0.5-1%)<br>2001 = (0.1-4.1%)<br>2006 = (1.0-4.4%)<br>2012 = (1.4-4.5%)<br>2018 = (0.9-5.4%)<br>End = (0.9-5.4%)	Start = (0-1%)<br>$YrCond_{NonKP}$= (0.5-1%)<br>2005 = (1.2-4.6%)<br>2011 = (1.6-3.3%)<br>2014 = (1.5-2.0%)<br>2016 = (1.8-2.8%)<br>2018 = (0.5-1.5%)<br>End = (0.5-1.5%)	As above
Among 50-59 years old	$Condom_{3}(t)$	Start = (0-2%)<br>$YrCond_{NonKP}$= (0.2-2%)<br>1995 = (0.2-9.7%)<br>1999 = (0.6-9.7%)<br>2006 = (0.6-10.0%)<br>2012 = (1.8-11.5%)<br>End = (1.8-11.5%)	Start = (0-1%)<br>$YrCond_{NonKP}$= (0.5-1%)<br>2001 = (0.1-4.1%)<br>2006 = (1.0-4.4%)<br>2012 = (1.4-4.5%)<br>2018 = (0.9-5.4%)<br>End = (0.9-5.4%)	Start = (0-1%)<br>$YrCond_{NonKP}$= (0.5-1%)<br>2005 = (1.2-4.6%)<br>2011 = (1.6-3.3%)<br>2014 = (1.5-2.0%)<br>2016 = (1.8-2.8%)<br>2018 = (0.5-1.5%)<br>End = (0.5-1.5%)	As above
**During sex acts of KP**					

All FSW with clients	$CondomSW(t)$	Start = (0-5%)<br>$YrCond_{SW}$= (5-15%)<br>1991 = (57-68%)<br>1993 = (74-81%)<br>1995 = (73-88%)<br>1997 = (88-93%)<br>1998 = (88-98%)<br>2002 = (91-99%)<br>2007 = (90-99%)<br>2012 = (90-95%)<br>2014 = (85-93%)<br>End = (85-93%)	Start = (0-5%)<br>$YrCond_{SW}$= (5-15%)<br>1997 = (74-83%)<br>2000 = (90-98%)<br>2003 = (94-98%)<br>2009 = (94-98%)<br>2018 = (95-98%)<br>End = (95-98%)	Start = (0-5%)<br>$YrCond_{SW}$= (5-15%)<br>1990 = (79-89%)<br>2006 = (93-99%)<br>2010 = (90-98%)<br>2015 = (95-99%)<br>2019 = (88-96%)<br>End = (88-96%)	Surveys among FSW in Côte d'Ivoire[68,69,71,97], Mali[72,98-103] and Senegal[73-76,104]
All MSM	$CondomMSM(t)$	Start = (0-0%)<br>$YrCond_{MSM}$= (5-15%)<br>2004 = (35-50%)<br>2012 = (57-69%)<br>2015 = (63-81%)<br>2017 = (68-82%)<br>End = (68-82%)	Start = (0-0%)<br>$YrCond_{MSM}$= (5-15%)<br>2014 = (70-82%)<br>2018 = (70-82%)<br>End = (70-82%)	Start = (0-0%)<br>$YrCond_{MSM}$= (5-15%)<br>2005 = (76-77%)<br>2014 = (73-76%)<br>2016 = (70-84%)<br>End = (70-84%)	Surveys among MSM in Côte d'Ivoire[37,41,51], Mali[33,105] and Senegal[53,56,78,106]. Estimates in Côte d'Ivoire and Senegal similar to levels of condom use reported by CohMSM participants (~75%) [46].
**Year of increase in condom use in the 1980's**					
Non-KP groups (r=0,1,3,4)	$YrCond_{NonKP}$	U(1981, 1990)	U(1981, 1990)	U(1981, 1990)	Assumption
FSW with clients (r=2,5)	$YrCond_{SW}$	U(1981, 1990)	U(1981, 1990)	U(1981, 1990)	Assumption
MSM (r=6,7)	$YrCond_{MSM}$	U(1981, 1990)	U(1981, 1990)	U(1981, 1990)	Assumption
**Scaling factors for the proportion of sex acts protected by condoms**					
Among non-KP aged 15-24 years	$CondPtl_{0:1}$	U(0, 1)	U(0, 1)	U(0, 1)	Assumption
Among non-KP aged 25-49 years	$CondPtl_2$	U(0, 1)	U(0, 1)	U(0, 1)	Assumption
Among non-KP aged 50-59 years	$CondPtl_3$	U(0, 1)	U(0, 1)	U(0, 1)	Assumption
All FSW with clients	$CondSWPtl$	U(0, 1)	U(0, 1)	U(0, 1)	Assumption
All MSM	$CondMSMPtl$	U(0, 1)	U(0, 1)	U(0, 1)	Assumption
RR actual condom use during sex work (vs reported)	RR_{CondSW}	U(0.7,1.0)	U(0.7,1.0)	U(0.7,1.0)	Conservative assumption based on studies among clients of FSW, or using biomarkers, pooling booth surveys or list randomisation[107-109]

FSW: female sex workers; MSM: men who have sex with men; MSMW: men who have sex with men and women; MSME: men who have sex with men exclusively; RR: relative risk; U: uniform distribution (min, max)

HIV disease progression and mortality $Q_{i,u}^{r,a}$

At each time step, newly infected PLHIV progress through different stages of infections according to their CD4 cell counts, expressed by the superscript "*i*", (with *i*=0 corresponding to HIV-uninfected people, **Figure S1c**). The first stage of HIV infection (*i*=1) corresponds to acute infection, the second (*i*=2) to CD4>500 cells/µL, the third (*i*=3) for CD4 between 350 and 500 cells/µL, the fourth (*i*=4) for CD4 between 200 and 350 cells/µL, and the last one (*i*=5) for CD4 <200 cells/µL, the latter stage being associated with HIV-related mortality. Transitions rates between infection stages (γ_i) were sourced from the literature and shown in **Table S1c**. As in [1], the reduced HIV-mortality among PLHIV on ART was obtained by reducing the transition rates γ_3 and γ_4 by a factor $RR\omega_u$ (informed by mortality data) which is > 1 only when *u*=5.

For HIV-uninfected people: $Q_{i=0,u,s}^{r,a}(t) = 0$

For PLHIV:

$Q_{i=1,u}^{r,a}(t) = -\gamma_0 X_{i=1,u}^{r,a}(t)$, if in the acute infection stage

$Q_{i=2,u}^{r,a}(t) = \gamma_0 X_{i=1,u}^{r,a}(t) - \gamma_1 X_{i=2,u}^{r,a}(t)$, if CD4>500 cells/µL

$Q_{i=3,u}^{r,a}(t) = \gamma_1 X_{i=2,u}^{r,a}(t) - \gamma_2 X_{i=3,u}^{r,a}(t)$, if CD4 between 350 and 500 cells/µL

$Q_{i=4,u}^{r,a}(t) = \gamma_2 X_{i=3,u}^{r,a}(t) - (\frac{\gamma_3}{RR\omega_u}) X_{i=4,u}^{r,a}(t)$, if CD4 between 200 and 350 cells/µL

$Q_{i=5,u}^{r,a}(t) = (\frac{\gamma_3}{RR\omega_u}) X_{i=4,u}^{r,a}(t) - (\frac{\gamma_4}{RR\omega_u}) X_{i=5,u}^{r,a}(t)$, if CD4<200 cells/µL

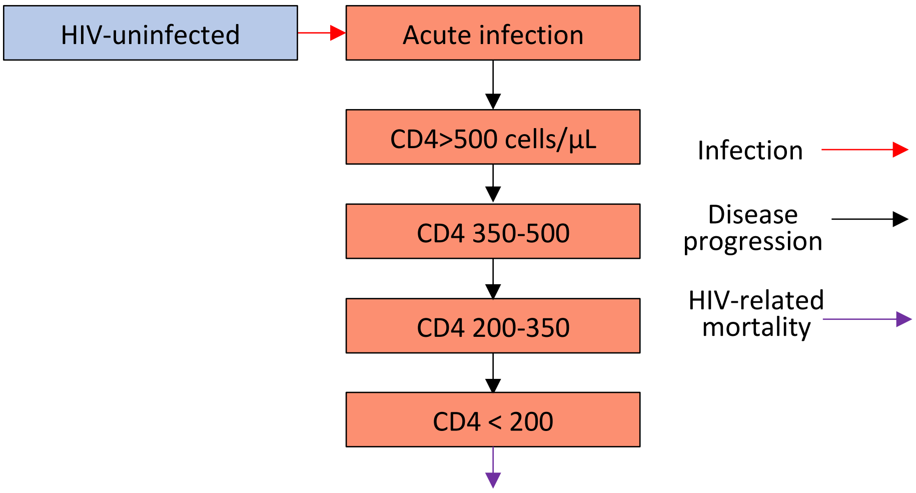

Figure S1c: modelled HIV infection stages among people not on ART.

HIV conventional testing and diagnosis $T_{i,u}^{r,a}$

The rates of conventional HIV testing and diagnosis in the modelled populations vary over time and are informed by the trends in proportions of people having tested for HIV in the last year (parameter $\tau^{g,a}(t)$) (see **Table S2e**). This data was available by sex and age group from countries successive DHS surveys. As in[1], in order to replicate the trends in testing rates and maintain temporal consistency despite uncertainties in exact levels of testing, the parameter $TestPtl$ is used to represent the percentile of each data uncertainty range, and is sampled between 0 and 1 within each simulation. The variable $Year_\tau$ is the time where people start testing for HIV in each country, and is sampled in each simulation.

$T_{i,u,s}^{r,a}(t) = 0$ if time $\leq Year_\tau$

When time > $Year_\tau$, population flows will depend on population HIV and diagnosis/treatment status.

Among HIV-uninfected populations (i=0):

$$T_{i,u=0}^{r,a}(t) = -X_{i,u=0}^{r,a}\ (t)\ \tau^{g,a}(t)\ RRTest_{i,u=0}^{r}(t)\ K_g$$

$T_{i,u=1}^{r,a}(t) = 0$ (conventional HIV tests by this population are accounted for, but do not correspond to a flow into another population group)

Among PLHIV (i >0):

$$T_{i,u=0}^{r,a}(t) = -X_{i,u=0,s}^{r,a}\ (t)\ \tau^{g,a}(t)\ RRTest_{i,u=0}^{r}(t)\ K_g$$

$$T_{i,u=1}^{r,a}(t) = -X_{i,u=1,s=0}^{r,a}\ (t)\ \tau^{g,a}(t)\ RRTest_{i,u=1}^{r}(t)\ K_g$$

$$T_{i,u=3,s=0}^{r,a}(t) = \Big(\big(X_{i,u=0,s}^{r,a}\ (t)\ \tau^{g,a}(t)\ RRTest_{i,u=0}^{r}(t)\big) + \big(X_{i,u=1,s=0}^{r,a}\ (t)\ \tau^{g,a}(t)\ RRTest_{i,u=1}^{r}(t)\big)\Big)\ K_g$$

$$T_{i,u=4,s}^{r,a}(t) = X_{i,u=2,s=1}^{r,a}\ (t)\ \tau^{g,a}(t)\ RRTest_{i,u=2}^{r}(t)\ K_g$$

$T_{i,u\geq 5,s}^{r,a}(t) = 0$ (conventional HIV tests by PLHIV on ART or that have dropped-out from ART are accounted for, but do not correspond to a flow into another population group)

Where the parameter $RRTest_{i,u}^{r}(t)$ is a product of cofactors (relative risks) defined using wide uncertainty ranges which aimed at reproducing empirical heterogeneities in HIV testing coverage by risk group, HIV status and HIV testing history status (see **Table S1e**):

- The parameter $RR_{\tau AIDS}$ reflects the increase in HIV conventional testing among PLHIV in the AIDS stage of infection (compared to PLHIV not in the AIDS stage).
- The parameters $RR_{\tau FSWStart}$ and $RR_{\tau FSW2020}$ represent the elevated HIV conventional testing rates among FSW compared to non-FSW females at the time points $Year_\tau$ and 2020,

with the RR for a specific time being calculated assuming a linear trend between $RR_{\tau FSWStart}$ and $RR_{\tau FSW2020}$ over the period [$Year_{\tau}$ – 2020]. A similar assumption is made for the HIV testing rates of MSM (compared to non-MSM males), using the parameters $RR_{\tau MSMStart}$ and $RR_{\tau MSM2020}$.

- Heterogeneities in HIV conventional testing rates among PLHIV vs HIV-uninfected populations are represented using one parameter for KPs ($RR_{\tau PLHIV\_KP}$) and one for non-KPs ($RR_{\tau PLHIV\_NKP}$), and similarly for populations never having tested for HIV (vs ever testing) (using the parameters $RR_{\tau NTest\_KP}$ and $RR_{\tau NTest\_NKP}$).
- Possible heterogeneities in HIV conventional testing among diagnosed PLHIV (compared to undiagnosed PLHIV) are captured using the parameter $RR_{\tau Diagn}$.

An overall K_g parameter is used as an overall fudge factor to fit the model to history of HIV testing (by risk group, and HIV status when available) as well as number of conventional HIV tests done in the countries.

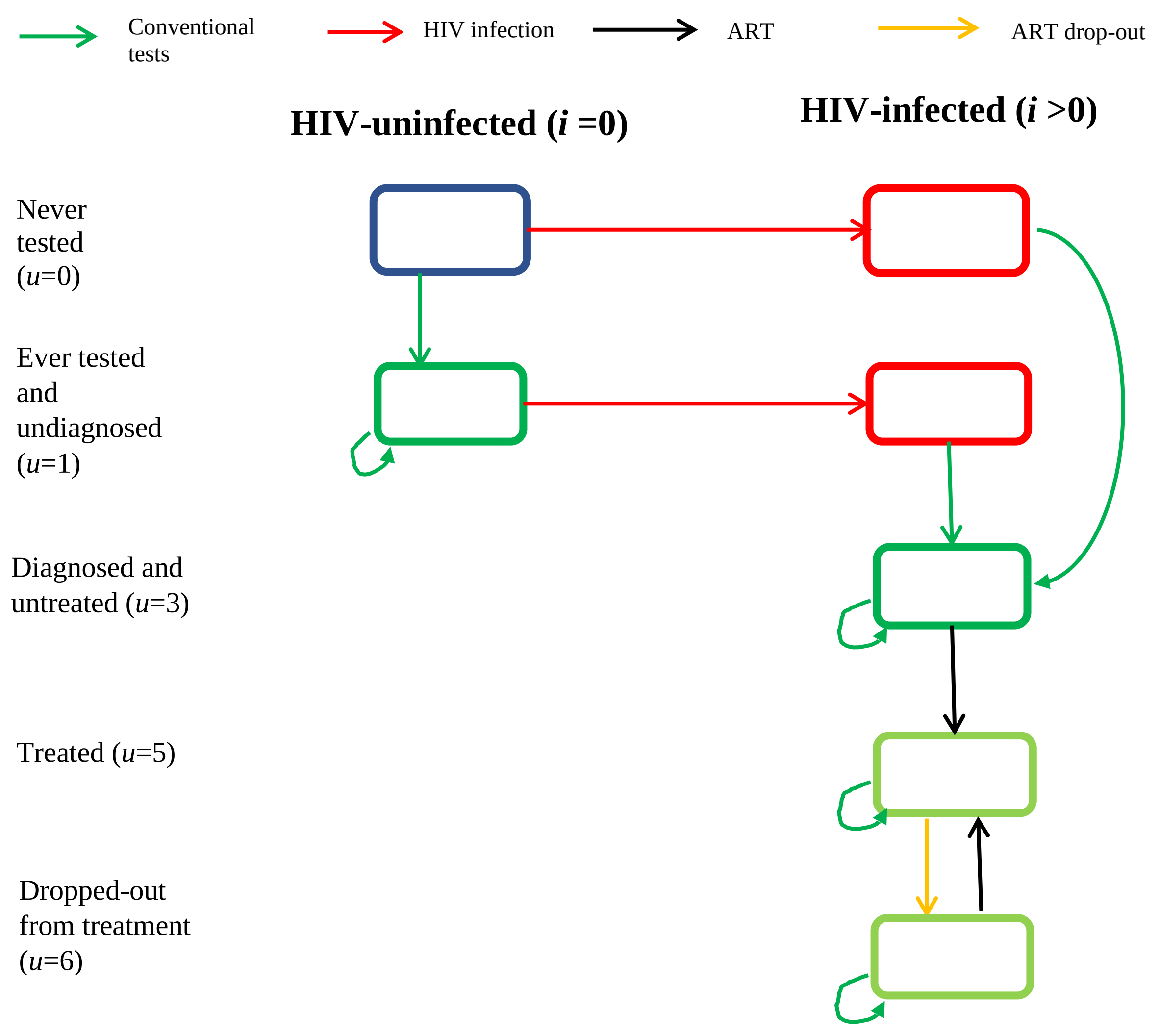

Figure S1d: modelled stages of HIV testing, diagnosis and treatment.

**Table S1e:** Model parameters related to HIV testing and treatment					
**Parameter**	**Symbol**	**Prior distribution**			**References**
		**Côte d'Ivoire**	**Mali**	**Senegal**	
**HIV conventional testing probabilities (last 12 months) $\boldsymbol{\tau^{g,a}(t)}$**					
Start year of HIV testing	$Year_\tau$	U(1996, 1999)	U(1996, 1999)	U(1996, 1999)	Conservative assumption based on World Bank report on HIV response in Western Africa [110].
Non-FSW females aged 15-24 years	$\tau^{0,0:1}(t)$	Start = (0, 0%)<br>$Year_\tau$ = (0, 0%)<br>2005 = (1, 6%)<br>2011 = (7, 26%)<br>2017 = (8, 33%)<br>End = (8, 33%)	Start = (0, 0%)<br>$Year_\tau$ = (0, 0%)<br>2006 = (2, 7%)<br>2013 = (3, 12%)<br>2018 = (4, 15%)<br>End = (4, 15%)	Start = (0, 0%)<br>$Year_\tau$ = (0, 0%)<br>2005 = (0, 2%)<br>2011 = (5, 25%)<br>2014 = (7, 28%)<br>End = (7, 28%)	DHS surveys in Côte d'Ivoire [11-14], Mali[15-19], and Senegal[20-23,29]
Non-FSW females aged 25-49 years	$\tau^{0,2}(t)$	Start = (0, 0%)<br>$Year_\tau$ = (0, 0%)<br>2005 = (2, 9%)<br>2011 = (7, 26%)<br>2017 = (11, 45%)<br>End = (11, 45%)	Start = (0, 0%)<br>$Year_\tau$ = (0, 0%)<br>2006 = (1, 5%)<br>2013 = (3, 12%)<br>2018 = (5, 18%)<br>End = (5, 18%)	Start = (0, 0%)<br>$Year_\tau$ = (0, 0%)<br>2005 = (0, 1%)<br>2011 = (7, 28%)<br>2014 = (8, 33%)<br>End = (8, 33%)	As above
Non-FSW females aged 50-59 years	$\tau^{0,3}(t)$	Start = (0, 0%)<br>$Year_\tau$ = (0, 0%)<br>2005 = (2, 9%)<br>2011 = (7, 26%)<br>2017 = (11, 45%)<br>End = (11, 45%)	Start = (0, 0%)<br>$Year_\tau$ = (0, 0%)<br>2006 = (1, 3%)<br>2013 = (2, 4%)<br>2018 = (3, 12%)<br>End = (3, 12%)	Start = (0, 0%)<br>$Year_\tau$ = (0, 0%)<br>2005 = (0, 2%)<br>2011 = (4, 17%)<br>2014 = (6, 25%)<br>End = (6, 25%)	As above
Non-MSM males aged 15-24 years	$\tau^{1,0:1}(t)$	Start = (0, 0%)<br>$Year_\tau$ = (0, 0%)<br>2005 = (1, 4%)<br>2011 = (4, 15%)<br>2017 = (3, 11%)<br>End = (3, 11%)	Start = (0, 0%)<br>$Year_\tau$ = (0, 0%)<br>2006 = (1, 4%)<br>2013 = (2, 7%)<br>2018 = (1, 4%)<br>End = (1, 4%)	Start = (0, 0%)<br>$Year_\tau$ = (0, 0%)<br>2005 = (0, 2%)<br>2011 = (3, 15%)<br>2014 = (3, 15%)<br>End = (3, 15%)	As above
Non-MSM males aged 25-49 years	$\tau^{1,2}(t)$	Start = (0, 0%)<br>$Year_\tau$ = (0, 0%)<br>2005 = (2, 8%)<br>2011 = (5, 22%)<br>2017 = (7, 28%)<br>End = (7, 28%)	Start = (0, 0%)<br>$Year_\tau$ = (0, 0%)<br>2006 = (2, 10%)<br>2013 = (4, 15%)<br>2018 = (3, 12%)<br>End = (3, 12%)	Start = (0, 0%)<br>$Year_\tau$ = (0, 0%)<br>2005 = (1, 4%)<br>2011 = (5, 20%)<br>2014 = (5, 20%)<br>End = (5, 20%)	As above

Non-MSM males aged 50-59 years	$\tau^{1,3}(t)$	Start = (0, 0%)<br>$Year_\tau$ = (0, 0%)<br>2005 = (1, 3%)<br>2011 = (5, 22%)<br>2017 = (4, 16%)<br>End = (4, 16%)	Start = (0, 0%)<br>$Year_\tau$ = (0, 0%)<br>2006 = (2, 10%)<br>2013 = (4, 15%)<br>2018 = (3, 12%)<br>End = (3, 12%)	Start = (0, 0%)<br>$Year_\tau$ = (0, 0%)<br>2005 = (1, 4%)<br>2011 = (4, 17%)<br>2014 = (3, 15%)<br>End = (3, 15%)	As above
Scaling factor for HIV testing among non-FSW and non-MSM	$TestPtl$	U(0, 1)	U(0, 1)	U(0, 1)	Assumed
Overall HIV testing scaling factor (by gender)	K_g	U(0.2, 5)	U(0.2, 5)	U(0.2, 5)	Conservative assumption. This parameter is fitted to HIV diagnosis and treatment data, including programmatic data on the number of HIV tests done in each country over time.
**Relative change in conventional testing rate among populations**					
RR HIV testing among PLHIV in AIDS stage (vs PLHIV not in the AIDS stage)	$RR_{\tau AIDS}$	U(1, 8)	U(1, 8)	U(1, 8)	Conservative assumption similar to [1]
RR HIV testing among FSW (vs non-KP females) at $Year_\tau$	$RR_{\tau FSWStart}$	U(1, 10)	U(1, 10)	U(1, 10)	Conservative assumption based on the higher reported history of testing among FSW compared to non-FSW women
RR HIV testing among FSW (vs non-KP females) from 2020	$RR_{\tau FSW2020}$	U(1, 10)	U(1, 10)	U(1, 10)	As above
RR HIV testing among MSM (vs non-MSM males) at $Year_\tau$	$RR_{\tau MSMStart}$	U(1, 10)	U(1, 10)	U(1, 10)	Conservative assumption based on the higher reported history of testing among MSM compared to non-MSM men
RR HIV testing among MSM (vs non-MSM males) from 2020	$RR_{\tau MSM2020}$	U(1, 10)	U(1, 10)	U(1, 10)	As above
RR HIV testing among KP PLHIV (vs HIV-uninfected KP)	$RR_{\tau PLHIV\_KP}$	U(0.2, 5)	U(0.2, 5)	U(0.2, 5)	Assumed
RR HIV testing among non-KP PLHIV (vs HIV-uninfected non-KP)	$RR_{\tau PLHIV\_NKP}$	U(0.2, 5)	U(0.2, 5)	U(0.2, 5)	Assumed
RR HIV testing among KP never having tested (vs KP ever tested)	$RR_{\tau NTest\_KP}$	U(0.2, 5)	U(0.2, 5)	U(0.2, 5)	Assumed
RR HIV testing among non-KP never having tested (vs non-KP ever tested)	$RR_{\tau NTest\_NKP}$	U(0.2, 5)	U(0.2, 5)	U(0.2, 5)	Assumed

RR HIV testing among diagnosed PLHIV (vs undiagnosed)	$RR_{\tau Diagn}$	U(0.2, 3)	U(0.2, 3)	U(0.2, 3)	Assumed
**Viral suppression among PLHIV**					
Fraction of female PLHIV on ART that have a suppressed viral load	$VLS_{g=0}(t)$	Start = (40-60%)<br>2000 = (40-60%)<br>2018 = (70-89%)<br>2020 = (73-93%)<br>2030 = (85-95%)<br>End = (85-95%)	Start = (40-60%)<br>2000 = (40-60%)<br>2020 = (63-83%)<br>2030 = (85-95%)<br>End = (85-95%)	Start = (40-60%)<br>2000 = (40-60%)<br>2016 = (73-91%)<br>2020 = (77-97%)<br>2030 = (85-97%)<br>End = (85-97%)	From[67] for 2016 and 2020, assumptions otherwise
Fraction of male PLHIV on ART that have a suppressed viral load	$VLS_{g=1}(t)$	Start = (40-60%)<br>2000 = (40-60%)<br>2018 = (69-91%)<br>2020 = (75-97%)<br>2030 = (85-97%)<br>End = (85-97%)	Start = (40-60%)<br>2000 = (40-60%)<br>2020 = (65-87%)<br>2030 = (85-95%)<br>End = (85-97%)	Start = (40-60%)<br>2000 = (40-60%)<br>2016 = (66-83%)<br>2020 = (76-96%)<br>2030 = (85-96%)<br>End = (85-96%)	As above
Scaling factor for viral suppression among PLHIV on ART	$VLSPtl$	U(0, 1)	U(0, 1)	U(0, 1)	Assumption

FSW: female sex workers; MSM: men who have sex with men; MSMW: men who have sex with men and women; MSME: men who have sex with men exclusively; RR: Relative risk; U: uniform distribution (min, max)

HIV treatment initiation and drop-out $U_{i,u}^{r,a}$

Our model reused the approach from[1,4] to represent ART initiation, which accounts for changes in ART eligibility, while reflecting the fact that CD4 cell counts may have been widely available in the region[91]. Our model assumes that individuals could only initiate ART after being diagnosed, hence no treatment was given until the period 1980-$Year_\tau$, with $Year_\tau$ being sampled over 1996-1999. PLHIV diagnosed through a confirmed reactive self-test initiate ART at a rate ρ_{ST}, informed by ATLAS survey data (see **Figure S1d**).

$U_{i,u}^{r,a}(t) = 0$ if u ≤ 2

$$U_{i,u=3}^{r,a}(t) = -UStart_i \, RRUStart_r(t) \, X_{i,u=3,s}^{r,a}(t)$$

$$U_{i,u=4}^{r,a}(t) = -\rho_{ST} \, X_{i,u=4,s}^{r,a}(t)$$

$$U_{i,u=5}^{r,a}(t) = UStart_i \, RRUStart_r(t) \, X_{i,u=3}^{r,a}(t) + \left(\left(UStart_i \, RRUStart_r(t)\right) - \left(UStop \, RRUStop(t)\right)\right) X_{i,u=6}^{r,a}(t)$$

$$U_{i,u=6}^{r,a}(t) = UStop \, RRUStop(t) \, X_{i,u=6}^{r,a}(t)$$

The parameter $UStart_i$ is the "base" rate of ART initiation and depend on PLHIV HIV stage of infection, whereas $UStop$ is the "base" rate of ceasing ART. As in [1,4], we assumed that PLHIV with a diagnosed infection and a CD4 count <200 cells/μL were more likely to show clinical symptoms and initiate ART. A linear relation was assumed between CD4 count stage and initiation rate using parameter ϖ. This parameter was given a uniform prior over [0-1] so that PLHIV with lower CD4 cell counts always had higher initiation rates. ART initiation rates were first sampled among PLHIV with a CD4 count <200 cells/μL ($UStart_4 = \rho_4$), and the rates among the other infection stages were calculated using the formula described in [1], where $m = ((0 - \rho_4)/9.25)\,\varpi$, $UStart_3 = \rho_3 = 3m - \rho_4$, $UStart_2 = \rho_2 = 6m - \rho_4$, and $UStart_1 = \rho_1 = 9m - \rho_4$.

The parameter $RRUStart_r(t)$ is a product of cofactors for initiating ART (see **Table S1c**): the parameter $RR_{\rho 2000}$ is the relative risk of ART initiation among PLHIV with a diagnosed infection at the time $Year_\tau$ compared to 2020, and we assumed that ART initiation rates would linearly increase between $Year_\tau$ and 2020, then stay constant at the 2020 levels after this period. Our model allowed the ART initiation rates to differ between KP and non-KP, using a wide prior range for a parameter $RR_{\rho KP}$.

Similarly, the parameter $RRUStop_r(t)$ is a product of cofactors for ceasing ART: our model assumed that ART drop-out rates could slightly decrease over time and be different between FSW and MSM compared to non-KP and FSW clients.

A fraction of PLHIV on ART have a suppressed viral load and are assumed not to be able to transmit HIV. This fraction increases over time and vary by sex ($VLS_g(t)$), using estimates from UNAIDS[67]. The scaling factor $VLSPtl$ sampled between 0 and 1 was used to reflect uncertainties in estimates as well as overall time trends.

Model fitting overview

Each model was fitted under a Bayesian framework in three steps. In the first step, a Latin hypercube of model parameters was used to simulate 50M simulations from prior distributions of the parameters describe above. In the second step, we only retained the simulations which agreed with all the widened confidence intervals of the fitting outcomes (i.e., prior constraints) described in **Tables S2a-c** (between 579 and 1550 simulations were retained across models). In the third step, the 100 fitted simulations with the highest overall likelihood (calculated by summing the simulation likelihood across all outcomes using their original sample size, except on HIV incidence rate, number of conventional tests and fraction of positive tests, for which there were no sample size) were identified for each country. The resulting posterior parameter sets were used to simulate all our model scenarios.

Model fitting data

Fitting data (Côte d'Ivoire)

Table S2a: List of demographic, epidemiological, and intervention outcomes used for model fitting in Côte d'Ivoire

Population or age group	Year	Point estimate (sample size used for simulation likelihood calculation)	Original 95%CI	Prior constraint	Reference
**Population size**					
Total number of 15-59 years-old	1970<br>1980<br>1990<br>2000<br>2010<br>2020	2.58 million<br>4.05 million<br>6.02 million<br>8.51 million<br>10.64 million<br>14.19 million	N.A.	Initial value for 1970 and direct calibration using growth rate between 1970 and 2020 estimates	From [5]
**Age distribution among 15–59-year-old females**					

	1970	15-24 years: 33.5%<br>25-49 years: 55.5%<br>50-59 years: 10.9%	N.A.	Used for comparison	From [5]. This data was only used for comparison because we used UNPD estimates for the year 1970 combining female and males as model input (Table S1a), and fitted our model to sex-specific UNPD estimates for 2020.
	1980	15-24 years: 37.1%<br>25-49 years: 52.7%<br>50-59 years: 10.4%	N.A.	Used for comparison	As above
	1990	15-24 years: 38.1%<br>25-49 years: 51.7%<br>50-59 years: 10.2%	N.A.	Used for comparison	As above
	2000	15-24 years: 40.0%<br>25-49 years: 51.1%<br>50-59 years: 9.0%	N.A.	Used for comparison	As above
	2010	15-24 years: 39.6%<br>25-49 years: 51.0%<br>50-59 years: 9.3%	N.A.	Used for comparison	As above
	2020	15-24 years: 38.7%<br>25-49 years: 52.3%<br>50-59 years: 9.0%	N.A.	35.2-42.6%<br>47.5-57.5%<br>6.9-11.7%	Fitted from [5]
**Age distribution among 15–59-year-old males**					
	1970	15-24 years: 32,2%<br>25-49 years: 57.5%<br>50-59 years: 10.3%	N.A.	Used for comparison	From [5]. This data was only used for comparison because we used UNPD estimates for the year 1970 combining female and males as model input (Table S1a), and fitted our model to sex-specific UNPD estimates for 2020.
	1980	15-24 years: 33.4%<br>25-49 years: 56.1%<br>50-59 years: 10.5%	N.A.	Used for comparison	As above
	1990	15-24 years: 33.9%<br>25-49 years: 54.9%<br>50-59 years: 11.3%	N.A.	Used for comparison	As above
	2000	15-24 years: 36.6%<br>25-49 years: 52.7%<br>50-59 years: 10.7%	N.A.	Used for comparison	As above
	2010	15-24 years: 37.6%<br>25-49 years: 51.7%<br>50-59 years: 10.7%	N.A.	Used for comparison	As above
	2020	15-24 years: 38.0%<br>25-49 years: 52.1%<br>50-59 years: 9.9%	N.A.	34.5-41.8%<br>47.4-57.3%<br>7.6-12.9%	Fitted from [5]
**HIV prevalence among all adult females (except female sex workers)**					
15-24	1989	2.5% (n=713)	(0.9-4.4%)	0.1-18.0%	[111]
15-24	2005	2.4% (n=1054)	(1.6-3.0%)	1.0-10.0%	[12]
15-24	2012	2.2% (n=1314)	(1.5-3.0%)	1.0-10.0%	[11]
15-24	2017	0.9% (n=3186)	(0.5-1.4%)	0.2-6.0%	[32]
15-24	2018	0.4%	(0.0-0.8%)	Only used for comparison	[112]. This study was identified post model fitting but had a low sample size (5 women were living with HIV).

25-49	1989	2.5% (n=1020)	(1.0-4.3%)	0.8-20.0%	[111] (estimate for the 25-54 year old age group, as estimate for the 25-49 year old age groups was not available)
25-49	2005	9.9% (n=1221)	(8.4-12.0%)	4.0-18.0%	[12]
25-49	2012	6.3% (n=1546)	(5.2-8.0%)	3.0-15.0%	[11]
25-49	2017	5.5% (n=4840)	(4.5-6.8%)	3.0-8.0%	[32]
50-59	1989	1.9% (n=192)	(0.4-4.4%)	0.1-10.0%	[111] (estimate for the 50-64 year old age group, as estimate for the 50-59 year old age groups was not available)
50-59	2005	10.2% (n=139)	(6.2-16.0%)	4.0-20.0%	[12] (prevalence for the 45-49 years old age group)
50-59	2012	9.5% (n=146)	(5.7-15.0%)	4.0-20.0%	[11] (prevalence for the 45-49 Years old age group)
50-59	2017	8.2% (n=723)	(5.0-13.1%)	4.0-20.0%	[32]
**HIV prevalence among all adult males**					
15-24	1989	2.4% (n=664)	(0.8-4.3%)	0.5-15.0%	[111]
15-24	2005	0.3% (n=1069)	(0.1-1.0%)	0.1-4.0%	[12]
15-24	2012	0.3% (n=1090)	(0.1-1.0%)	0.1-4.0%	[11]
15-24	2017	0.3% (n=2750)	(0.1-0.9%)	0.1-4.0%	[32]
15-24	2018	0.3%	(0.0-0.6%)	Only used for comparison	[112]. This study was identified post model fitting but had a low sample size (2 men were living with HIV).
25-49	1989	7.7% (n=858)	(5.5-10.0%)	2.0-40.0%	[111] (estimate for the 25-54 year old age group, as estimate for the 25-49 year old age groups was not available)
25-49	2005	4.8% (n=907)	(3.6-6.0%)	2.0-15.0%	[12]
25-49	2012	4.3% (n=1427)	(3.3-5.0%)	2.0-15.0%	[11]
25-49	2017	2.1% (n=4923)	(1.4-2.9%)	1.0-4.0%	[32]
50-59	1989	1.7% (n=283)	(0.4-3.9%)	1.0-25.0%	[111]
50-59	2005	4.9% (n=151)	(2.4-10.0%)	2.0-20.0%	[12]
50-59	2012	8.7% (n=184)	(5.4-14.0%)	4.0-30.0%	[11]
50-59	2017	3.6% (n=907)	(1.7-7.4%)	2.0-15.0%	[32]
**HIV prevalence among all female sex workers**					
15-59	1986	36.9% (n=101)	(27.6-46.7%)	10.0-85.0%	[113]
15-59	1987	36.9% (n=116)	(30.1-48.2%)	10.0-85.0%	[113]
15-59	1989	47.6% (n=120)	(38.4-56.7%)	10.0-85.0%	[113]
15-59	1990	68.4% (n=72)	(57.0-78.6%)	10.0-85.0%	[113]
15-59	1994	67.0% (n=607)	(63.2-70.8%)	10.0-85.0%	[68]
15-59	1995	54.0% (n=832)	(50.5-57.4%)	10.0-85.0%	[68]
15-59	1996	52.0% (n=916)	(48.7-55.2%)	10.0-85.0%	[68]
15-59	1997	52.0% (n=876)	(48.7-55.4%)	10.0-85.0%	[68]
15-59	1998	32.0% (n=876)	(28.9-35.3%)	10.0-65.0%	From [114]. As sample size was not available but data was from the same clinic as [68], we assumed the same sample size as the 1998 estimates.
15-59	1999	32.0% (n=876)	(28.7-35.5%)	10.0-65.0%	As above
15-59	2000	28.0% (n=876)	(24.8-31.4%)	10.0-55.0%	As above
15-59	2001	31.0% (n=876)	(27.7-34.4%)	10.0-55.0%	As above
15-59	2002	27.0% (n=876)	(23.8-30.3%)	10.0-55.0%	As above
15-59	2003	33.0% (n=876)	(28.0-38.0%)	10.0-55.0%	From [115]. As sample size was not available but data was from the same clinic as [68], we assumed the same sample size as the 1998 estimates.

15-59	2004	27.0% (n=876)	(22.4-31.9%)	10.0-55.0%	[115]
15-59	2005	18.0% (n=876)	(14.1-22.4%)	8.0-40.0%	[115]
15-59	2006	19.0% (n=876)	(15.1-23.6%)	8.0-40.0%	[115]
15-59	2007	21.0% (n=876)	(16.9-25.7%)	8.0-40.0%	[115]
15-59	2007	22.9% (n=446)	(13.7-35.6%)	8.0-40.0%	[116]
15-59	2008	19.0% (n=876)	(15.1-23.6%)	8.0-40.0%	[115]
15-59	2009	20.0% (n=876)	(15.9-22.5%)	8.0-40.0%	[115]
15-59	2009	11.2% (n=446)	(6.4-18.9%)	8.0-40.0%	[116]
15-59	2010	21.0% (n=569)	(10.0-35.0%)	8.0-40.0%	[115]
15-59	2014	11.0% (n=500)	(8.3-14.3%)	5.0-25.0%	[97]. Sample size not available and assumes n=500.
15-59	2016	11.4% (n=466)	(8.8-14.8%)	5.0-25.0%	[69]
15-59	2020	4.9% (n=1177)	(3.8-6.3%)	2.0-15.0%	[117]
**HIV prevalence among clients of sex workers**					
15-59	1999	13.4% (n=423)	(10.5-17.0%)	5.0-30.0%	[77]
**HIV Prevalence among MSM**					
All 15-59	2015	11.2% (n=1301)	(9.6-13.1%)	5.0-25.0%	[43]
All 15-59	2016	19.6% (n=500)	N.A.	10.0-40.0%	[118]. Sample size not available and assumed n=500.
All 15-59	2017	12.3% (n=365)	(9.3-16.1%)	5.0-25.0%	[119]
All 15-59	2020	6.4% (n=1301)	(5.2-7.5%)	4.0-20.0%	[47]
All 15-24	2012	12.5% (n=355)	(6.8-18.2%)	3.0-35.0%	[41]
All 15-24	2015	11.4% (n=329)	(6.6-19.0%)	3.0-25.0%	[43]
All 15-24	2020	5.1% (n=633)	(3.4-6.8%)	2.0-15.0%	[47]. Sample size not available and assumed half of the study total sample size (n=1265)
All 25-49	2012	24.8% (n=246)	(16.7-34.9%)	10.0-60.0%	[41]
All 25-49	2015	16.3% (n=643)	(7.5-32.0%)	5.0-40.0%	[43]
All 25-49	2020	11.2% (n=633)	(7.3-14.9%)	3.0-22.0%	[47]. Sample size not available and assumed half of the study total sample size (n=1265)
All MSMW	2012	12.3% (n=327)	(9.2-16.3%)	5.0-25.0%	[41]
All MSMW`	2020	6.2% (n=633)	(4.1-8.3%)	3.0-15.0%	[47]. Sample size not available and assumed half of the study total sample size (n=1265)
All MSME	2012	25.7% (n=264)	(20.8-31.3%)	10.0-45.0%	[41]
All MSME	2020	6.2% (n=633)	(4.0-8.2%)	3.0-15.0%	[47]. Sample size not available and assumed half of the study total sample size (n=1265)
**HIV incidence rate (per 100 susceptible-year)**					
15-59	2005	0.161	(0.069-0.304)	0.03-0.75	[120]. No sample size available.
15-59	2010	0.152	(0.065-0.286)	0.03-0.75	As above
15-59	2017	0.129	(0.055-0.243)	0.05-0.24	As above
**Number of new HIV infections**					
15-59	2005	18100	(7900-34000)	3000-65000	As above
15-59	2010	22000	(9700-41000)	4000-80000	As above
15-59	2017	26000	(11400-49000)	11400-49000	As above
**Number of HIV-related deaths**					
15-59	2005	48000	(29000-72000)	1000-100000	As above
15-59	2010	29000	(17500-45000)	5000-70000	As above
15-59	2017	21700	(12700-32000)	12700-32000	As above
**Fraction of all females ever tested for HIV**					
15-49	2000	7.2% (n=11475)	(6.7-7.7%)	1.0-25.0%	[121]
15-49	2005	10.9% (n=5177)	(8.2-14.5%)	5.0-35.0%	[12]
15-49	2011	35.4% (n=9937)	(33.1-37.8%)	20.0-50.0%	[11]
15-49	2016	56.0% (n=11780)	(53.9-58.0%)	35.0-75.0%	[122]

15-24	2009	25.3% (n=2325)	(23.6-27.1,)	10.0-50.0%	[123]
**Fraction of all females not living with HIV ever tested for HIV**					
15-49	2005	10.0% (n=4274)	(7.2-13.7%)	4.0-25.0%	[12]
15-49	2011	34.5% (n=4385)	(31.8-37.2%)	15.0-55.0%	[11]
15-49	2017	56.3% (n=5000)	(54.3-58.2%)	35.0-75.0%	[32]. Sample size not available and assumed n=5000.
**Fraction of all females living with HIV ever tested for HIV**					
15-49	2005	13.6% (n=255)	(7.9-22.5%)	5.0-40.0%	[12]
15-49	2011	42.0% (n=208)	(34.2-50.2%)	20.0-70.0%	[11]
15-49	2017	74.7% (n=300)	(67.5-82.0%)	50.0-95.0%	[32]. Sample size not available.
**Fraction of all males ever tested for HIV**					
15-49	2005	7.9% (n=4500)	(6.2-9.9%)	3.0-25.0%	[12]
15-49	2011	23.1% (n=4677)	(20.7-25.7%)	10.0-40.0%	[11]
15-49	2016	34.6% (n=5405)	(32.1-37.1%)	20.0-55.0%	[122]
15-24 males	2009	18.1% (n=2537)	(16.7-19.6%)	5.0-40.0%	[123]
**Fraction of all males not living with HIV ever tested for HIV**					
15-49	2005	7.4% (n=3791)	(6.2-9.9%)	3.0-20.0%	[12]
15-49	2011	23.0% (n=3851)	(20.7-25.7%)	10.0-40.0%	[11]
15-49	2017	32.2% (n=5000)	(30.1-34.4%)	20.0-45.0%	[32]. Sample size not available and assumed n=5000.
**Fraction of all males living with HIV ever tested for HIV**					
15-49	2005	23.7% (n=97)	(10.9-44.1%)	8.0-50.0%	[12]
15-49	2011	39.0% (n=107)	(28.4-50.7%)	25.0-65.0%	[11]
15-49	2017	53.4% (n=300)	(39.1-67.6%)	30.0-75.0%	[32]. Sample size not available and assumed n=300.
**Fraction of FSW ever tested for HIV**					
15-59	2007	54.0% (n=2461)	(52.0-56.0%)	30.0-85.0%	[124]
15-59	2020	82.0% (n=1177)	(79.0-83.0%)	65.0-92.0%	[117]
15-24	2014	75.3% (n=178)	(68.5-81.0%)	50.0-95.0%	[97]
25-49	2014	85.7% (n=245)	(81.1-89.3%)	65.0-99.0%	[97]
**Fraction of MSM ever tested for HIV**					
15-59	2011	62.6% (n=601)	(56.5-68.2%)	40.0-95.0%	[41]
15-59	2015	92.4% (n=105)	(85.7-96.1%)	70.0-99.0%	[125]
15-59	2020	70.0% (n=1265)	(67.0-72.0%)	60.0-90.0%	[47]
**Fraction of all females living with HIV which are diagnosed**					
15-59	2000	4.2% (n=500)	(3.5-5.0%)	1.0-15.0%	[126]. No sample size available but was assumed n=500.
15-59	2005	14.7% (n=500)	(12.2-17.6%)	5.0-30.0%	As above
15-59	2010	42.0% (n=500)	(35.0-50.3%)	20.0-60.0%	As above
15-59	2015	69.0% (n=500)	(58.3-84.0%)	50.0-95.0%	As above
15-59	2020	84.7% (n=500)	(70.5-99.0%)	70.0-99.0%	As above
**Fraction of all males living with HIV which are diagnosed**					
15-59	2000	3.2% (n=500)	(2.7-3.8%)	1.0-15.0%	As above
15-59	2005	10.9% (n=500)	(9.0-13.1%)	5.0-25.0%	As above
15-59	2010	29.1% (n=500)	(24.3-34.9%)	15.0-50.0%	As above
15-59	2015	49.9% (n=500)	(41.6-59.9%)	30.0-70.0%	As above
15-59	2020	68.1% (n=500)	(56.7-81.7%)	50.0-90.0%	As above
**Fraction of all FSW living with HIV with a diagnosed infection**					
15-59	2014	26.7% (n=45)	(15.9-41.0%)	5.0-70.0%	[97]
15-59	2020	81.0% (n=57)	(69.0-89.0%)	50.0-95.0%	[117]
**Fraction of all MSM living with HIV with a diagnosed infection**					
15-59	2011	15.9% (n=113)	(10.3-23.8%)	30.0-50.0%	[41]
15-59	2015	37.0% (n=146)	(29.6-45.1%)	15.0-65.0%	[37]
15-59	2017	26.7% (n=45)	(16.0-41.0%)	10.0-60.0%	[45]
15-59	2020	32.7% (n=98)	(34.1-42.4%)	15.0-60.0%	[47]
**Fraction of all females living with HIV with a treated infection**					
15-59	2015	44.0%	(40.0-50.0%)	25.0-60.0%	[67]. No sample size available.
15-59	2020	83.0%	(74.0-94.0%)	69.0-99.0%	As above

**Fraction of all males living with HIV with a treated infection**					
15-59	2015	29.0%	(25.0-34.0%)	15.0-50.0%	As above
15-59	2020	61.0%	(55.0-71.0%)	49.0-78.0%	As above
**Fraction of all FSW living with HIV with a treated infection**					
15-59	2012	45.6% (n=163)	(42.2-49.1%)	20.0-70.0%	[115]
15-59	2020	65.0% (n=32)	(52.0-76.0%)	52.0-76.0%	[117]
**Fraction of PLHIV with a suppressed viral load**					
15-49 females	2017	38.4% (n=240)	(29.2-47.7%)	20.0-55.0%	[32]
15-49 males	2017	20.1% (n=97)	(11.9-28.3%)	5.0-40.0%	[32]
15-49 MSM	2017	19.4% (n=36)	(9.8-35.2%)	10.0-45.0%	[45]
**Number of conventional HIV tests done by females each year**					
15-59	2015	1,601,691	N.A.	(800,846-3,203,382)	Programmatic data reported by countries to UNAIDS's Shiny90[126]. No sample size available.
15-59	2016	1,826,826	N.A.	(913,413-3,653,652)	As above
15-59	2017	1,631,236	N.A.	(815,718-3,262,672)	As above
15-59	2018	1,809,731	N.A.	(904,866-3,619,462)	As above
**Number of conventional HIV tests done by males each year**					
15-59	2015	492,691	N.A.	(246,346-985,382)	As above
15-59	2016	553,680	N.A.	(276,840-1,107,360)	As above
15-59	2017	437,692	N.A.	(218,846-975,384)	As above
15-59	2018	902,838	N.A.	(451,419-1,805,676)	As above
**Fraction of conventional HIV tests done by females which are positive**					
15-59	2015	3.2%	N.A.	(1.6-6.5%)	As above
15-59	2016	2.6%	N.A.	(1.3-5.2%)	As above
15-59	2017	2.2%	N.A.	(1.1-4.4%)	As above
15-59	2018	1.8%	N.A.	(0.9-3.5%)	As above
**Fraction of conventional HIV tests done by males which are positive**					
15-59	2015	3.6%	N.A.	(1.8-7.1%)	As above
15-59	2016	3.0%	N.A.	(1.5-6.0%)	As above
15-59	2017	3.0%	N.A.	(1.5-6.0%)	As above
15-59	2018	3.5%	N.A.	(1.8-7.0%)	As above

MSMW: men who have sex with men as well as female partners; MSME: men who have sex with men exclusively.
N.A.: Not available

Fitting data (Mali)

Table S2b: List of demographic, epidemiological, and intervention outcomes used for model fitting in Mali					
**Population or age group**	**Year**	**Point estimate (sample size used for simulation likelihood calculation)**	**Original 95%CI**	**Prior constraint**	**Reference**
**Population size**					
Total number of 15-59 years-old	1970<br>1980<br>1990<br>2000<br>2010<br>2020	3.15 million<br>3.57 million<br>4.00 million<br>5.30 million<br>7.27 million<br>9.95 million	N.A.	Initial value for 1970 and direct calibration using growth rate between 1970 and 2020 estimates	From [5]
**Age distribution among 15–59-year-old females**					
	1970	15-24 years: 35.0%<br>25-49 years: 51.6%<br>50-59 years: 13.4%	N.A.	Used for comparison	From [5]. This data was only used for comparison because we used UNPD estimates for the year 1970 combining female and males as model input (Table S1a), and fitted our model to sex-specific UNPD estimates for 2020.
	1980	15-24 years: 36.3%<br>25-49 years: 51.5%<br>50-59 years: 12.2%	N.A.	Used for comparison	As above
	1990	15-24 years: 39.0%<br>25-49 years: 49.8%<br>50-59 years: 11.2%	N.A.	Used for comparison	As above
	2000	15-24 years: 40.5%<br>25-49 years: 49.3%<br>50-59 years: 10.2%	N.A.	Used for comparison	As above
	2010	15-24 years: 39.0%<br>25-49 years: 51.7%<br>50-59 years: 9.3%	N.A.	Used for comparison	As above
	2020	15-24 years: 39.9%<br>25-49 years: 51.1%<br>50-59 years: 9.0%	N.A.	30.7-51.9%<br>39.3-66.4%<br>6.0-13.5%	Fitted from [5]
**Age distribution among 15–59-year-old males**					
	1970	15-24 years: 35.8%<br>25-49 years: 51.2%<br>50-59 years: 13.0%	N.A.	Used for comparison	From [5]. This data was only used for comparison because we used UNPD estimates for the year 1970 combining female and males as model input (Table S1a), and fitted our model to sex-specific UNPD estimates for 2020.
	1980	15-24 years: 38.0%<br>25-49 years: 50.2%<br>50-59 years: 11.8%	N.A.	Used for comparison	As above
	1990	15-24 years: 42.0%<br>25-49 years: 47.9%<br>50-59 years: 10.1%	N.A.	Used for comparison	As above

	2000	15-24 years: 43.3%<br>25-49 years: 48.1%<br>50-59 years: 8.6%	N.A.	Used for comparison	As above
	2010	15-24 years: 40.5%<br>25-49 years: 51.5%<br>50-59 years: 8.0%	N.A.	Used for comparison	As above
	2020	15-24 years: 41.1%<br>25-49 years: 50.9%<br>50-59 years: 8.0%	N.A.	31.6-53.4%<br>39.2-66.2%<br>5.3-12.0%	Fitted from [5]
**HIV prevalence among all adult females (except female sex workers)**					
15-24	2001	1.3% (n=1546)	(0.7-2.0%)	0.1-5.0%	[18]
15-24	2006	0.9% (n=1850)	(0.4-1.5%)	0.1-4.0%	[17]
15-24	2013	1.1% (n=1713)	(0.6-1.7%)	0.3-2.3%	[16]
25-49	2001	2.6% (n=2289)	(1.0-4.3%)	0.5-10.0%	[18]
25-49	2006	1.7% (n=2677)	(1.0-2.4%)	0.2-7.0%	[17]
25-49	2013	1.5% (n=3093)	(1.0-1.9%)	0.4-2.9%	[16]
**HIV prevalence among all adult males**					
15-24	2001	0.3% (n=994)	(0.0-0.7%)	0.0-3.0%	[18]
15-24	2006	0.5% (n=1492)	(0.0-0.9%)	0.0-4.0%	[17]
15-24	2013	0.3% (n=1210)	(0.0-0.7%)	0.0-1.0%	[16]
25-49	2001	2.0% (n=1602)	(1.0-4.3%)	0.5-8.0%	[18]
25-49	2006	1.1% (n=2121)	(0.5-1.8%)	0.2-5.0%	[17]
25-49	2013	1.1% (n=2293)	(0.6-1.6%)	0.2-2.5%	[16]
50-59	2006	1.2% (n=488)	(0.0-2.4%)	0.0-7.0%	[17]
50-59	2013	1.2% (n=551)	(0.0-2.3%)	0.0-3.5%	[16]
**HIV prevalence among all female sex workers**					
15-59	1987	36.0% (n=103)	N.A.	5.0-85.0%	[98]
15-59	1995	46.0% (n=176)	(38.8-53.4%)	20.0-85.0%	[127]
15-59	1997	30.4% (n=191)	(24.3-37.2%)	15.0-80.0%	[99]
15-59	2000	28.9% (n=200)	N.A.	13.0-70.0%	[72]. Sample size not available and assumed n=200.
15-59	2003	31.9% (n=200)	N.A.	15.0-60.0%	As above
15-59	2006	35.3% (n=200)	N.A.	15.0-70.0%	As above
15-59	2009	24.2% (n=433)	(21.4-27.2%)	12.0-60.0%	[72]
15-59	2013	18.3% (n=388)	(14.8-22.5%)	10.0-50.0%	[102]
15-59	2017	20.8% (n=303)	(16.6-25.7%)	10.0-40.0%	[128]
15-59	2018	20.4% (n=353)	(16.3-25.0%)	10.0-40.0%	[103]
15-59	2019	8.7% (n=1253)	(7.3-10.4%)	4.0-30.0%	[15]
**HIV prevalence among clients of sex workers**					
15-59	2009	2.7% (n=731)	(1.8-4.2%)	Only used for comparison	[72]. This study was among truck drivers, which do not well represent FSW clients.
15-59	2019	1.9% (n=1104)	(1.2-2.9%)	Only used for comparison	[15]. This study was among truck drivers, which do not well represent FSW clients.
**HIV Prevalence among MSM**					
All 15-59	2011	20.1% (n=200)	N.A.	5.0-70.0%	[7] (original source not found, sample size not available and assumed n=200)
All 15-59	2015	18.1% (n=552)	(15.1-21.5%)	3.0-32.0%	[50]
All 15-59	2015	9.5% (n=613)	(5.6-13.5%)	3.0-32.0%	[48]
All 15-24	2020	8.5% (n=613)	(6.5-11.0%)	4.0-15.0%	[33]
All 25-49	2020	19.1% (n=418)	(15.6-23.1%)	10.0-28.0%	[33]
**HIV incidence rate (per 100 susceptible-year)**					
15-59	1990	0.255	(0.128-0.398)	0.03-1.40	[67]
15-59	1995	0.232	(0.179-0.310)	0.06-0.36	As above
15-59	2000	0.133	(0.105-0.167)	0.05-0.30	As above
15-59	2005	0.092	(0.071-0.115)	0.03-0.21	As above
15-59	2010	0.066	(0.049-0.086)	0.03-0.19	As above

15-59	2015	0.048	(0.032-0.067)	0.01-0.17	As above
15-59	2020	0.030	(0.018-0.052)	0.01-0.06	As above
**Number of new HIV infections**					
15-59	1990	11000	(5600-17000)	2600-37000	[67]
15-59	1995	11000	(8700-15000)	2700-35000	As above
15-59	2000	7400	(5800-9300)	1800-25300	As above
15-59	2005	5900	(4500-7400)	1200-14000	As above
15-59	2010	4900	(3600-6400)	1000-9000	As above
15-59	2015	4100	(2800-5800)	800-8000	As above
15-59	2020	3100	(1800-5200)	1800-7000	As above
**Number of HIV-related deaths**					
15-59	1990	1100	(0-3200)	0-8200	[67]
15-59	1995	3400	(1600-6700)	500-12300	As above
15-59	2000	6000	(4100-8500)	2100-15500	As above
15-59	2005	6400	(5100-8000)	2100-16000	As above
15-59	2010	4200	(3300-5400)	1300-9400	As above
15-59	2015	4300	(3400-5400)	1400-9400	As above
15-59	2020	3100	(2200-4300)	1500-5500	As above
**Fraction of all females ever tested for HIV**					
15-49	2001	4.2% (n=12837)	(3.5-5.0%)	1.0-20.0%	[18]
15-49	2009	14.4% (n=26717)	(13.3-15.6%)	5.0-50.0%	[129]
15-49	2015	19.9% (n=18409)	(18.0-21.9%)	8.0-50.0%	[130]
15-49	2018	17.9% (n=10519)	(15.8-20.1%)	10.0-50.0%	[59]
**Fraction of all females not living with HIV ever tested for HIV**					
15-49	2006	6.2% (n=4644)	(5.2-7.5%)	2.0-25.0%	[17]
15-49	2013	12.4% (n=5044)	(10.6-14.3%)	5.0-45.0%	[16]
**Fraction of all females living with HIV ever tested for HIV**					
15-49	2006	16.9% (n=69)	N.A.	5.0-50.0%	[126]
15-49	2013	36.5% (n=66)	N.A.	20.0-60.0%	As above
15-49	2020	54.7% (n=100)	N.A.	40.0-70.0%	As above
**Fraction of all males ever tested for HIV**					
15-49	2001	9.0% (n=3053)	(7.3-10.9%)	1.0-40.0%	[18]
15-49	2015	17.3% (n=7428)	(15.7-19.0%)	5.0-45.0%	[130]
15-49	2018	14.7% (n=4618)	(32.1-37.1%)	8.0-30.0%	[59]
**Fraction of all males not living with HIV ever tested for HIV**					
15-49	2006	6.4% (n=3449)	(5.3-7.8%)	2.0-30.0%	[17]
15-49	2013	10.7% (n=3304)	(9.1-12.6%)	4.0-30.0%	[16]
**Fraction of all males living with HIV ever tested for HIV**					
15-49	2006	16.4% (n=34)	N.A.	8.0-45.0%	[126]
15-49	2013	33.7% (n=25)	N.A.	15.0-60.0%	As above
15-49	2020	49.8% (n=100)	N.A.	35.0-65.0%	As above
**Fraction of FSW ever tested for HIV**					
15-59	2000	35.9% (n=131)	N.A.	2.0-70.0%	[100]
15-59	2003	30.5% (n=133)	N.A.	10.0-60.0%	As above
15-59	2006	50.8% (n=200)	N.A.	25.0-80.0%	As above
15-59	2009	67.3% (n=409)	N.A.	30.0-90.0%	As above
15-59	2018	45.8% (n=72)	(34.8-57.3%)	20.0-85.0%	[103] (Sample of FSW living with HIV)
**Fraction of MSM ever tested for HIV**					
15-59	2015	71.6% (n=522)	N.A.	50.0-95.0%	[50]
15-59	2019	83.2% (n=1031)	(80.8-85.4%)	60.0-90.0%	[33]
**Fraction of all females living with HIV which are diagnosed**					
15-59	2000	5.5% (n=500)	(4.6-6.6%)	1.0-15.0%	[126]. No sample size available but was assumed n=500.
15-59	2005	11.6% (n=500)	(9.7-14.0%)	5.0-25.0%	As above
15-59	2010	30.4% (n=500)	(25.3-36.5%)	15.0-50.0%	As above
15-59	2015	37.8% (n=500)	(31.5-45.4%)	20.0-60.0%	As above
15-59	2020	51.3% (n=500)	(42.8-61.6%)	30.0-70.0%	As above
**Fraction of all males living with HIV which are diagnosed**					

15-59	2000	5.3% (n=500)	(4.4-6.4%)	1.0-15.0%	As above
15-59	2005	11.2% (n=500)	(9.3-13.5%)	5.0-25.0%	As above
15-59	2010	28.8% (n=500)	(24.0-34.6%)	15.0-50.0%	As above
15-59	2015	34.9% (n=500)	(29.1-41.9%)	20.0-60.0%	As above
15-59	2020	45.5% (n=500)	(37.9-54.6%)	30.0-70.0%	As above
**Fraction of all FSW living with HIV with a diagnosed infection**					
15-59	2018	45.8% (n=72)	(34.8-57.3%)	28.0-70.0%	[103]
**Fraction of all MSM living with HIV with a diagnosed infection**					
15-59	2014	34.1% (n=79)	(15.6-52.5%)	15.0-65.0%	[48]
**Fraction of all females living with HIV with a treated infection**					
15-49	2015	39.0%	(33.0-47.0%)	20.0-60.0%	[67]. No sample size available.
15-49	2020	62.0%	(51.0-76.0%)	45.0-82.0%	As above
**Fraction of all males living with HIV with a treated infection**					
15-49	2015	25.0%	(21.0-30.0%)	10.0-50.0%	As above
15-49	2020	44.0%	(35.0-53.0%)	30.0-60.0%	As above
**Fraction of all MSM living with HIV with a treated infection**					
15-59	2014	30.0% (n=79)	(18.3-45.0%)	10.0-60.0%	[48]
**Fraction of FSW living with HIV with a suppressed viral load**					
15-49	2019	51.6% (n=93)	(41.3-61.7%)	30.0-70.0%	[15]
**Fraction of MSM living with HIV with a suppressed viral load**					
15-49	2014	30.0% (n=79)	(18.3-45.0%)	10.0-60.0%	[48]
15-24	2020	40.0% (n=52)	(27.5-44.8%)	20.0-65.0%	[33]
25-49	2020	48.1% (n=79)	(37.0-59.2%)	30.0-70.0%	[33]
**Number of conventional HIV tests done each year (females and males combined)**					
15-59	2015	393,007	N.A.	(196,504-786,014)	Programmatic data reported by countries to UNAIDS's Shiny90[126]
15-59	2016	400,005	N.A.	(200,003-800,010)	As above
15-59	2017	476,098	N.A.	(238,049-952,196)	As above
15-59	2018	565,838	N.A.	(282,919-1,131,676)	As above
15-59	2019	504,414	N.A.	(252,207-1,008,828)	As above
**Fraction of conventional HIV tests done which are positive (females and males combined)**					
15-59	2015	2.3%	N.A.	(1.2-4.6%)	As above
15-59	2016	1.7%	N.A.	(0.9-3.5%)	As above
15-59	2017	2.6%	N.A.	(1.3-5.1%)	As above
15-59	2018	2.3%	N.A.	(1.2-4.6%)	As above
15-59	2019	2.5%	N.A.	(1.2-5.0%)	As above

MSMW: men who have sex with men as well as female partners; MSME: men who have sex with men exclusively.
N.A.: Not available

Fitting data (Senegal)

Table S2c: List of demographic, epidemiological, and intervention outcomes used for model fitting in Senegal

Population or age group	Year	Point estimate (sample size used for simulation likelihood calculation)	Original 95%CI	Prior constraint	Reference
**Population size**					
Total number of 15-59years-old	1970<br>1980<br>1990<br>2000<br>2010<br>2020	2.18 million<br>2.74 million<br>3.64 million<br>4.92 million<br>6.56 million<br>8.81 million	N.A.	Initial value for 1970 and direct calibration using growth rate between 1970 and 2020 estimates	From [5]
**Age distribution among 15–59-year-old females**					
	1970	15-24 years: 35.7%<br>25-49 years: 53.0%<br>50-59 years: 11.3%	N.A.	Used for comparison	From [5]. This data was only used for comparison because we used UNPD estimates for the year 1970 combining female and males as model input (Table S1a), and fitted our model to sex-specific UNPD estimates for 2020.
	1980	15-24 years: 37.2%<br>25-49 years: 51.4%<br>50-59 years: 11.4%	N.A.	Used for comparison	As above
	1990	15-24 years: 39.3%<br>25-49 years: 49.8%<br>50-59 years: 10.9%	N.A.	Used for comparison	As above
	2000	15-24 years: 40.4%<br>25-49 years: 50.1%<br>50-59 years: 9.5%	N.A.	Used for comparison	As above
	2010	15-24 years: 37.8%<br>25-49 years: 52.5%<br>50-59 years: 9.7%	N.A.	Used for comparison	As above
	2020	15-24 years: 35.6%<br>25-49 years: 54.2%<br>50-59 years: 10.2%	N.A.	27.4-46.3%<br>41.6-70.5%<br>6.8-15.3%	Fitted from [5]
**Age distribution among 15–59-year-old males**					
	1970	15-24 years: 35.7%<br>25-49 years: 53.8%<br>50-59 years: 10.5%	N.A.	Used for comparison	From [5]. This data was only used for comparison because we used UNPD estimates for the year 1970 combining female and males as model input (Table S1a), and fitted our model to sex-specific UNPD estimates for 2020.
	1980	15-24 years: 37.3%<br>25-49 years: 51.6%<br>50-59 years: 11.1%	N.A.	Used for comparison	As above
	1990	15-24 years: 40.4%<br>25-49 years: 48.8%<br>50-59 years: 10.8%	N.A.	Used for comparison	As above

	2000	15-24 years: 42.8%<br>25-49 years: 48.2%<br>50-59 years: 9.0%	N.A.	Used for comparison	As above
	2010	15-24 years: 41.4%<br>25-49 years: 50.0%<br>50-59 years: 8.6%	N.A.	Used for comparison	As above
	2020	15-24 years: 39.1%<br>25-49 years: 52.3%<br>50-59 years: 8.6%	N.A.	30.1-50.8%<br>40.2-68.0%<br>5.7-12.9%	Fitted from [5]
**HIV prevalence among all adult females (except female sex workers)**					
15-24	2005	0.5% (n=1991)	(0.1-0.8%)	0.1-3.0%	[22]
15-24	2011	0.3% (n=2432)	(0.1-0.5%)	0.1-3.0%	[23]
15-24	2017	0.2% (n=3353)	(0.0-0.5%)	0.0-1.0%	[25]
25-49	2005	0.8% (n=2475)	(0.4-1.3%)	0.4-6.0%	[22]
25-49	2011	0.8% (n=3158)	(0.4-1.1%)	0.4-5.0%	[23]
25-49	2017	0.7% (n=4612)	(0.5-1.3%)	0.5-1.5%	[25]
**HIV prevalence among all adult males**					
15-24	2005	0.1% (n=1482)	(0.0-0.2%)	0.0-1.5%	[22]
15-24	2011	0.1% (n=1916)	(0.0-0.1%)	0.0-1.5%	[23]
15-24	2017	0.1% (n=2736)	(0.0-0.2%)	0.0-1.0%	[25]
25-49	2005	0.7% (n=1466)	(0.1-1.2%)	0.1-5.0%	[22]
25-49	2011	0.7% (n=1956)	(0.3-1.2%)	0.1-5.0%	[23]
25-49	2017	0.6% (n=2994)	(0.3-1.0%)	0.1-2.0%	[25]
50-59	2011	0.8% (n=455)	(0.1-1.4%)	0.0-5.0%	[23]
50-59	2017	1.2% (n=615)	(0.3-2.1%)	0.0-3.0%	[25]
**HIV prevalence among all female sex workers**					
15-59	1988	16.1% (n=1710)	(14.5-18.0%)	5.0-60.0%	[131]
15-59	1989	3.1% (n=200)	N.A.	1.0-60.0%	[132]. Sample size not available
15-59	1994	10.1% (n=200)	N.A.	2.0-60.0%	As above
15-59	2000	20.1% (n=1296)	(18.0-22.4%)	5.0-60.0%	[133]
15-59	2006	19.8% (n=618)	(16.8-23.1%)	5.0-60.0%	[76]
15-59	2010	18.5% (n=672)	N.A.	5.0-50.0%	[75]
15-59	2015	6.6% (n=694)	(5.4-8.7%)	2.0-30.0%	[74]
15-59	2015	3.3% (n=758)	(1.5-5.2%)	2.0-30.0%	[78]
15-59	2017	8.1% (n=173)	N.A.	3.0-40.0%	[109]
15-59	2019	5.8% (n=1749)	(4.0-7.0%)	10.0-40.0%	[73]
**HIV prevalence among clients of sex workers**					
15-59	1999	4.4% (n=1071)	(3.3-5.8%)	1.0-30.0%	[134]
15-59	2015	1.2% (n=600)	(0.2-2.5%)	0.5-10.0%	IBBS surveys [78] (personal communication of estimated from the unpublished client survey)
**HIV Prevalence among MSM**					
All 15-59	2012	38.6% (n=114)	(30.2-47.8%)	15.0-60.0%	[135]
All 15-59	2016	23.5% (n=724)	(18.6-28.4%)	10.0-40.0%	[78] (RDS-adjusted estimates)
All 15-24	2014	17.7% (n=645)	(14.9-20.8%)	5.0-30.0%	[55]
All 15-24	2017	19.4% (n=690)	(16.6-22.5%)	5.0-35.0%	[56] (data fitted among MSMW/MSME separately)
All 25-49	2014	18.1% (n=365)	(14.5-22.4%)	5.0-50.0%	[55]
All 25-49	2017	39.5% (n=441)	(35.0-44.1%)	16.0-60.0%	[56]
All MSMW	2004	20.2% (n=401)	(16.6-24.4%)	5.0-60.0%	[51]
All MSMW	2007	19.3% (n=357)	(15.5-23.7%)	5.0-50.0%	[79]
All MSMW	2017	24.1% (n=882)	(21.4-27.0%)	15.0-35.0%	[56]
All MSME	2004	34.1% (n=41)	(21.5-49.4%)	5.0-70.0%	[51]
All MSME	2007	29.0% (n=131)	(21.9-37.3%)	13.0-70.0%	[79]
All MSME	2017	37.7% (n=266)	(32.0-43.7%)	25.0-50.0%	[56]
**HIV incidence rate (per 100 susceptible-year)**					
15-59	1990	0.036	(0.028-0.046)	0.010-0.12	[67]
15-59	1995	0.072	(0.058-0.088)	0.018-0.160	As above
15-59	2000	0.094	(0.080-0.112)	0.030-0.192	As above

15-59	2005	0.064	(0.052-0.076)	0.022-0.146	As above
15-59	2010	0.018	(0.014-0.022)	0.007-0.052	As above
15-59	2015	0.010	(0.008-0.013)	0.004-0.045	As above
15-59	2020	0.010	(0.088-0.014)	0.004-0.030	As above
**Number of new HIV infections**					
15-59	1990	1400	(1100-1800)	300-4500	[67]
15-59	1995	3300	(2600-3900)	1600-7000	As above
15-59	2000	4900	(4100-5800)	2100-9800	As above
15-59	2005	3800	(3100-4500)	2100-8500	As above
15-59	2010	1200	(0-1500)	0-3500	As above
15-59	2015	500	(0-1000)	0-3000	As above
15-59	2020	700	(0-1300)	0-2600	As above
**Number of HIV-related deaths**					
15-59	1990	100	(0-500)	0-2000	[67]
15-59	1995	500	(0-1000)	0-3000	As above
15-59	2000	1300	(1100-1700)	500-3700	As above
15-59	2005	2400	(2000-2900)	1000-3900	As above
15-59	2010	1200	(0-1600)	0-3000	As above
15-59	2015	1600	(1200-2000)	600-4000	As above
15-59	2020	800	(0-1000)	0-1500	As above
**Fraction of all females ever tested for HIV**					
15-49	2000	3.0% (n=11793)	(2.4-3.8%)	0.0-20.0%	[136]
15-49	2014	42.6% (n=8488)	(40.0-45.2%)	25.0-80.0%	[28]
15-49	2015	42.6% (n=8851)	(40.1-45.1%)	25.0-80.0%	[27]
15-49	2016	39.8% (n=8865)	(36.8-42.8%)	10.0-50.0%	[26]
**Fraction of all females not living with HIV ever tested for HIV**					
15-49	2005	2.8% (n=4408)	(2.2-3.6%)	0.5-20.0%	[22]
15-49	2010	27.3% (n=5529)	(25.3-29.4%)	15.0-70.0%	[137]
15-49	2017	46.1% (n=7909)	(44.1-48.1%)	32.0-80.0%	[25]
**Fraction of all females living with HIV ever tested for HIV**					
15-49	2005	6.7% (n=48)	N.A.	2.0-40.0%	[126]
15-49	2010	50.0% (n=61)	N.A.	25.0-80.0%	As above
15-49	2017	82.2% (n=56)	N.A.	70.0-99.0%	As above
**Fraction of all males ever tested for HIV**					
15-49	2014	20.0% (n=3067)	(17.5-22.7%)	12.0-70.0%	[28]
15-49	2015	21.8% (n=3445)	(19.2-24.7%)	12.0-70.0%	[27]
15-49	2016	19.8% (n=3236)	(17.3-22.4%)	10.0-70.0%	[26]
15-49	2018	17.9% (n=3134)	(15.8-20.1%)	10.0-60.0%	[24]
**Fraction of all males not living with HIV ever tested for HIV**					
15-49	2005	3.7% (n=2977)	(2.8-4.9%)	1.0-20.0%	[22]
15-49	2010	16.7% (n=3919)	(15.1-18.6%)	5.0-60.0%	[137]
15-49	2017	19.0% (n=5790)	(17.3-20.8%)	15.0-70.0%	[25]
**Fraction of all males living with HIV ever tested for HIV**					
15-49	2005	4.9% (n=14)	N.A.	1.0-40.0%	[126]
15-49	2010	30.0% (n=24)	N.A.	10.0-80.0%	As above
15-49	2017	55.7% (n=27)	N.A.	35.0-90.0%	As above
**Fraction of FSW ever tested for HIV**					
15-59	2006	63.2% (n=618)	N.A.	10.0-90.0%	[76]
15-59	2010	73.6% (n=694)	N.A.	20.0-95.0%	[75]
15-59	2013	58.0% (n=500)	N.A.	25.0-90.0%	[40]. Sample size not available and assumed n=500.
15-59	2015	72.4% (n=550)	(68.5-76.0%)	30.0-95.0%	[78]
15-59	2018	79.4% (n=155)	(72.3-85.0%)	40.0-95.0%	[138]
**Fraction of MSM ever tested for HIV**					
15-59	2003	13.3% (n=158)	(8.9-19.5%)	3.0-60.0%	[106]
15-59	2004	10.8% (n=463)	(8.1-14.0%)	3.0-50.0%	[51]
15-59	2007	34.1% (n=501)	(30.1-38.4%)	10.0-70.0%	[74,79]
15-59	2012	86.6% (n=119)	(79.3-91.6%)	55.0-99.0%	[135]
15-59	2014	72.6% (n=3649)	N.A.	30.0-90.0%	[139]

15-59	2014	69.1% (n=1012)	N.A.	50.0-90.0%	[74]
15-59	2015	70.2% (n=727)	(66.7-74.4%)	50.0-95.0%	[78]
15-59	2017	82.6% (n=1148)	(80.2-84.7%)	60.0-95.0%	[56]
15-59	2018	54.0% (n=174)	(46.6-61.3%)	40.0-90.0%	[138]
**Fraction of all females living with HIV which are diagnosed**					
15-59	2000	2.0% (n=500)	(1.6-2.4%)	1.0-10.0%	[126]. No sample size available but was assumed n=500.
15-59	2005	4.7% (n=500)	(4.0-5.7%)	1.0-20.0%	As above
15-59	2010	47.5% (n=500)	(39.6-57.0%)	30.0-70.0%	As above
15-59	2015	69.9% (n=500)	(58.3-83.9%)	50.0-95.0%	As above
15-59	2020	92.0% (n=500)	(76.7-99.0%)	75.0-99.0%	As above
**Fraction of all males living with HIV which are diagnosed**					
15-59	2000	1.3%	(1.1-1.6%)	1.0-10.0%	As above
15-59	2005	3.3% (n=500)	(2.8-4.0%)	1.0-15.0%	As above
15-59	2010	27.4% (n=500)	(22.0-32.9%)	10.0-50.0%	As above
15-59	2015	45.7% (n=500)	(38.1-54.8%)	30.0-70.0%	As above
15-59	2020	65.5% (n=500)	(54.6-78.6%)	50.0-90.0%	As above
**Fraction of all FSW living with HIV with a diagnosed infection**					
15-59	2000	5.1% (n=39)	(1.4-16.9%)	0.0-40.0%	[140]
15-59	2002	29.3% (n=208)	(23.6-35.8%)	5.0-70.0%	[141]
15-59	2010	12.5% (n=128)	(7.8-19.3%)	5.0-80.0%	[75]
15-59	2015	53.8% (n=39)	(37.4-69.6%)	20.0-90.0%	[138]
15-59	2015	67.5% (n=40)	(52.0-79.9%)	20.0-90.0%	[142]
15-59	2016	55.0% (n=40)	(39.8-69.3%)	30.0-80.0%	[78]
**Fraction of all MSM living with HIV with a diagnosed infection**					
15-59	2014	48.8% (n=41)	(34.2-63.5%)	20.0-85.0%	[135]
15-59	2016	13.2% (n=219)	(9.4-18.4%)	5.0-80.0%	[78]
15-59	2018	63.4% (n=100)	N.A.	40.0-90.0%	[7]. No sample size available.
**Fraction of all females living with HIV with a treated infection**					
15-49	2015	56.0%	(50.0-63.0%)	20.0-90.0%	[67]. No sample size available.
15-49	2020	95.0%	(85.0-99.0%)	85.0-99.0%	As above
**Fraction of all males living with HIV with a treated infection**					
15-49	2015	35.0%	(31.0-39.0%)	10.0-80.0%	As above
15-49	2020	61.0%	(54.0-69.0%)	45.0-75.0%	As above
**Fraction of all FSW living with HIV with a treated infection**					
15-59	2016	37.5% (n=40)	(24.2-53.0%)	20.0-53.0%	[78]
**Fraction of all MSM living with HIV with a treated infection**					
15-59	2016	10.0% (n=219)	(24.2-53.0%)	20.0-53.0%	[78]
15-59	2019	38.0% (n=200)	N.A.	10.0-60.0%	[7], No sample size available.
**Fraction of FSW living with HIV with a suppressed viral load**					
15-49	2019	48.0% (n=100)	(38.5-57.7%)	35.0-60.0%	[73]
**Number of conventional HIV tests done each year (females and males combined)**					
15-59	2016	611,175	N.A.	(305,588-1,222,350)	Programmatic data reported by countries to UNAIDS's Shiny90[126]
15-59	2017	550,386	N.A.	(275,193-1,100,772)	As above
15-59	2018	669,438	N.A.	(334,719-1,338,876)	As above
15-59	2019	684,635	N.A.	(342,318-1,369,270)	As above
**Fraction of conventional HIV tests done which are positive (females and males combined)**					
15-59	2016	1.5%	N.A.	(0.7-2.9%)	As above
15-59	2017	1.6%	N.A.	(0.8-3.3%)	As above
15-59	2018	1.1%	N.A.	(0.5-2.1%)	As above
15-59	2019	1.2%	N.A.	(0.6-2.4%)	As above

MSMW: men who have sex with men as well as female partners; MSME: men who have sex with men exclusively.
N.A.: Not available

Definition of indicators

Estimating the impact of condom use and ART uptake by risk populations on HIV outcomes

The fractions of new HIV infections and HIV-related deaths averted by condom use, ART uptake, and both (AF_{t_0-t}, equation 1), among all and specific risk groups was estimated by comparing the predicted cumulative number of new HIV infections and HIV-related deaths over periods $[t_0 - t_1]$ between the baseline scenario (with existing intervention efficacies) ($CI_{t_0-t_1}(baseline)$) and a counterfactual scenario where interventions do not protect against acquisition (condoms) nor transmission (condoms and ART) and do not reduce disease progression (ART) among the relevant risk groups over the period [t_0, t] ($CI_{t_0-t_1}(no\ intervention)$).

Equation 1: $AF_{t_0-t_1} = \frac{CI_{t_0-t_1}(no\ intervention) - CI_{t_0-t_1}(baseline)}{CI_{t_0-t_1}(no\ intervention)}$

The sum of $AF_{t_0-t_1}$ estimates for separate interventions may exceed 100% as it accounts for secondary transmissions averted that may overlap for different interventions.

Estimating the sources of HIV acquisition, of direct HIV transmissions only and of both direct and indirect HIV transmission

We derived three indicators to describe the contribution of different risk groups to the HIV epidemic.

The distribution of acquired infections (indicator 2) is the fraction of all new infections over a time period which are acquired by a specific risk group. The distribution of directly transmitted infections (indicator 3) is the fraction of all new infections over a period which are directly transmitted by a specific risk group. The transmission population-attributable fractions ($tPAF_{t_0-t}$, indicator 4)[143], is the fraction of all new infections directly or indirectly transmitted by a specific risk group. It is calculated as the relative difference between the cumulative number of new infections over the period [t_0, t]between the baseline scenario $CI_{t_0-t_1}(baseline)$ and a counterfactual scenario assuming that the relevant group cannot transmit HIV ($CI_{t_0-t_1}(no\ risk)$, equation 2)

Equation 2: $tPAF_{t_0-t_1} = \frac{CI_{t_0-t_1}(baseline) - CI_{t_0-t_1}(no\ risk)}{CI_{t_0-t_1}(baseline)}$.

The sum of tPAF estimates from separate risk groups may exceed 100% as it accounts for onward secondary transmissions that may overlap for different groups[143]. This can be interpreted as the fractions of new infections that would be averted by a 100% effective intervention blocking all transmission from the relevant risk population.

Results: model fits (Côte d'Ivoire)

Demography

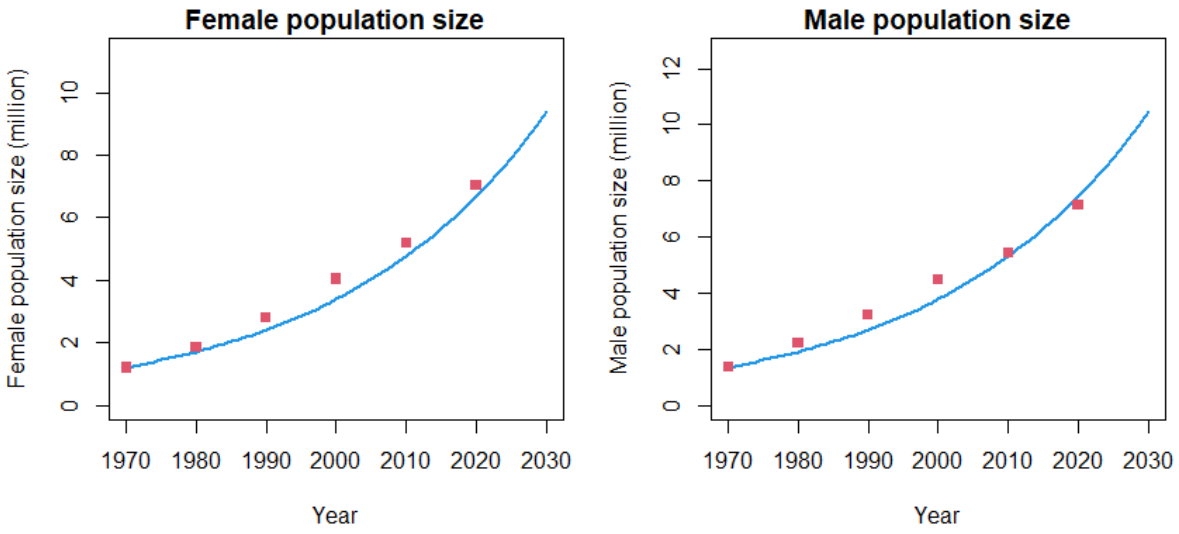

Figure S2a: Côte d'Ivoire model fitting to the size of the female and male population aged 15-59 years old over time. Blue curves represent model estimates and red squares the estimates from UNPD[5].

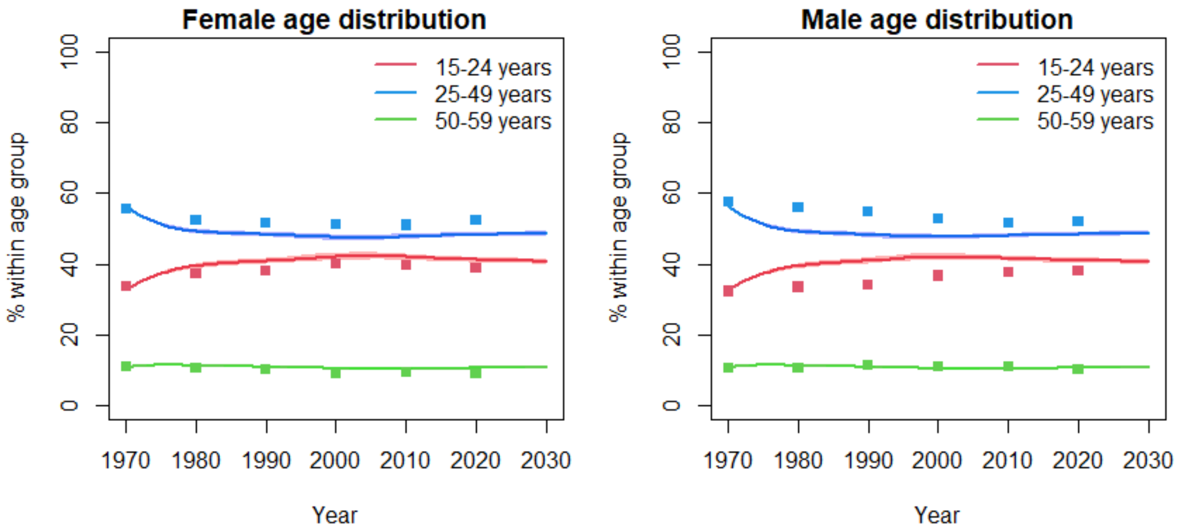

Figure S2b: Côte d'Ivoire model fitting to the age distribution of the female and male population aged 15-59 years old over time. Curves represent model estimates and the squares the estimates from UNPD. The latter were used to initialise the population age distribution in 1970 (combining males and females), and to calibrate the model to sex-specific estimates for 2020.

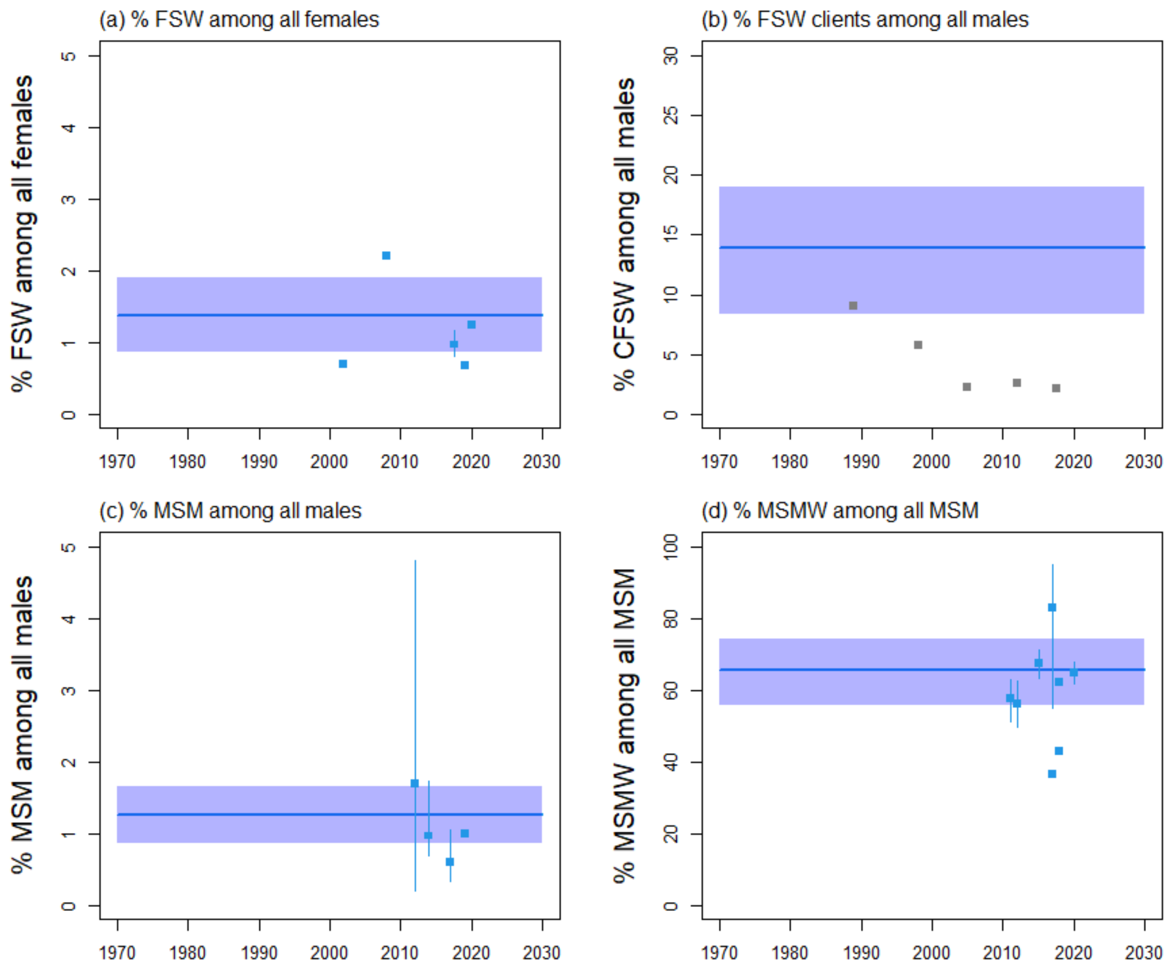

Figure S2c: Côte d'Ivoire model fitting to the size of key populations and their clients, with fractions of a) FSW among all females aged 15-59 years, b) FSW clients among all males aged 15-59 years, c) MSM among all males aged 15-59 years, and d) MSMW (men who have sex with men and women) among all MSM aged 15-59 years old over time. Blue curves and shades represent median and 90% UI (5thth and 95th percentiles across model fits), whereas squares and intervals represent empirical estimates (with 95% CI). Grey estimates in panel b) were reported from household surveys and were only used for comparison as they were deemed unrepresentative. The FSW clients population size in the model was calculated using the multiplier method as in [1,34,144,145].

HIV epidemiology

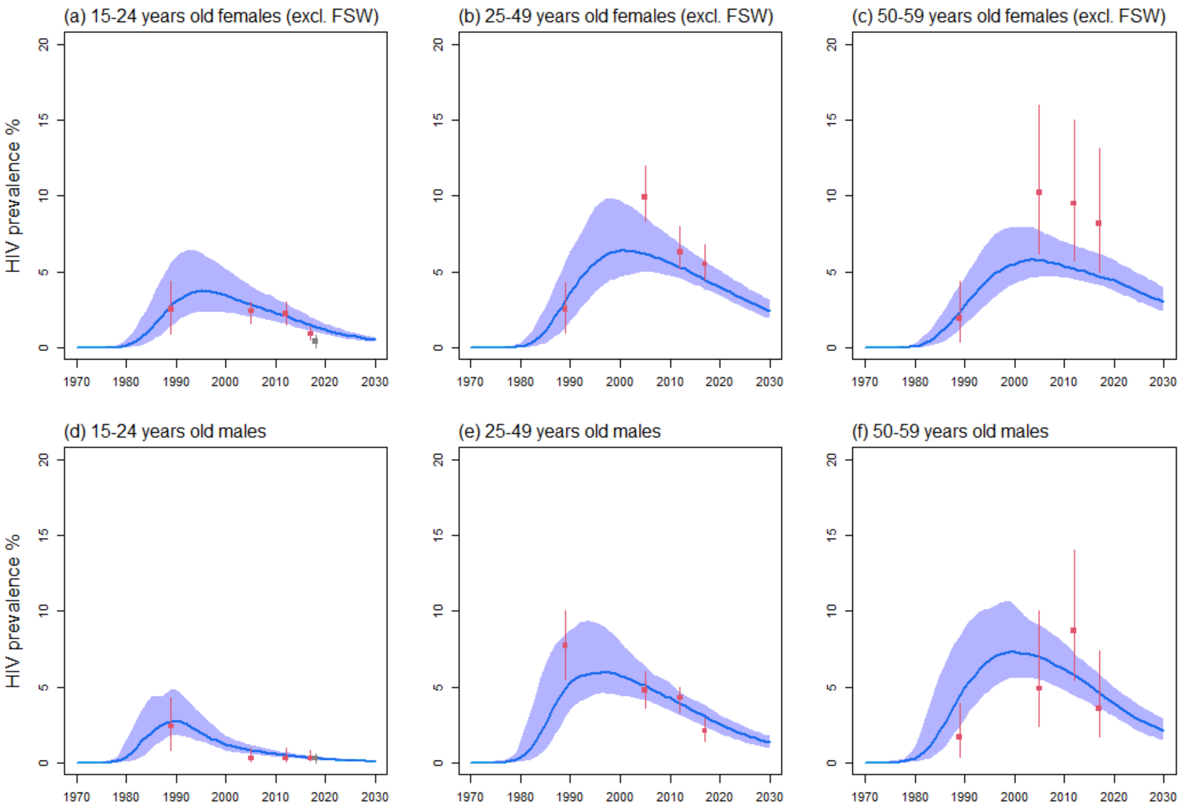

Figure S2d: Côte d'Ivoire model fitting to the HIV prevalence among all females aged a) 15-24, b) 25-49, and c) 50-59 years old (excluding FSW), as well as all males aged d) 15-24, e) 25-49 years, and f) 50-59 years old. Blue curves and shades represent median and 90% UI (5th and 95th percentiles across model fits), red squares and intervals represent empirical estimates used for model fitting (with 95% CI), and the grey square and intervals in panel a) are estimates from a recent survey about violence against children and youth in Côte d'Ivoire[112] included for comparison (not used to fit the model). Estimates among females aged 50-59 years old in 2005 and 2012 (panel c) should be interpreted with caution as they correspond to the estimate for the 45-49 years old age group as in[1] (these surveys did not include women aged 50+ years or older).

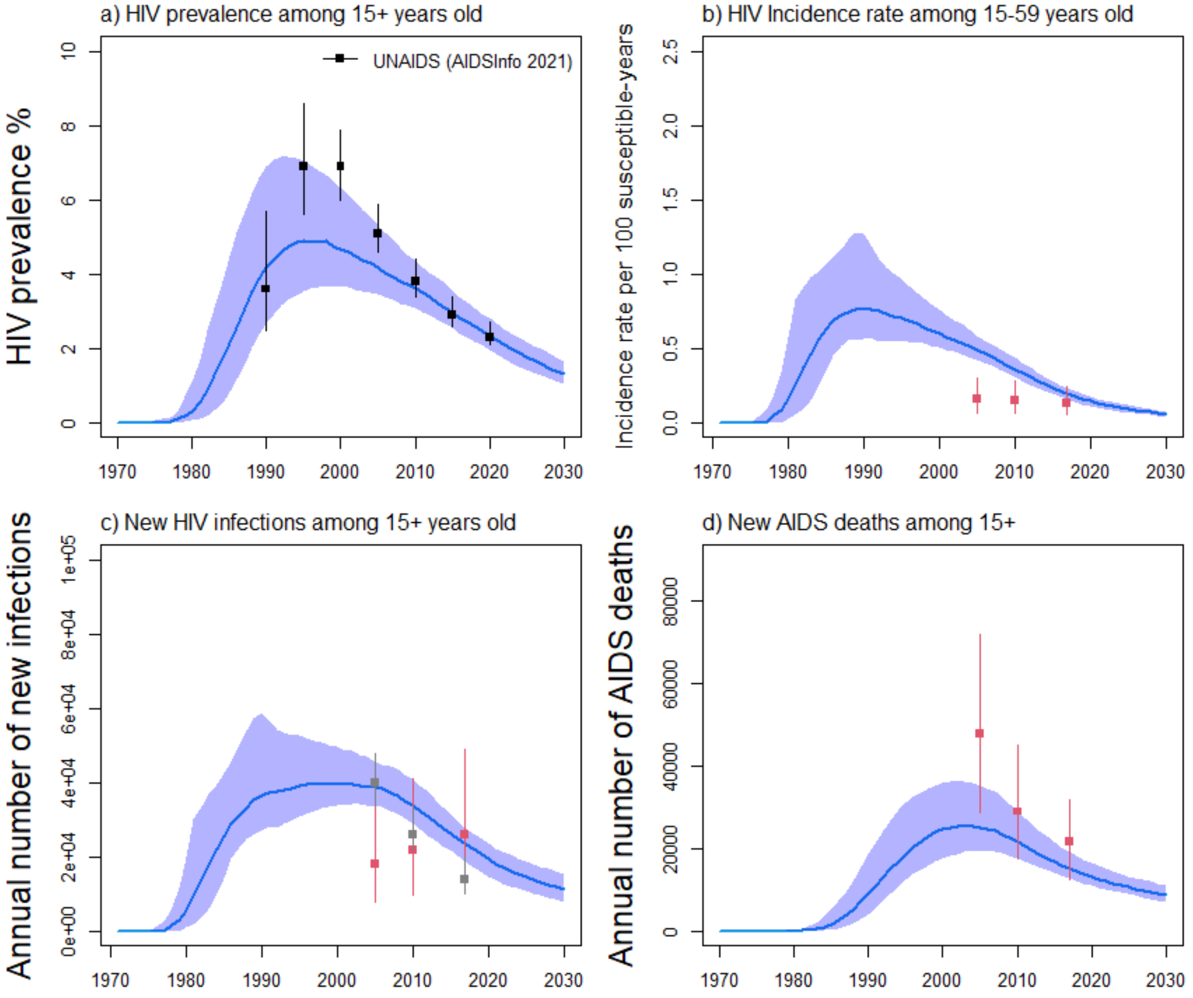

Figure S2e: Estimates of HIV prevalence among a) all adults aged over 15 years old, fits to b) HIV incidence rate per susceptible, c) annual number of new HIV infections and d) annual HIV-related deaths in Côte d'Ivoire from UNAIDS Data 2018 (from the Spectrum/EPP model[146]). Blue curves and shades represent median and 90% UI (5th and 95th percentiles across model fits), red squares and intervals represent empirical estimates used for model fitting (with 95%CI), dark squares and intervals in panel a) represent estimates from UNAIDS (also from the Spectrum/EPP model) used for comparison, whereas grey squares and intervals in panel c) represent new estimates from UNAIDS (Spectrum/EPP) which were published in July 2023 and not available at the time of our analysis.

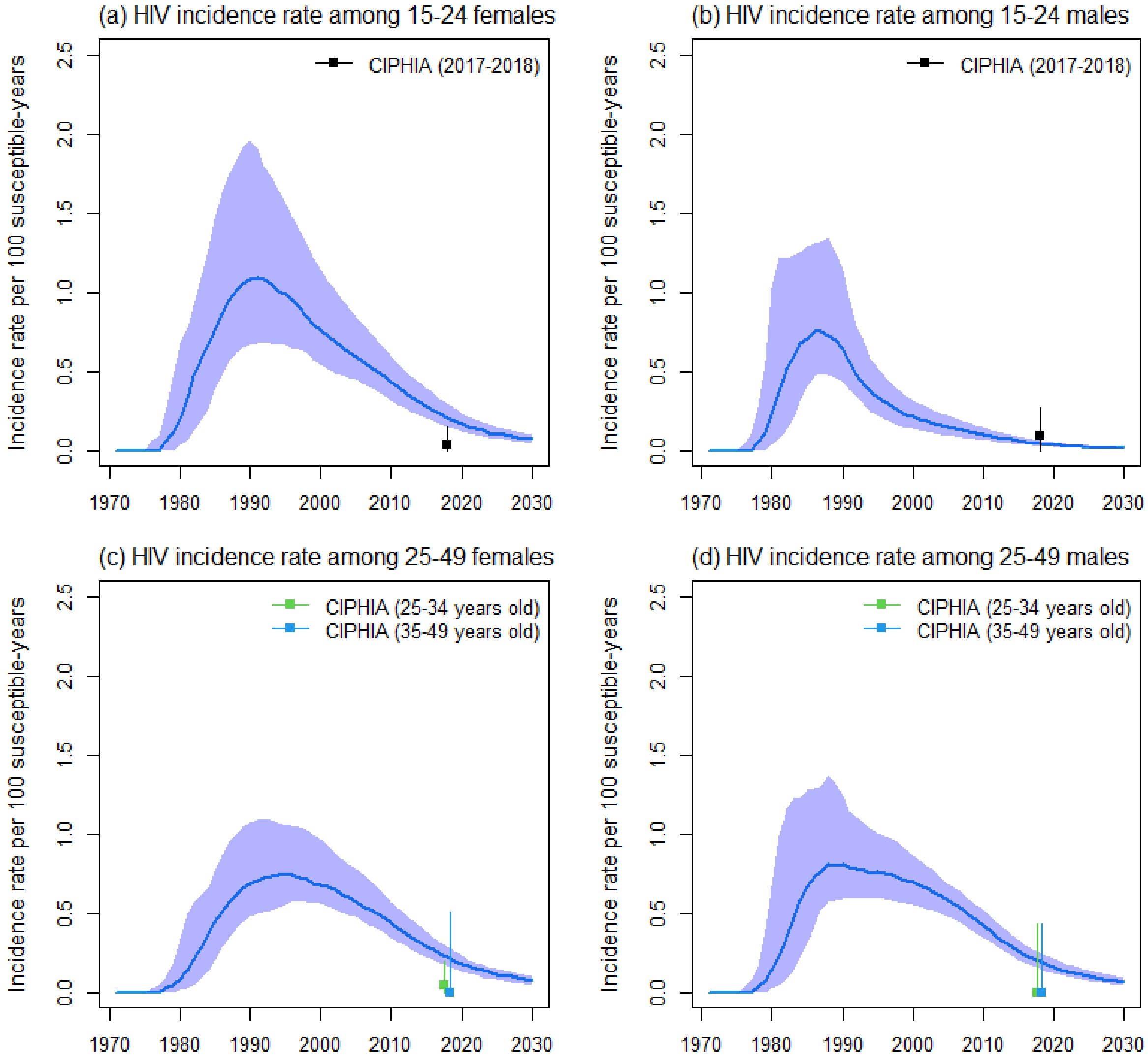

Figure S2f: Comparison of estimates of HIV incidence in the Côte d'Ivoire PHIA survey among a) females aged 15-24 years old, b) males aged 15-24 years old, c) females aged 25-49 years old, d) males aged 25-49 years old. Blue curves and shades represent median and 90% UI (5th and 95th percentiles across model fits), whereas squares and intervals represent empirical estimates from the PHIA survey (with 95%CI).

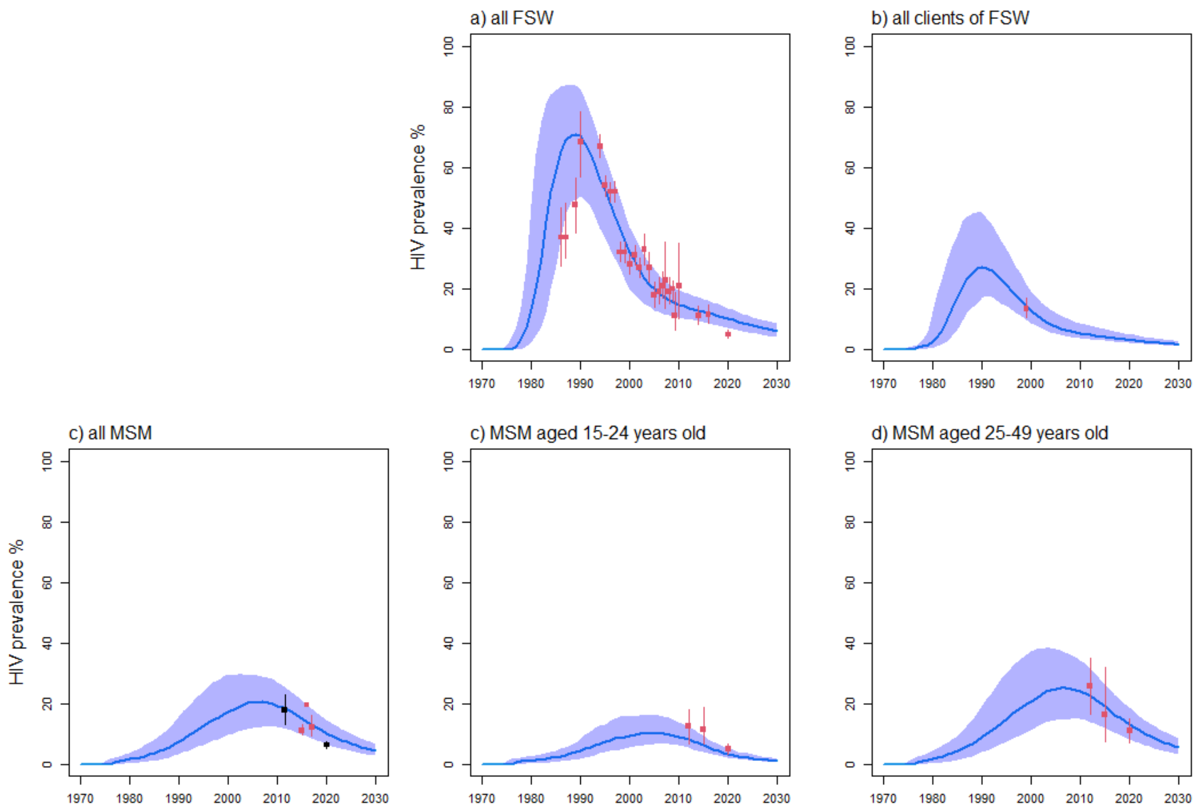

Figure S2g: Côte d'Ivoire model fitting to HIV prevalence estimates among all a) FSW, b) clients of FSW, c) MSM, as well as c) MSM aged 15-24 years old, and d) aged 25-49 years old. Blue curves and shades represent median and 90% UI (5th and 95th percentiles across model fits), red squares and intervals represent empirical estimates used for model fitting (with 95%CI), whereas dark estimates in panel c) represent estimates fitted among MSMW and MSME separately (see Figure S2h).

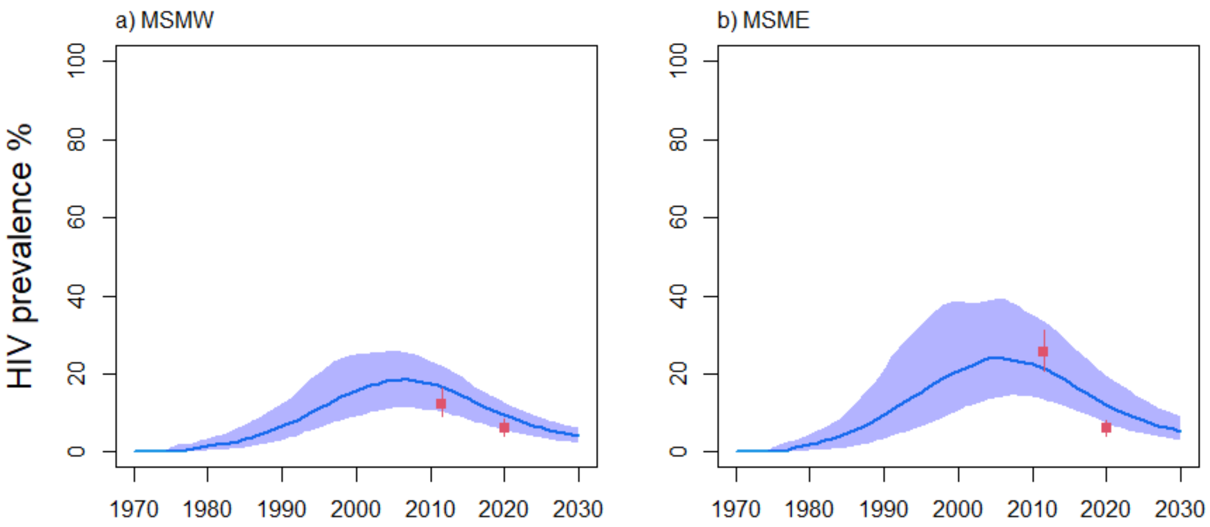

Figure S2h: Côte d'Ivoire model fitting to HIV prevalence estimates among all a) MSMW (men having sex with both men and women) and b) MSME (men having sex with another men) MSM. Blue

curves and shades represent median and 90% UI (5th and 95th percentiles across model fits), whereas red squares and intervals represent empirical estimates (with 95%CI).

HIV treatment cascade

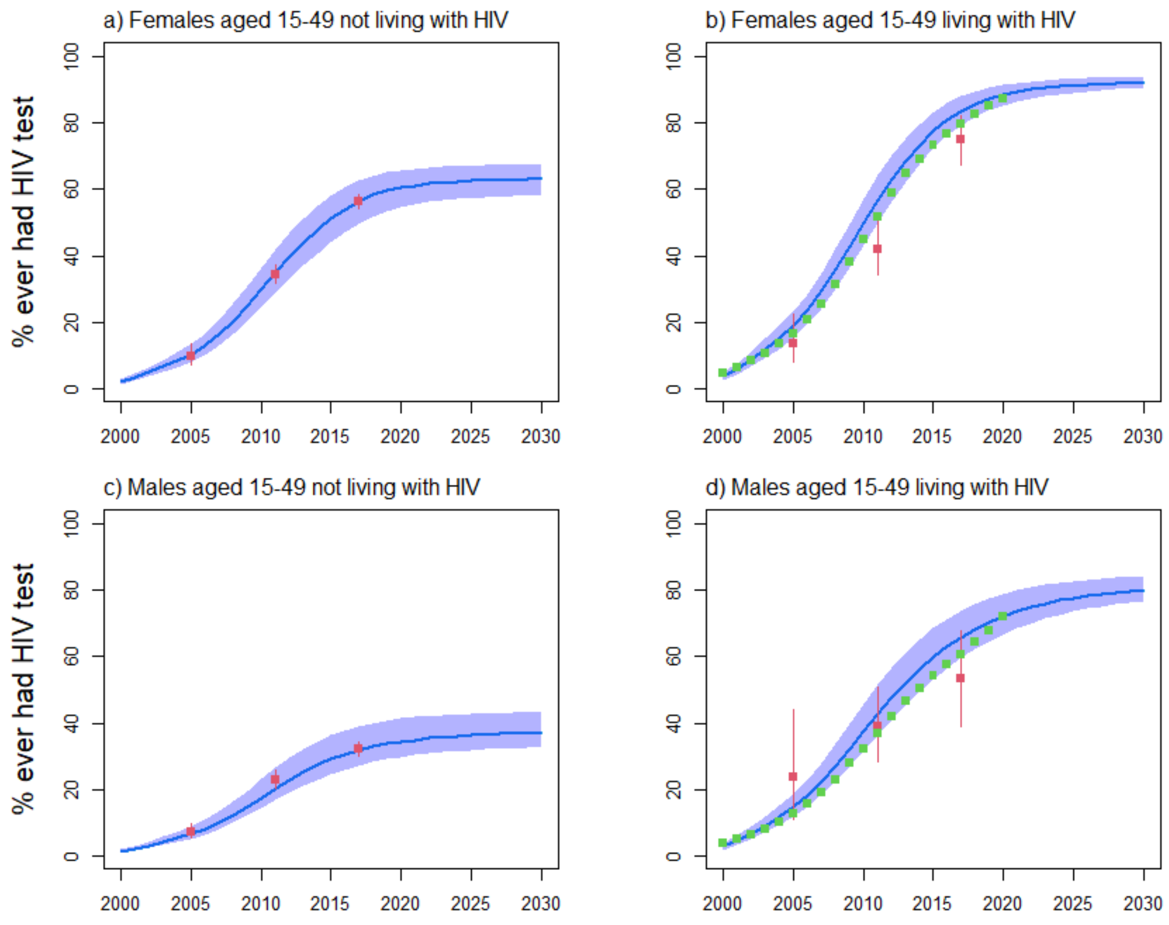

Figure S2i: Côte d'Ivoire model fitting to the fraction ever having tested for HIV among all females aged 15-49 years old a) not living with HIV, and b) living with HIV, and males aged 15-49 years old c) not living with HIV, and d) living with HIV. Blue curves and shades represent median and 90% UI (5th and 95th percentiles across model fits), red squares and intervals represent empirical estimates used for model fitting (with 95%CI), whereas green squares represent estimates from UNAIDS Shiny90 which were only used for comparison.

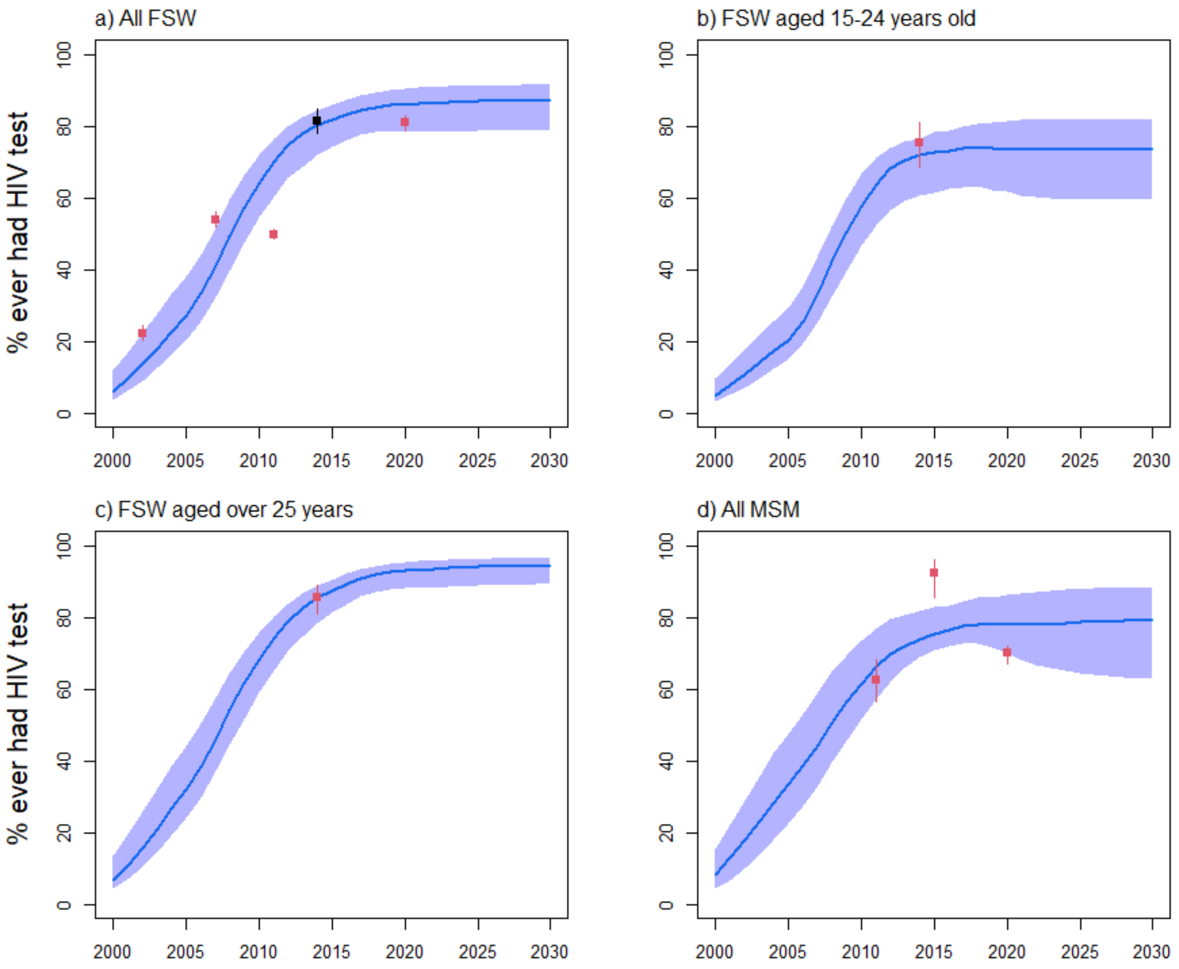

Figure S2j: Côte d'Ivoire model fitting to the fraction ever having tested for HIV among a) all FSW, b) FSW aged 15-24 years, c) FSW aged 25-49 years, and d) MSM. Blue curves and shades represent median and 90% UI (5th and 95th percentiles across model fits), red squares and intervals represent empirical estimates used for model fitting (with 95%CI), whereas dark squares in panel a) represent overall fraction from study outcomes shown in panels b) and c).

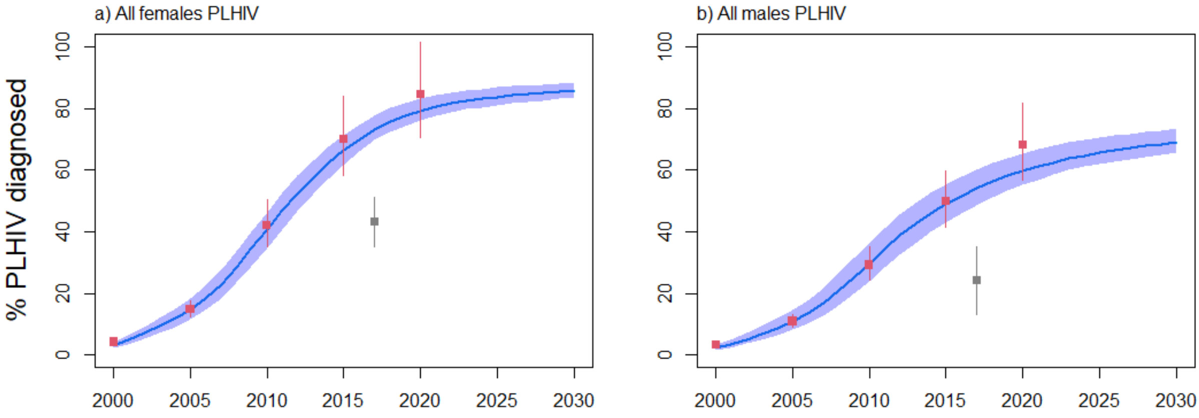

Figure S2k: Côte d'Ivoire model fitting to the fraction of a) all females living with HIV and b) all males living with HIV diagnosed (aware of their status). Blue curves and shades represent median and 90% UI (5th and 95th percentiles across model fits), red squares and intervals represent estimates from UNAIDS Shiny90 used for model fitting[126], whereas grey squares and intervals represent estimates from PHIA[32], which were underestimates because this fraction of people diagnosed (reporting being aware of their positive status) lower than the fraction of PLHIV having traces of ARV drugs in their blood in the survey.

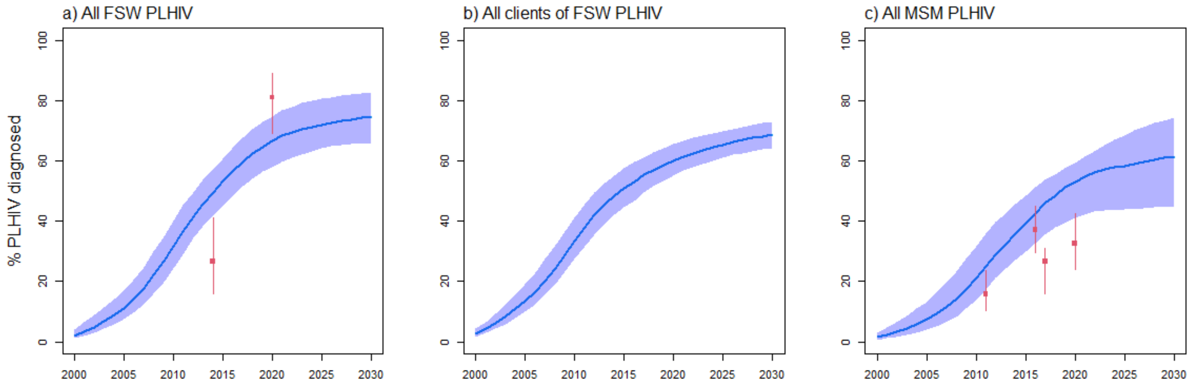

Figure S2l: Côte d'Ivoire model fitting to fraction of a) all FSW living with HIV, b) all male clients of FSW living with HIV, and c) all MSM living with HIV diagnosed. Blue curves and shades represent median and 90% UI (5th and 95th percentiles across model fits), whereas red squares and intervals represent estimates from empirical surveys (with 95%CI). Empirical estimates are self-reported and likely to be under-estimates.[147]

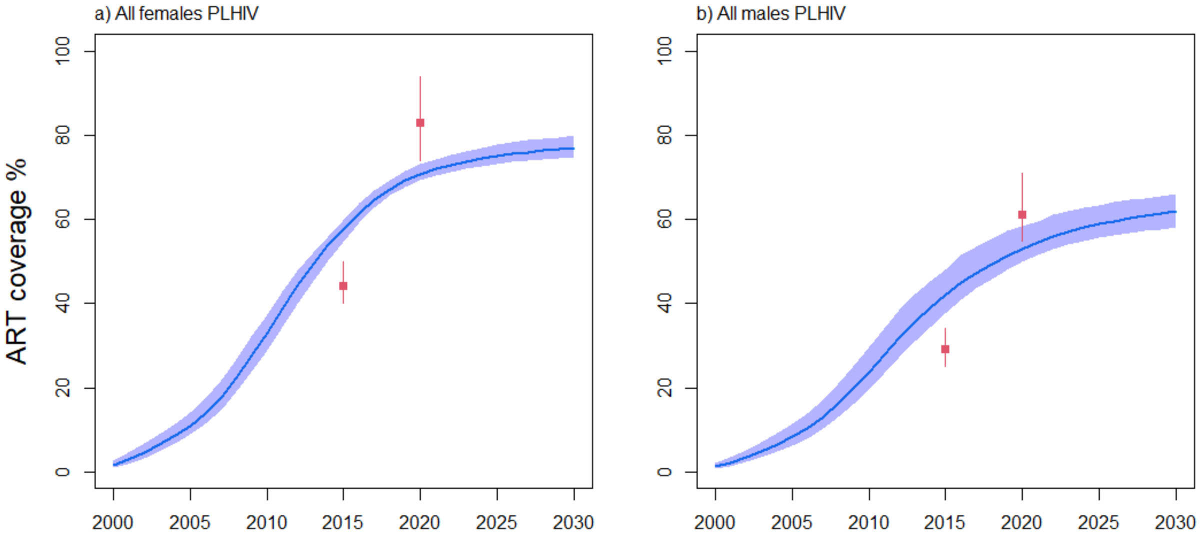

Figure S2m: Côte d'Ivoire model fitting to ART coverage among a) all females and b) all males aged 15-59 years old living with HIV. Blue curves and shades represent median and 90% UI (5thth and 95th percentiles across model fits), whereas red squares and intervals represent estimates from UNAIDS (with 95%CI), using the Spectrum/EPP model. Note that high coverages in red for the year 2020 could not be reproduced by our model as they don't agree well with estimates of the fraction of all females and all males PLHIV with a suppressed HIV viral load in the 2017 PHIA survey (see Table S2a and Figure S2o).

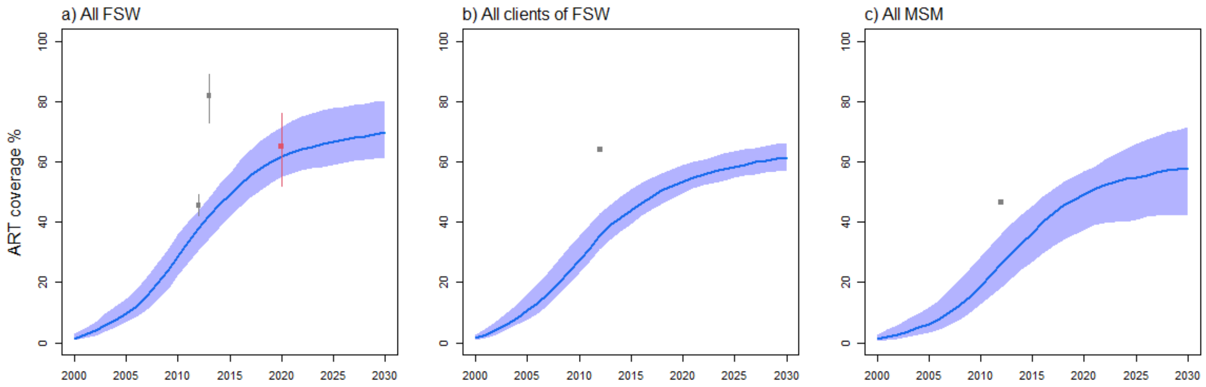

Figure S2n: Côte d'Ivoire model fitting to ART coverage among a) all FSW, b) all male clients of FSW, and c) all MSM living with HIV. Blue curves and shades represent median and 90% UI (5th and 95th percentiles across model fits), whereas red squares and intervals represent empirical estimates from local surveys (with 95%CI)[117]. Estimates in grey represent estimates from an STI clinic[115], which were assumed to be overestimates and not included for model fitting but shown for comparison.

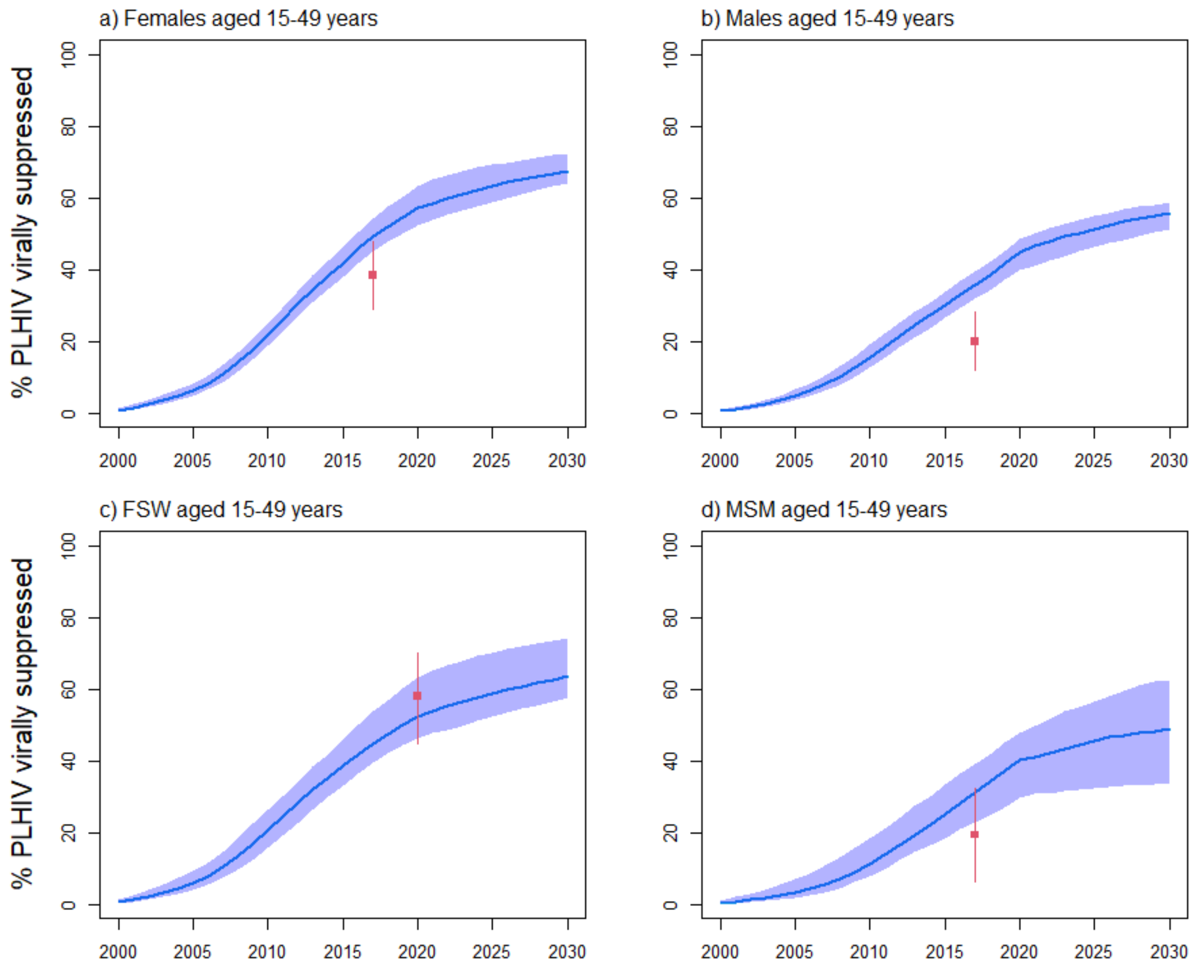

Figure S2o: Côte d'Ivoire model fitting to HIV viral load suppression coverage among a) all females, b) all males, c) all FSW, and d) all MSM living with HIV aged 15-49 years. Blue curves and shades represent median and 90% UI (5th and 95th percentiles across model fits), whereas red squares and intervals represent empirical estimates from local surveys. Note that low coverages in red in panel b) for the year 2017 could not be reproduced by our model as they don't agree well with UNAIDS estimates of the fraction of males PLHIV which are treated (see Table S2a and Figure S2m).

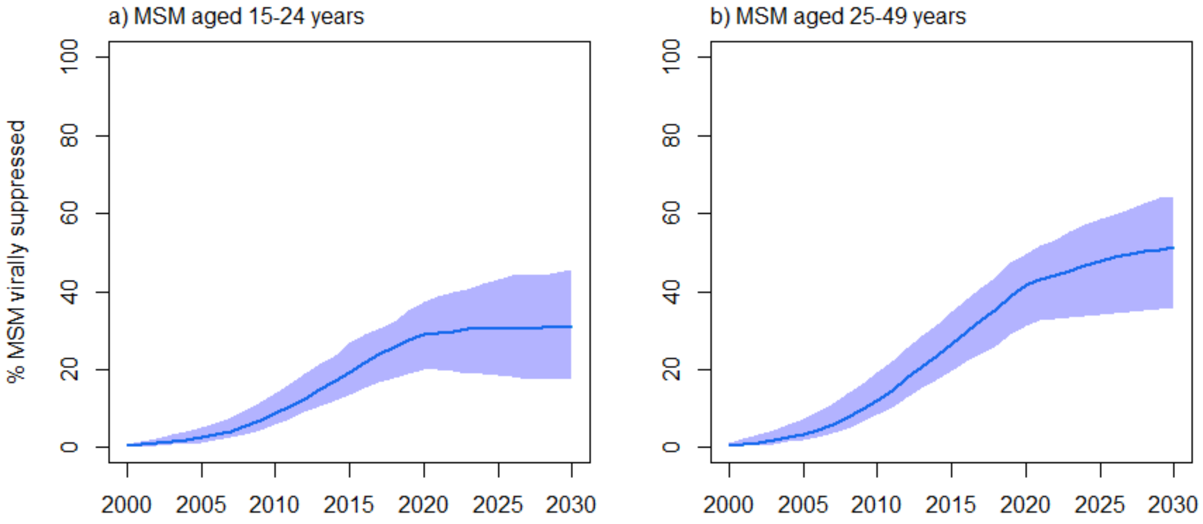

Figure S2p: Côte d'Ivoire model fitting to HIV viral load suppression coverage among MSM aged a) 15-24 years and b) 25-49 years living with HIV. Blue curves and shades represent median and 90% UI (5th and 95th percentiles across model fits). No data was available.

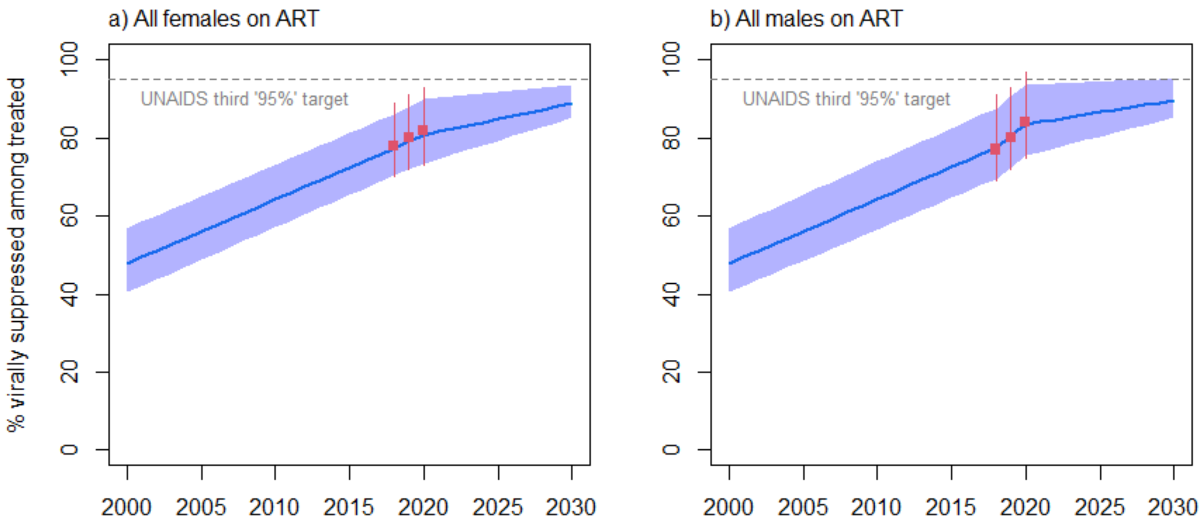

Figure S2q: Côte d'Ivoire model fitting to fractions of a) females and b) males PLHIV on ART which are virally suppressed (the third UNAIDSs "95%" indicator) over time. Blue curves and shades represent median and 90% UI (5th and 95th percentiles across model fits), whereas red squares and intervals represent estimates from UNAIDS used as parameters and not at fitting targets. The grey dashed line corresponds to the UNAIDS's third "95%" target whereby 95% of PLHIV on ART should be virally suppressed in 2025.

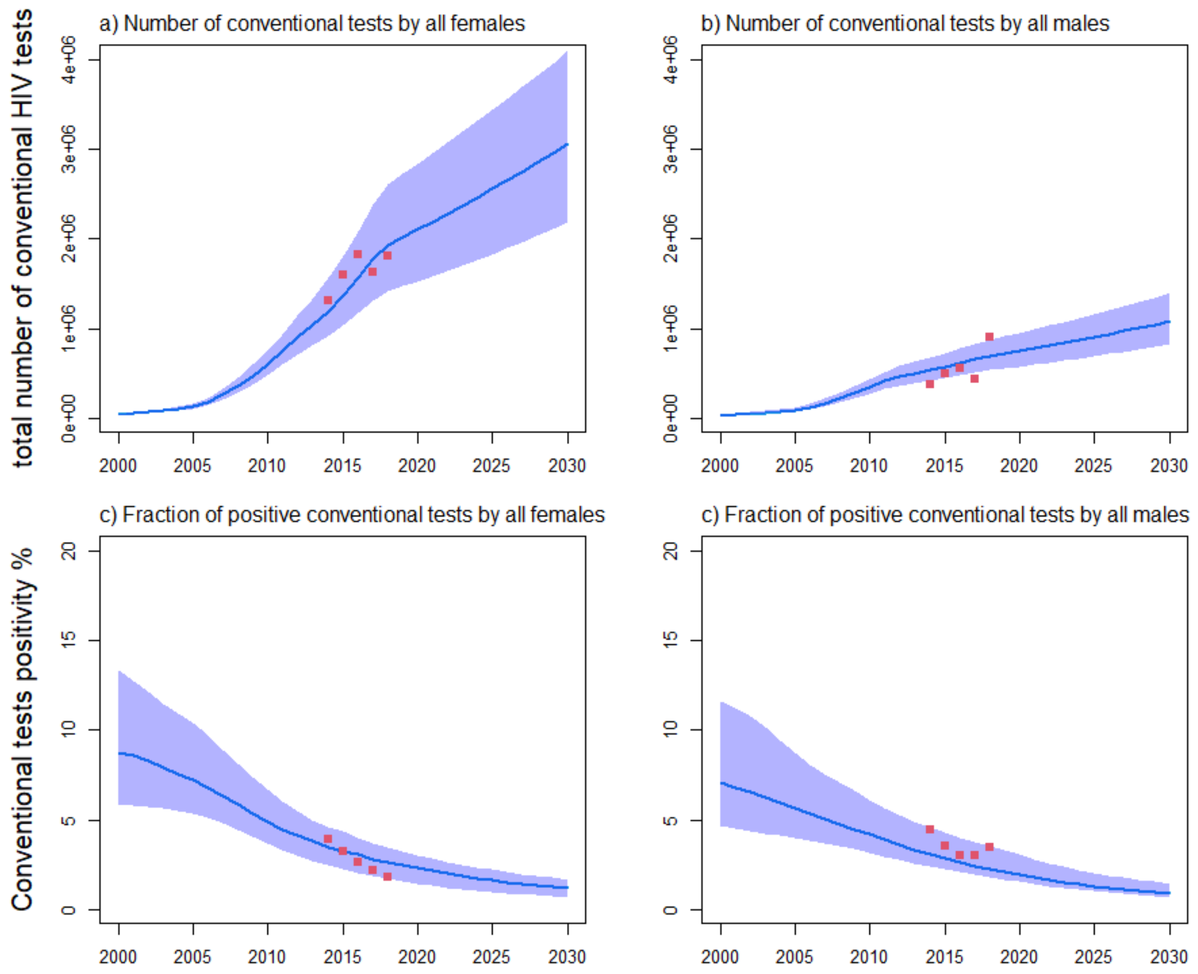

Figure S2r: Côte d'Ivoire model fitting to programmatic data[67] on the total number of conventional tests among a) all females and b) all males. Proportions of positive conventional tests among c) all females, and d) all males. Blue curves and shades represent median and 90% UI (5th and 95th percentiles across model fits), whereas red squares represent programmatic data communicated by UNAIDS.

Results: model fits (Mali)

Demography

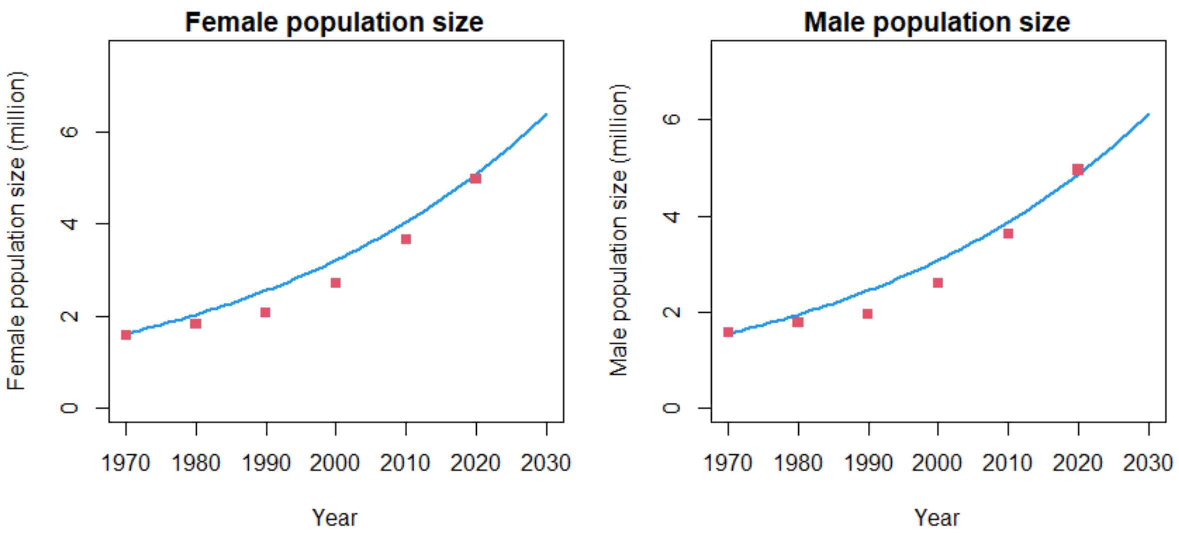

Figure S3a: Mali model fitting to the size of the female and male population aged 15-59 years old over time. Blue curves represent model estimates and red squares the estimates from UNPD[5].

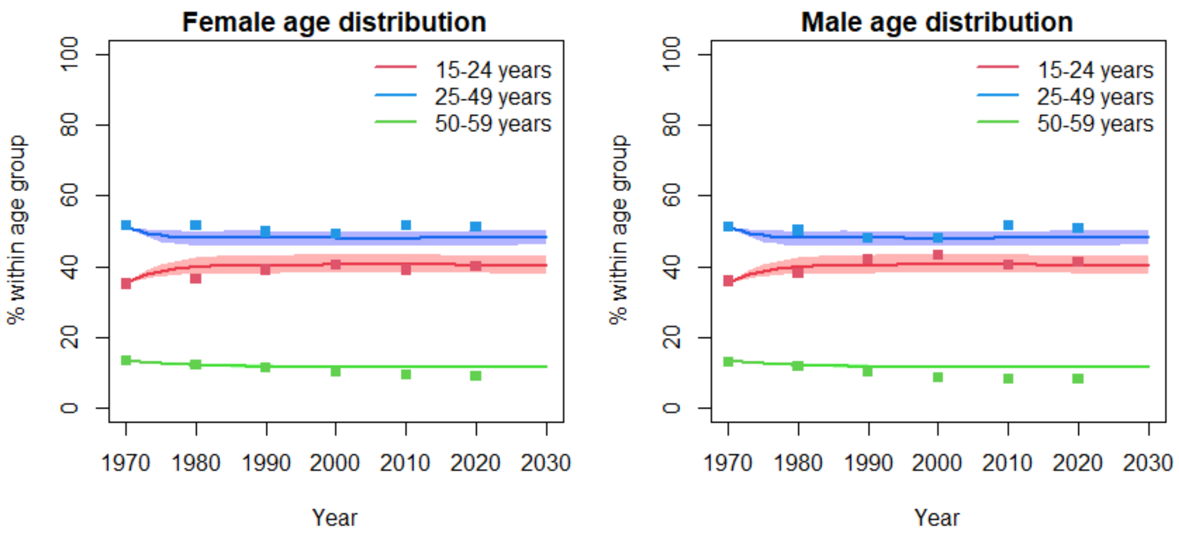

Figure S3b: Mali model fitting to the size of the female and male population aged 15-59 years old over time. Blue curves represent model estimates and red squares the estimates from UNPD[5]. The latter were used to initialise the population age distribution in 1970 (combining males and females), and to calibrate the model to sex-specific estimates for 2020.

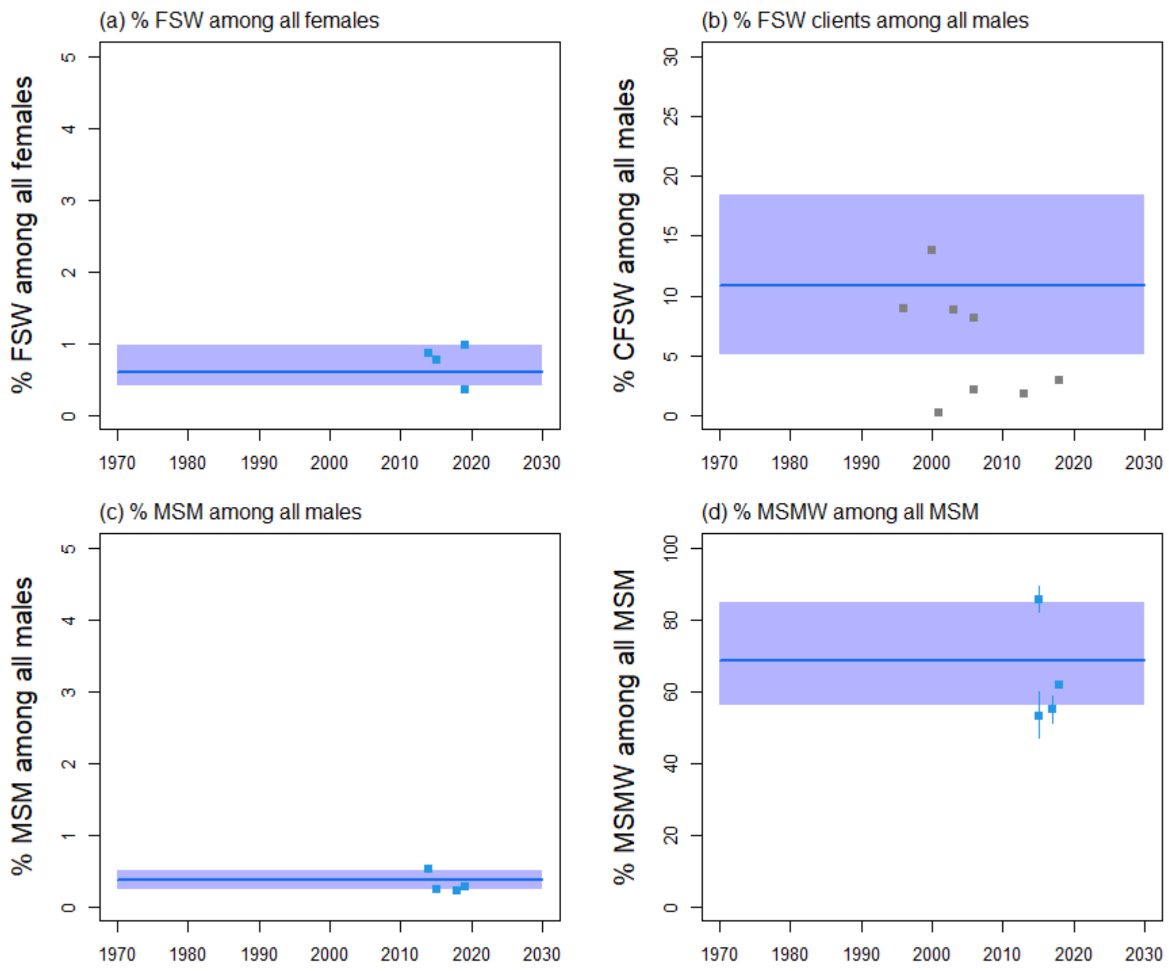

Figure S3c: Mali model fitting to the size of key populations and their clients, with fractions of a) FSW among all females aged 15-59 years, b) FSW clients among all males aged 15-59 years, c) MSM among all males aged 15-59 years, and d) MSMW (men who have sex with men and women) among all MSM aged 15-59 years old over time. Blue curves and shades represent median and 90% UI (5thth and 95th percentiles across model fits), whereas squares and intervals represent empirical estimates (with 95% CI). Grey estimates in panel b) were reported from household surveys and studies among truck drivers, and were only used for comparison as they were deemed to be unrepresentative, The FSW clients population size in the model was calculated using the multiplier method as in [1,34,144,145].

HIV epidemiology

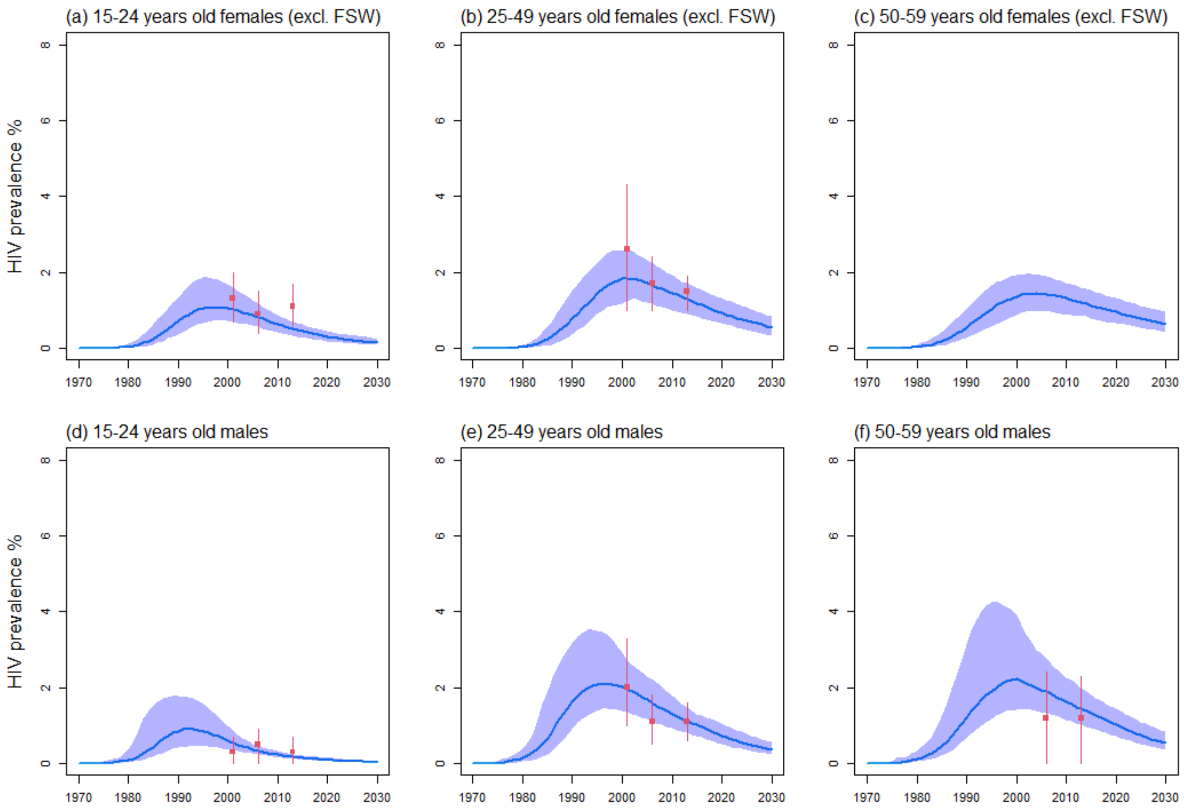

Figure S3d: Mali model fitting to the HIV prevalence among all females aged a) 15-24, b) 25-49, and c) 50-59 years old (excluding FSW), as well as all males aged d) 15-24, e) 25-49 years, and f) 50-59 years old. Blue curves and shades represent median and 90% UI (5th and 95th percentiles across model fits), red squares and intervals represent empirical estimates used for model fitting (with 95% CI).

.

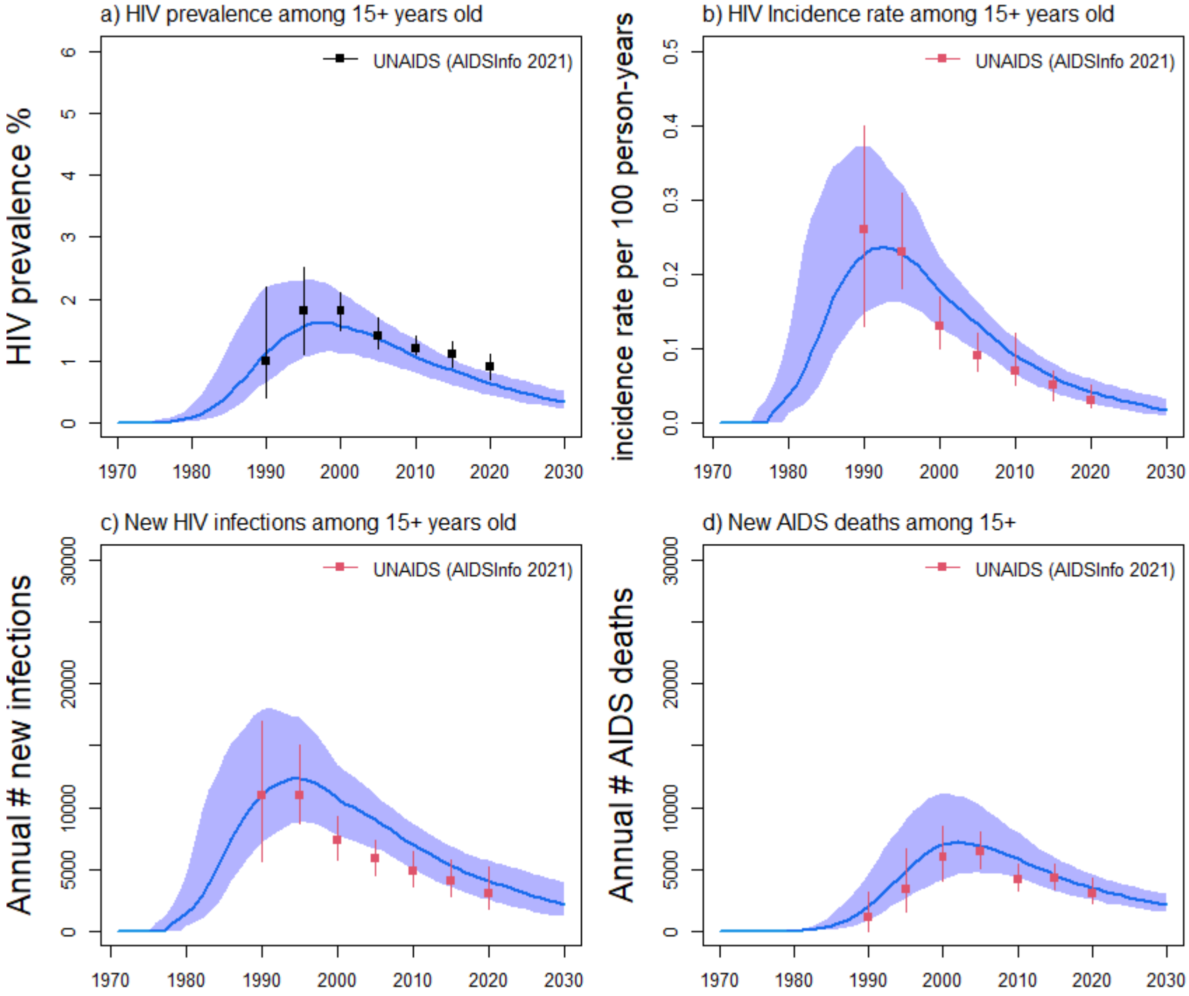

Figure S3e: Estimates of HIV prevalence among a) all adults aged over 15 years old, fits to b) HIV incidence rate, c) annual number of new HIV infections and d) annual HIV-related deaths in Mali from UNAIDS (from the Spectrum/EPP model[146]). Blue curves and shades represent median and 90% UI (5th and 95th percentiles across model fits), red squares and intervals represent empirical estimates used for model fitting, whereas the dark squares and intervals in panel a) represent estimates from UNAIDS (also from the Spectrum/EPP model) only used for comparison.

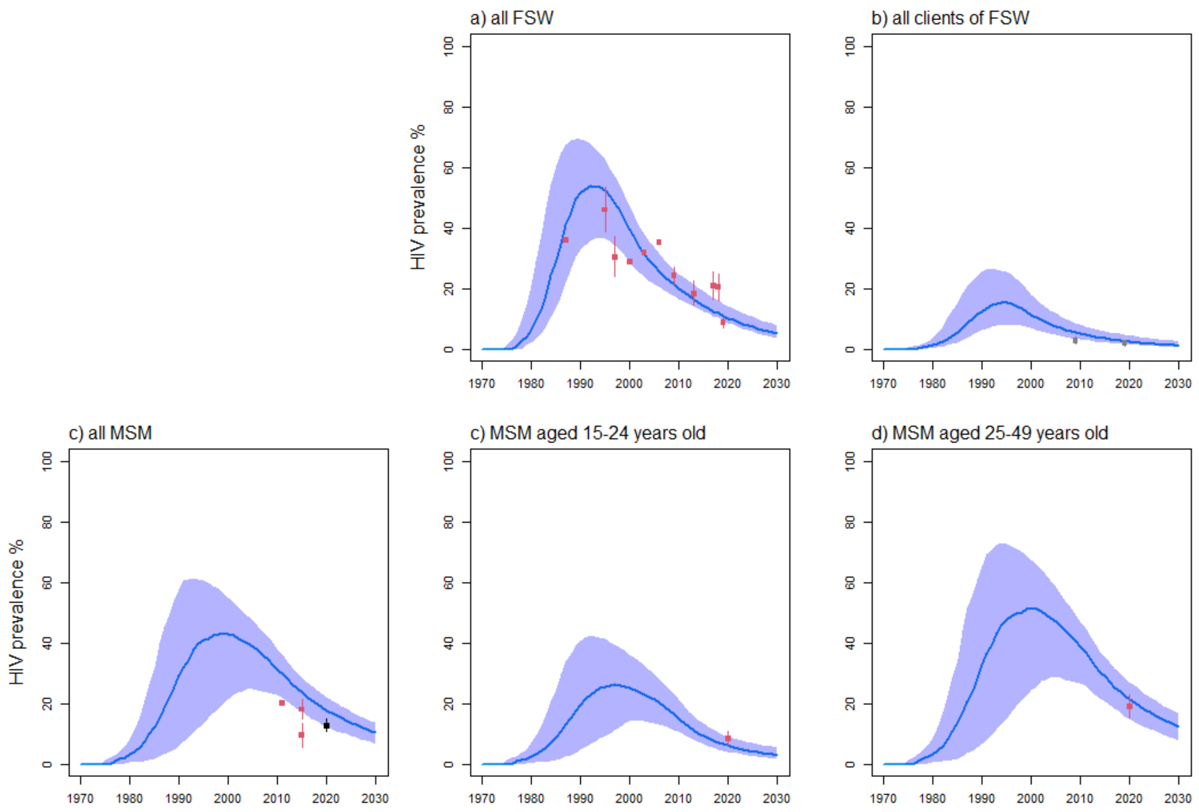

Figure S3f: Mali model fitting to HIV prevalence estimates among all a) FSW, b) clients of FSW, c) MSM, as well as c) MSM aged 15-24 years old, and d) aged 25-49 years old. Blue curves and shades represent median and 90% UI (5th and 95th percentiles across model fits), red squares and intervals represent empirical estimates used for model fitting, whereas the dark square on panel c) represents the estimate fitted by age.

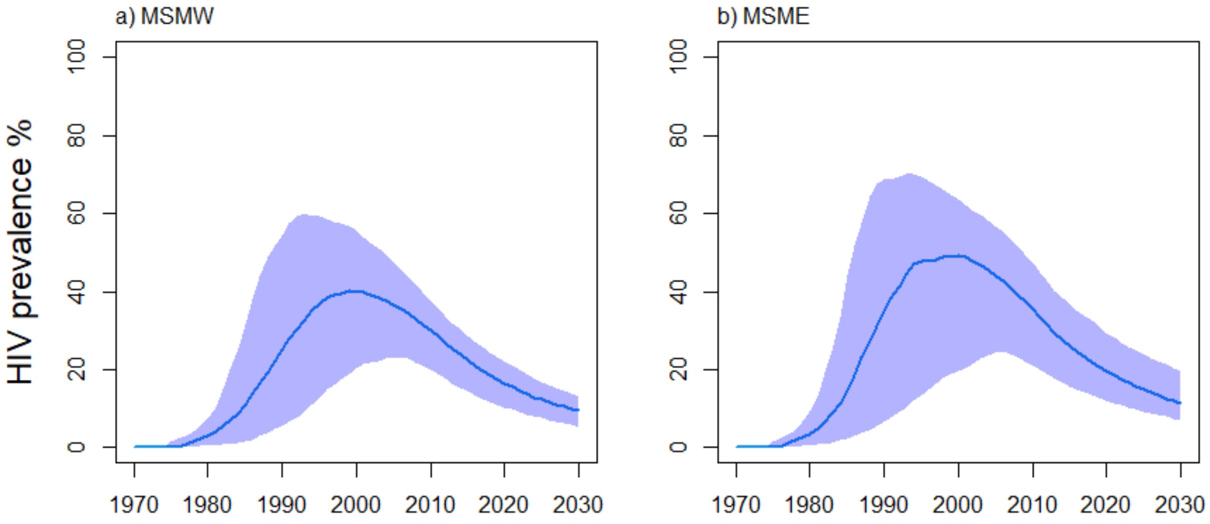

Figure S3g: Mali model estimates of the HIV prevalence among all a) MSMW (men having sex with both men and women) and b) MSME (men having sex with another men) MSM. Blue curves and shades represent median and 90% UI (5th and 95th percentiles across model fits). No data was available.

HIV treatment cascade

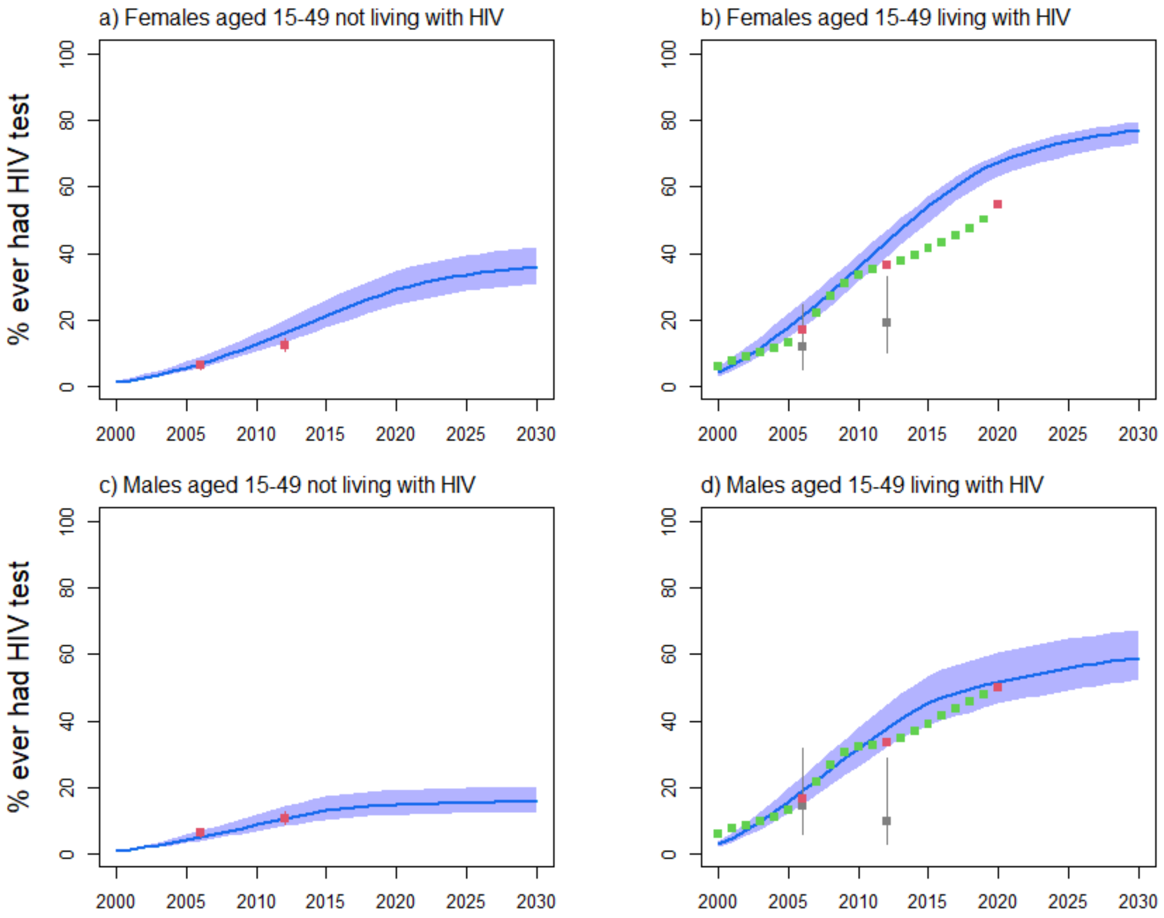

Figure S3h: Mali model fitting to the fraction ever having tested for HIV among all females aged 15-49 years old a) not living with HIV, b) living with HIV, and males aged 15-49 years old c) not living with HIV, d) living with HIV. Blue curves and shades represent median and 90% UI (5th and 95th percentiles across model fits), red squares and intervals represent estimates from UNAIDS Shiny90 [126] used for model fitting (green=Shiny90 estimates used for comparison), whereas grey squares represent estimates from DHS surveys among PLHIV which were used for comparison.

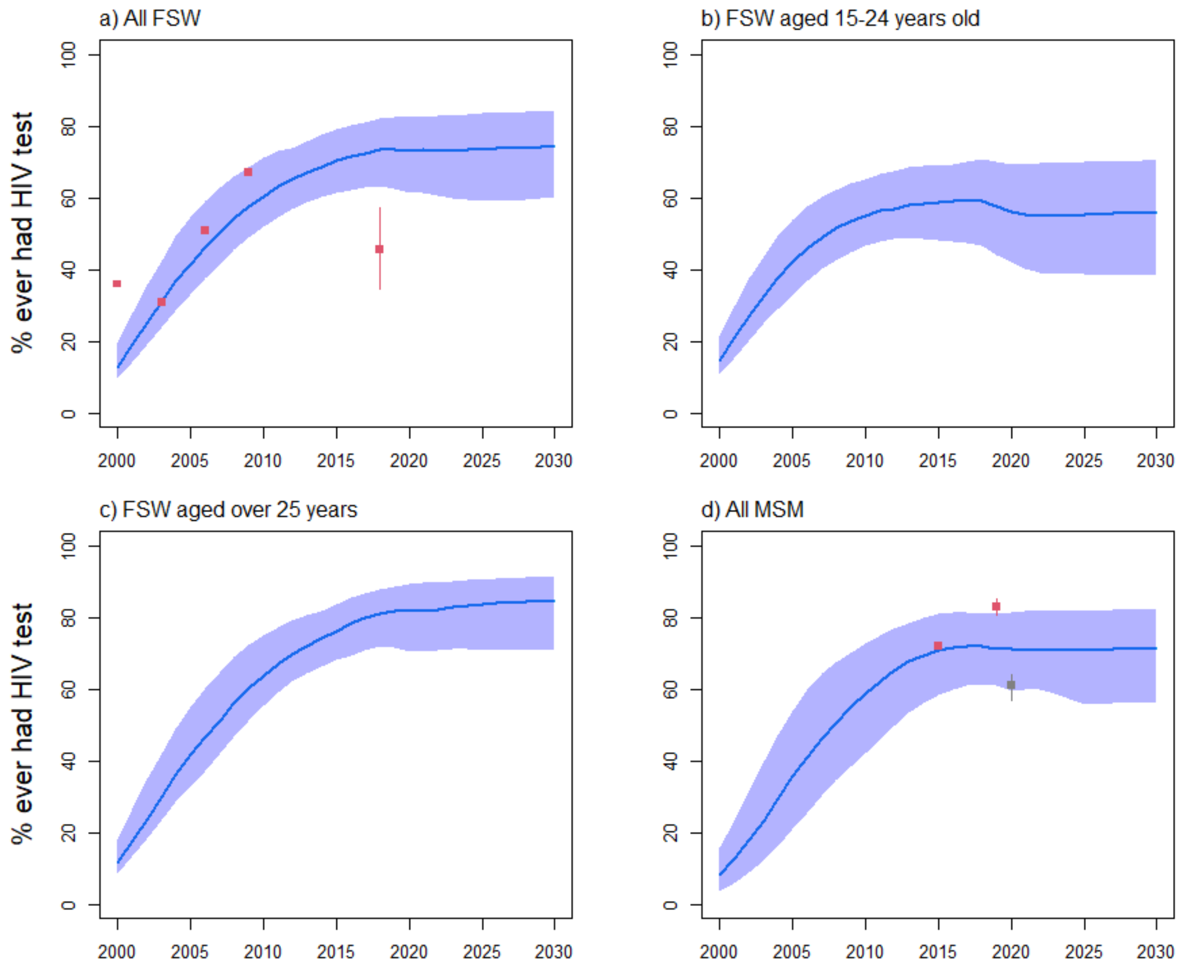

Figure S3i: Mali model fitting to the fraction ever having tested for HIV among a) all FSW, b) FSW aged 15-24 years, c) FSW aged 25-49 years, and d) MSM. Blue curves and shades represent median and 90% UI (5th and 95th percentiles across model fits), red squares and intervals represent empirical estimates used for model fitting, the grey square for 2020 in panel d) was an estimate of the fraction of MSM having had an HIV test in the last year, which was used for comparison. We anticipated the estimate from Tounkara et al. for the year 2018[103] in panel a) to be lower than expected, as it was calculated among FSW living with HIV only and because all the women reporting being newly diagnosed during the study reported never having tested for HIV in the past.

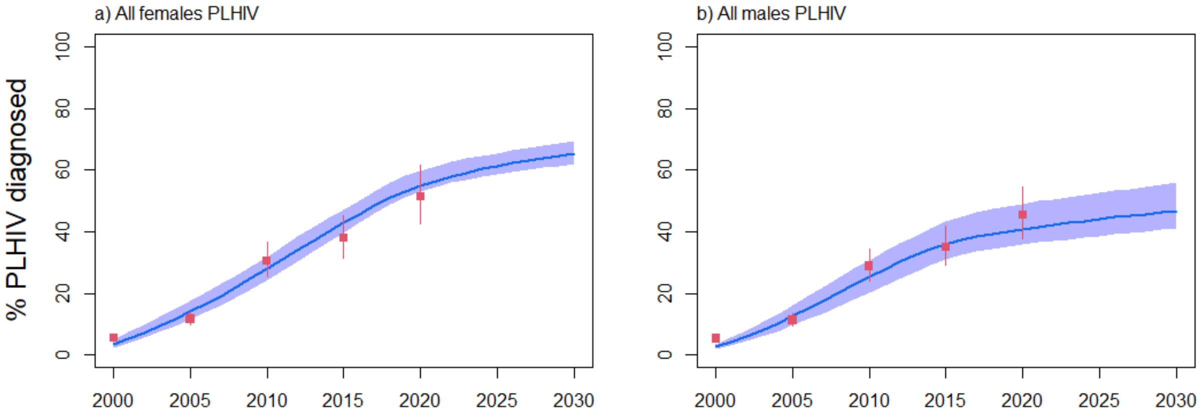

Figure S3j: Mali model fitting to the fraction of a) all females living with HIV and b) all males living with HIV being diagnosed. Blue curves and shades represent median and 90% UI (5th and 95th percentiles across model fits), red squares and intervals represent estimates from UNAIDS Shiny90 used for model fitting.

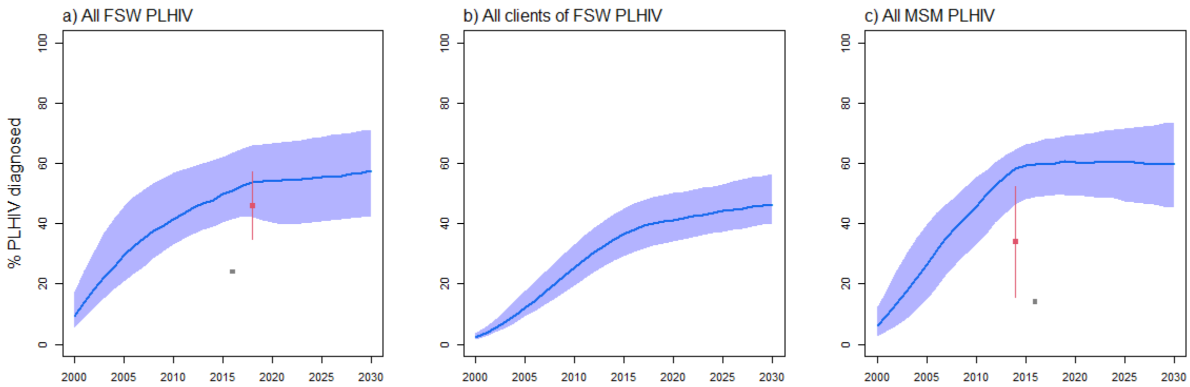

Figure S3k: Mali model fitting to the fraction of a) all FSW living with HIV, b) all male clients of FSW living with HIV, and c) all MSM living with HIV diagnosed. Blue curves and shades represent median and 90% UI (5th and 95th percentiles across model fits), red squares and intervals represent estimates from empirical surveys[48,103], whereas grey squares represent national estimates for which no report or underlying study could be identified, and which were only used for comparison. Empirical estimates are self-reported and likely to be under-estimates.[147]. In particular, the estimate from Hakim et al. [48] in panel c) could not be well fitted as it did not agree well with data on HIV viral suppression among MSM in the country(see Figures S3n and S3o).

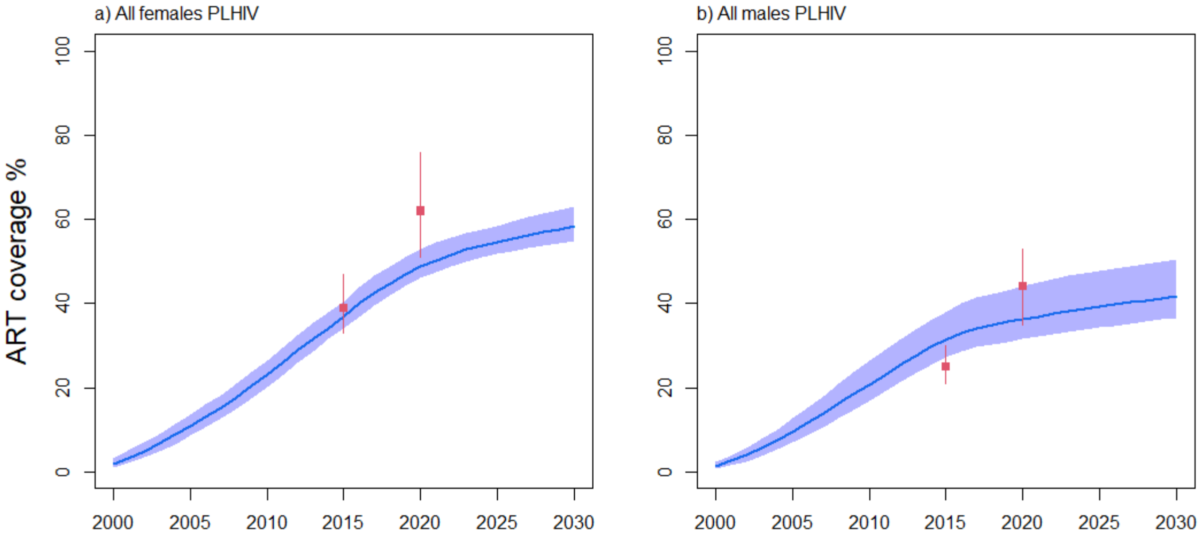

Figure S3l: Mali model fitting to ART coverage among a) all females and b) all males living with HIV. Blue curves and shades represent median and 90% UI (5th and 95th percentiles across model fits), whereas red squares and intervals represent estimates from UNAIDS (with 95%CI), using the Spectrum/EPP model.

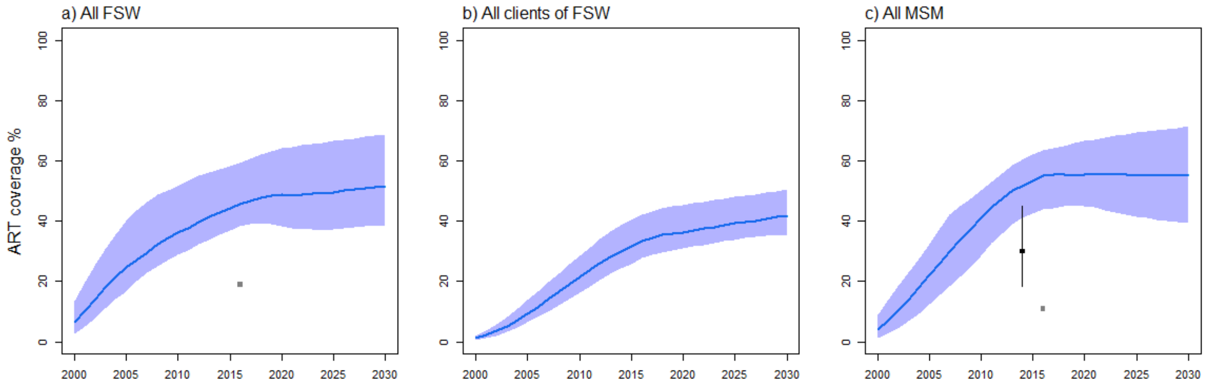

Figure S3m: Mali model fitting to ART coverage among a) all FSW, b) all male clients of FSW, and c) all MSM living with HIV. Blue curves and shades represent median and 90% UI (5th and 95th percentiles across model fits), whereas red squares and intervals represent empirical estimates from local surveys. Dark point and interval in panel c) represent self-reported use of ART, and all study participants reporting being on ART were virally suppressed (which was fitted by age in our model, see figure S3o). Grey squares in panels a) and c) represent estimates for which no report or underlying study could be identified. Although no ART coverage data was used, we fitted the Mali model to estimates of viral suppression in the country.

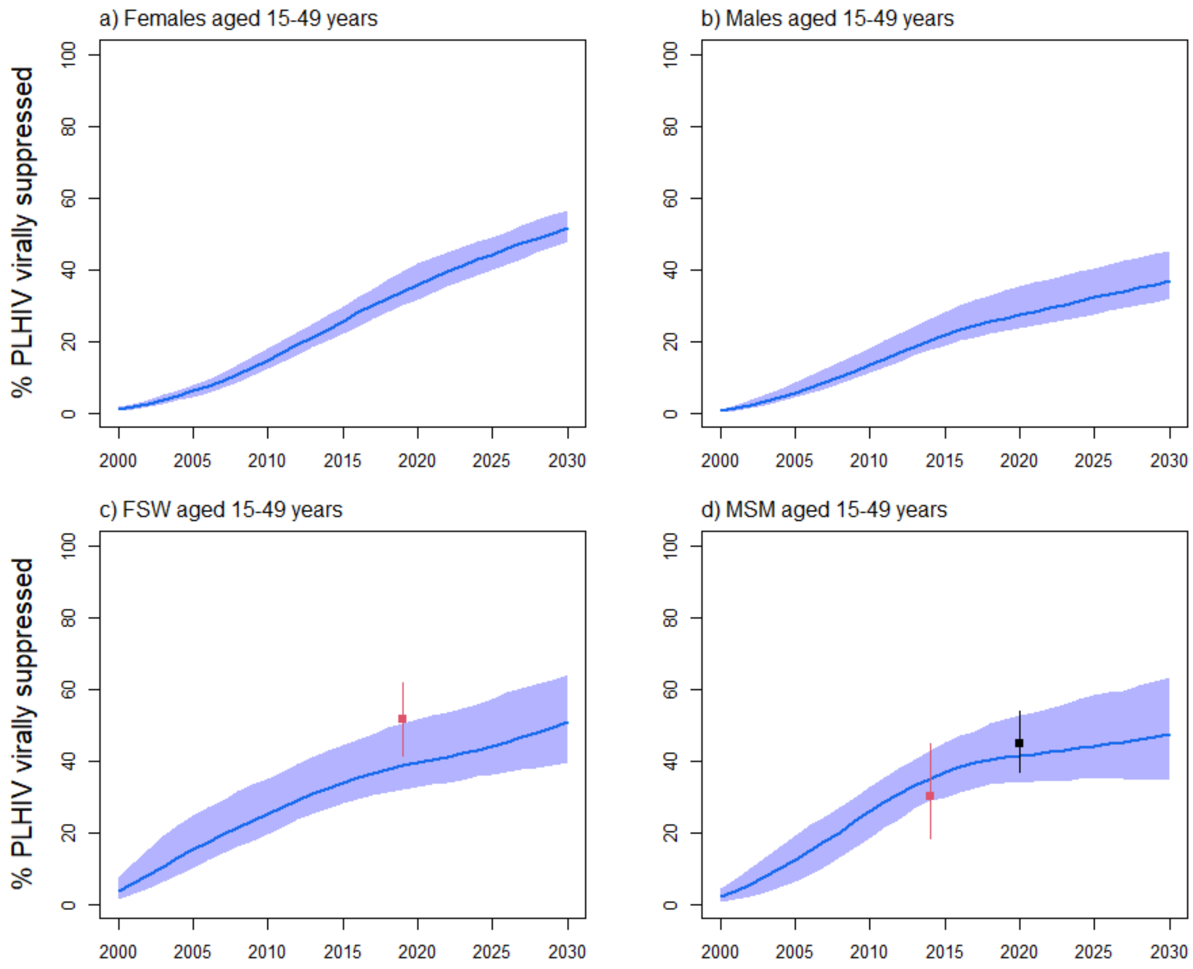

Figure S3n: Mali model fitting to HIV viral load suppression coverage among a) all females, b) all males, c) all FSW, and d) all MSM aged 15-49 years living with HIV. Blue curves and shades represent median and 90% UI (5th and 95th percentiles across model fits), whereas red squares and intervals represent empirical estimates from local surveys. The black square in panel d) was only used for comparison; it was aggregated from the age-stratified 2020 estimates used at the fitting stage (see figure S3o).

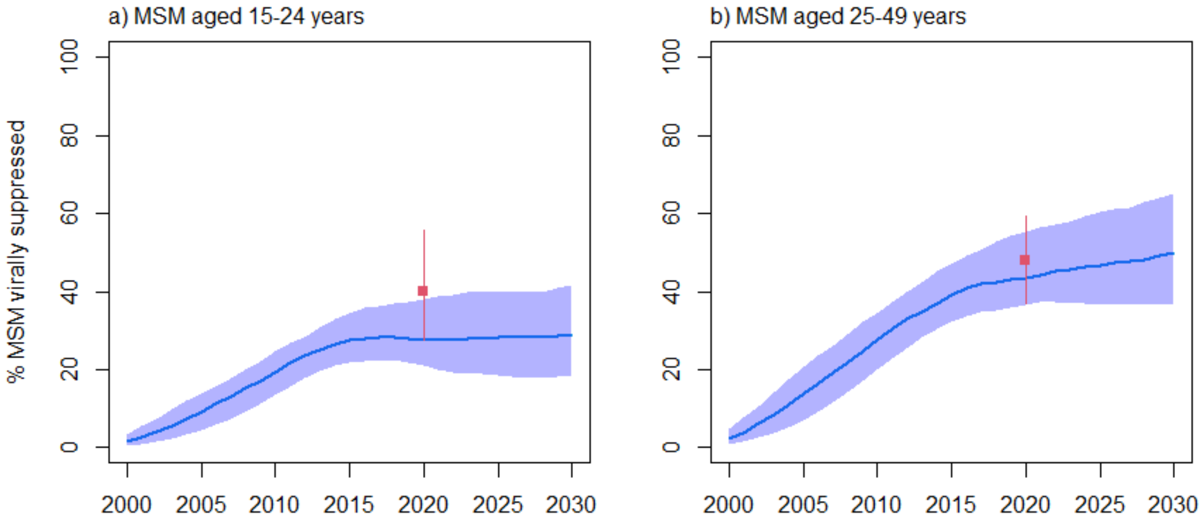

Figure S3o: Mali model fitting to HIV viral load suppression coverage among MSM living with HIV aged a) 15-24 years and b) 25-49 years. Blue curves and shades represent median and 90% UI (5th and 95th percentiles across model fits), whereas red squares and intervals represent empirical estimates.

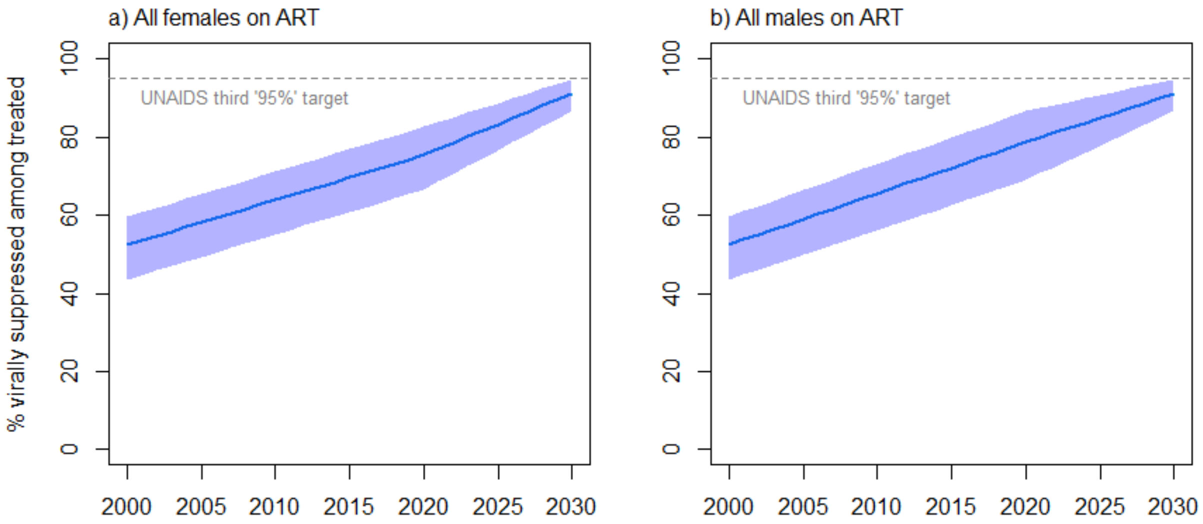

Figure S3p: Mali model estimates of the fraction of a) females and b) males living with HIV on ART which are virally suppressed (the third UNAIDSs "95%" indicator) over time, which is used as a parameter in our model. There were no available estimates of this fraction, and the plausible fractions were estimated by using the relationship between the 1st and 3rd "95%" indicators in Côte d'Ivoire and Senegal and applying this relationship to the estimate of the 1st "95%" indicator in Mali. The grey dashed line corresponds to the UNAIDS's third "95%" target whereby 95% of PLHIV on ART should be virally suppressed in 2025.

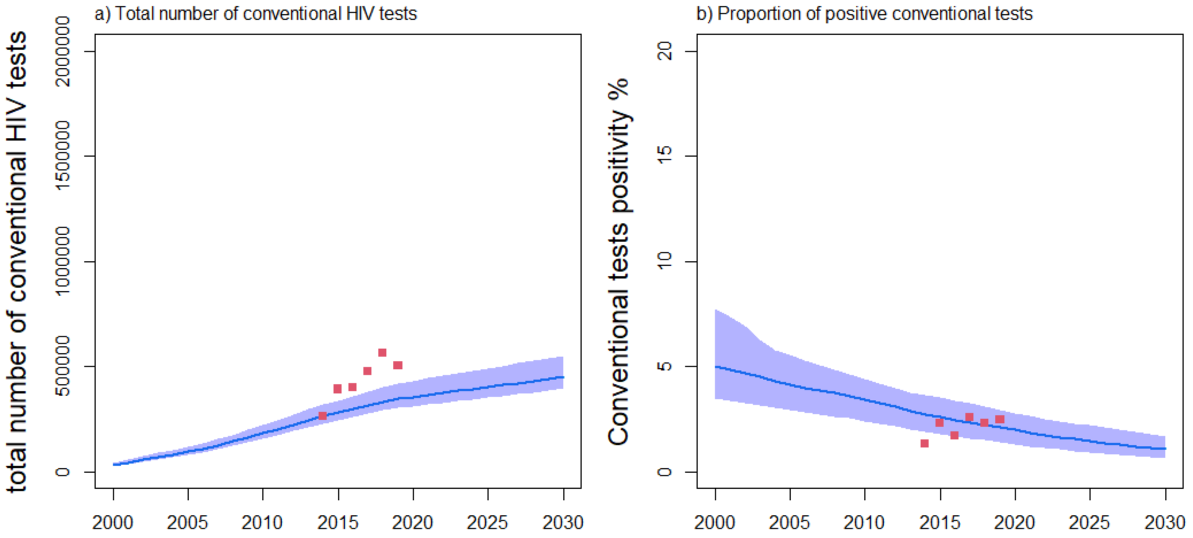

Figure S3q: Mali model fitting to programmatic data[67] on a) the total number of conventional tests and b) proportion of these tests which were positive. Blue curves and shades represent median and 90%UI (5th and 95th percentiles across model fits), whereas red squares represent programmatic data from UNAIDS[67].

Results: model fits (Senegal)

Demography

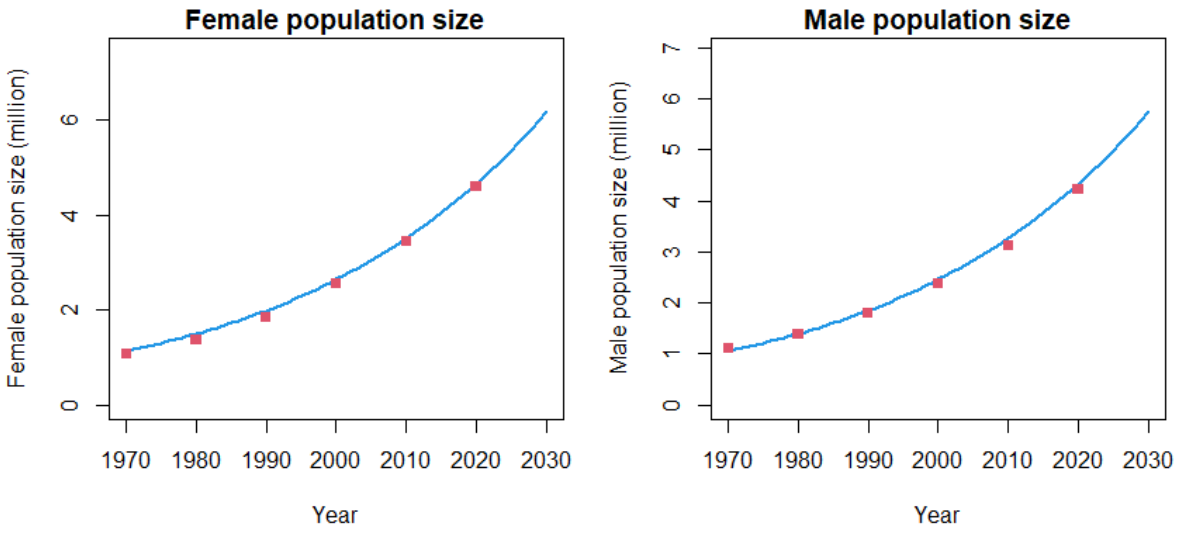

Figure S4a: Senegal model fitting to the size of the (left) female and (right) male populations aged 15-59 years old over time. Blue curves represent model estimates, while red squares show estimates from UNPD.

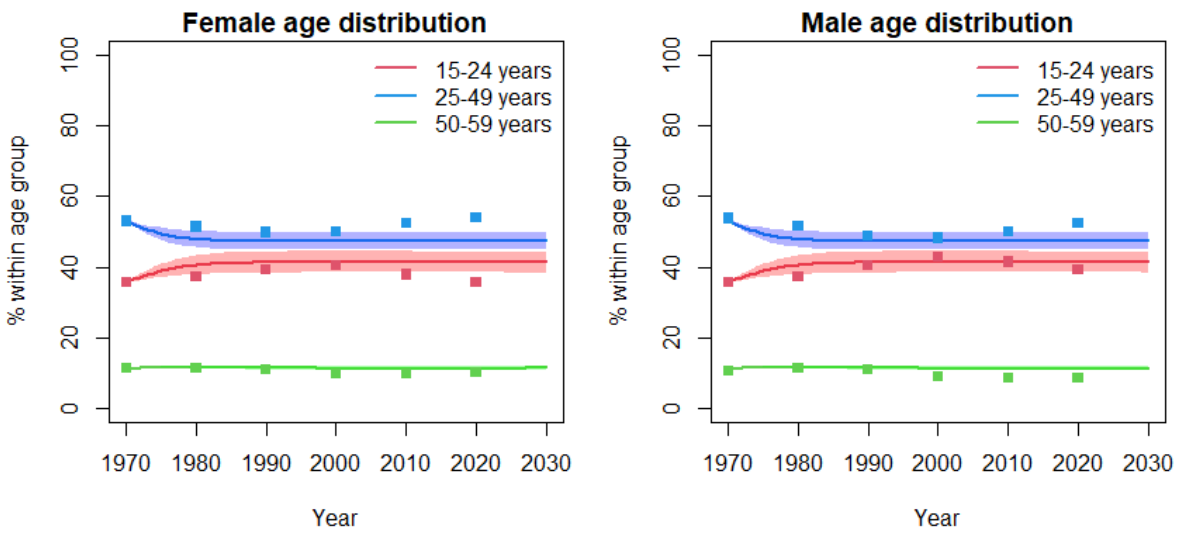

Figure S4b: Senegal model fitting to the age distribution of (left) females and (right) male populations aged 15-59 years old over time. Curves represent model estimates while squares show estimates from UNPD. The latter were used to initialise the population age distribution in 1970 (combining males and females), and to calibrate the model to sex-specific estimates for 2020.

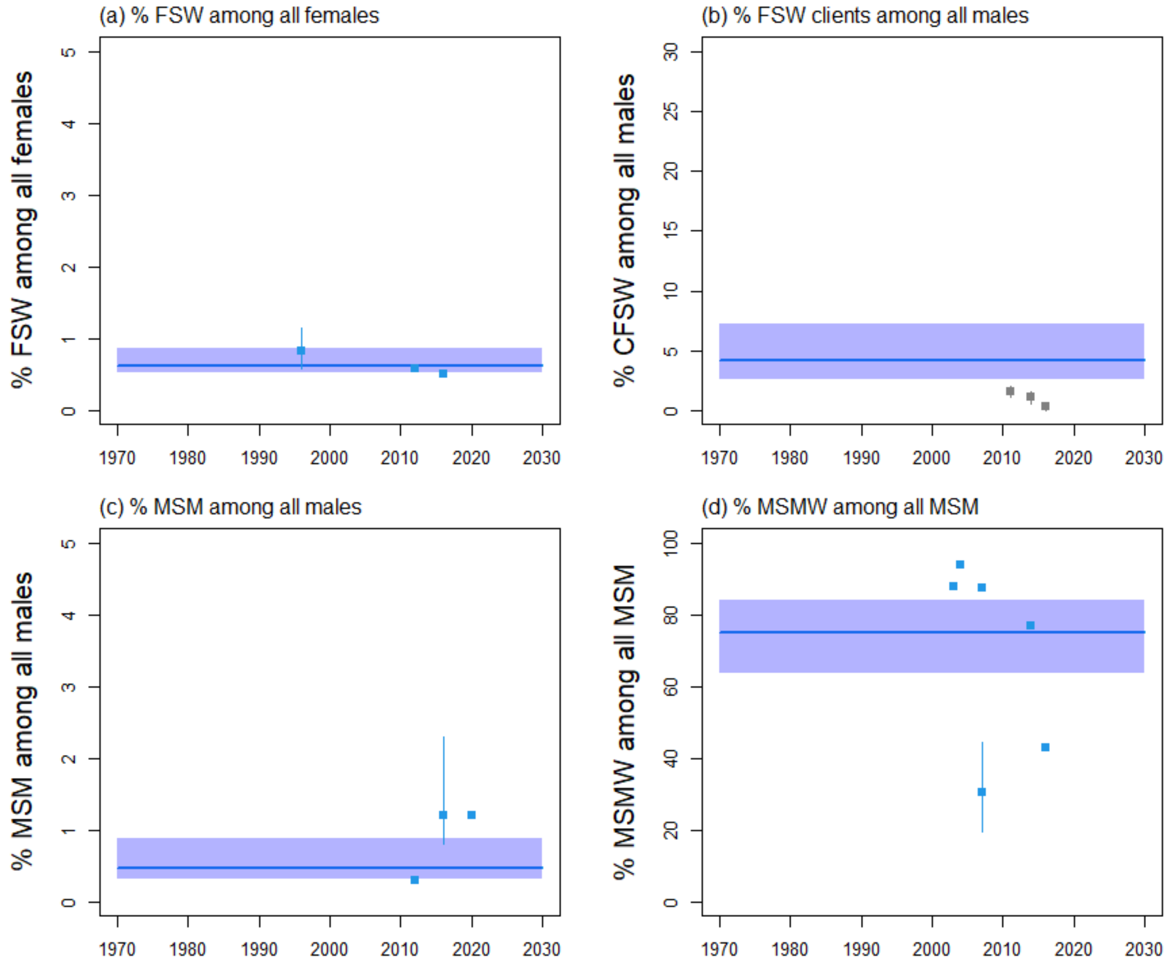

Figure S4c: Senegal model fitting to the size of key populations and their clients, with the fraction of a) FSW among all females aged 15-59 years, b) FSW clients among all males aged 15-59 years, c) MSM among all males aged 15-59 years, and d) MSMW (men who have sex with men and women) among all MSM aged 15-59 years old over time. Blue curves and shades represent median and 90% UI (5thth and 95th percentiles across model fits), whereas squares and intervals represent empirical estimates. Grey estimates in panel b) were reported from household surveys and were only used for comparison as they were deemed to biased to be unrepresentative, The FSW clients population size in the model was calculated using the multiplier method as in [1,34,144,145]. Larger fractions of MSM population in panel c) could not be reproduced by the model due to incompatibilities with 1) concomitant low HIV prevalence among all males and high prevalence among MSM, and 2) concomitant high ART coverage among all males and low ART coverage among MSM.

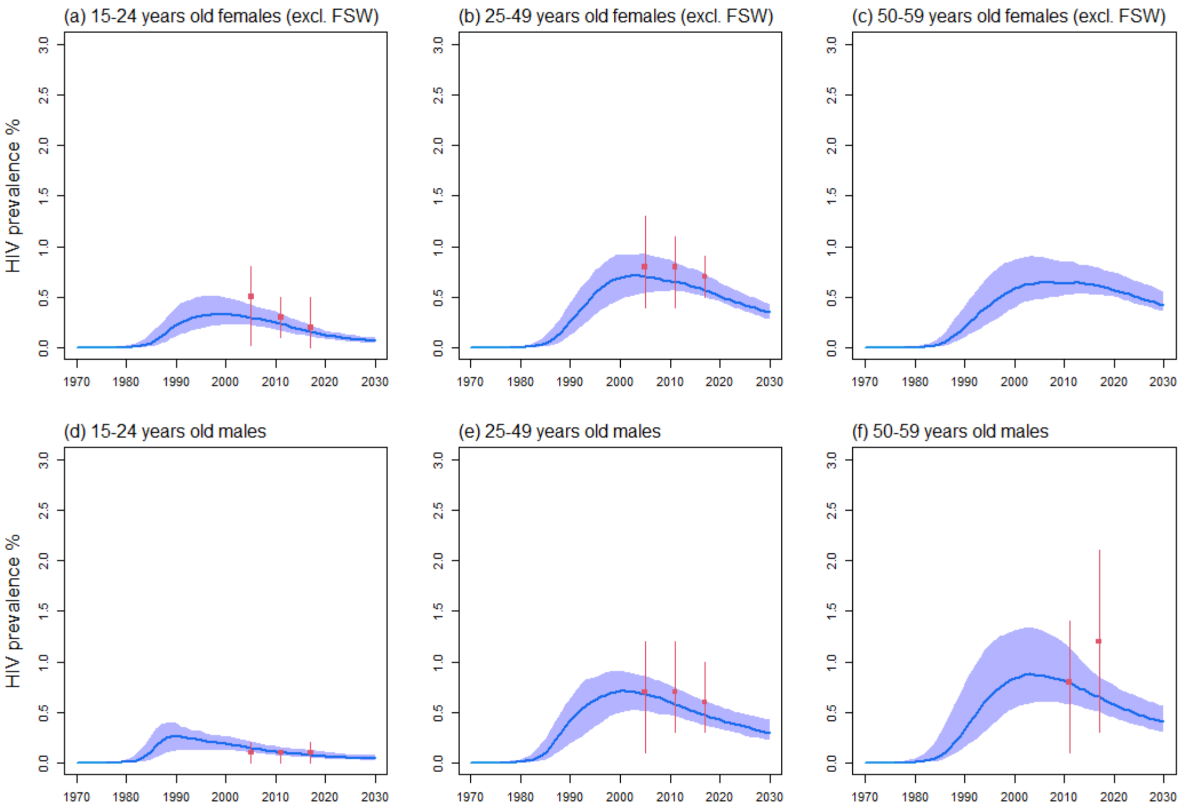

Figure S4d: Senegal model fitting to the HIV prevalence among all females aged a) 15-24, b) 25-49, and c) 50-59 years old (excluding FSW), as well as all males aged d) 15-24, e) 25-49 years, and f) 50-59 years old. Blue curves and shades represent median and 90% UI (5th and 95th percentiles across model fits), whereas red squares and intervals represent empirical estimates used for model fitting.

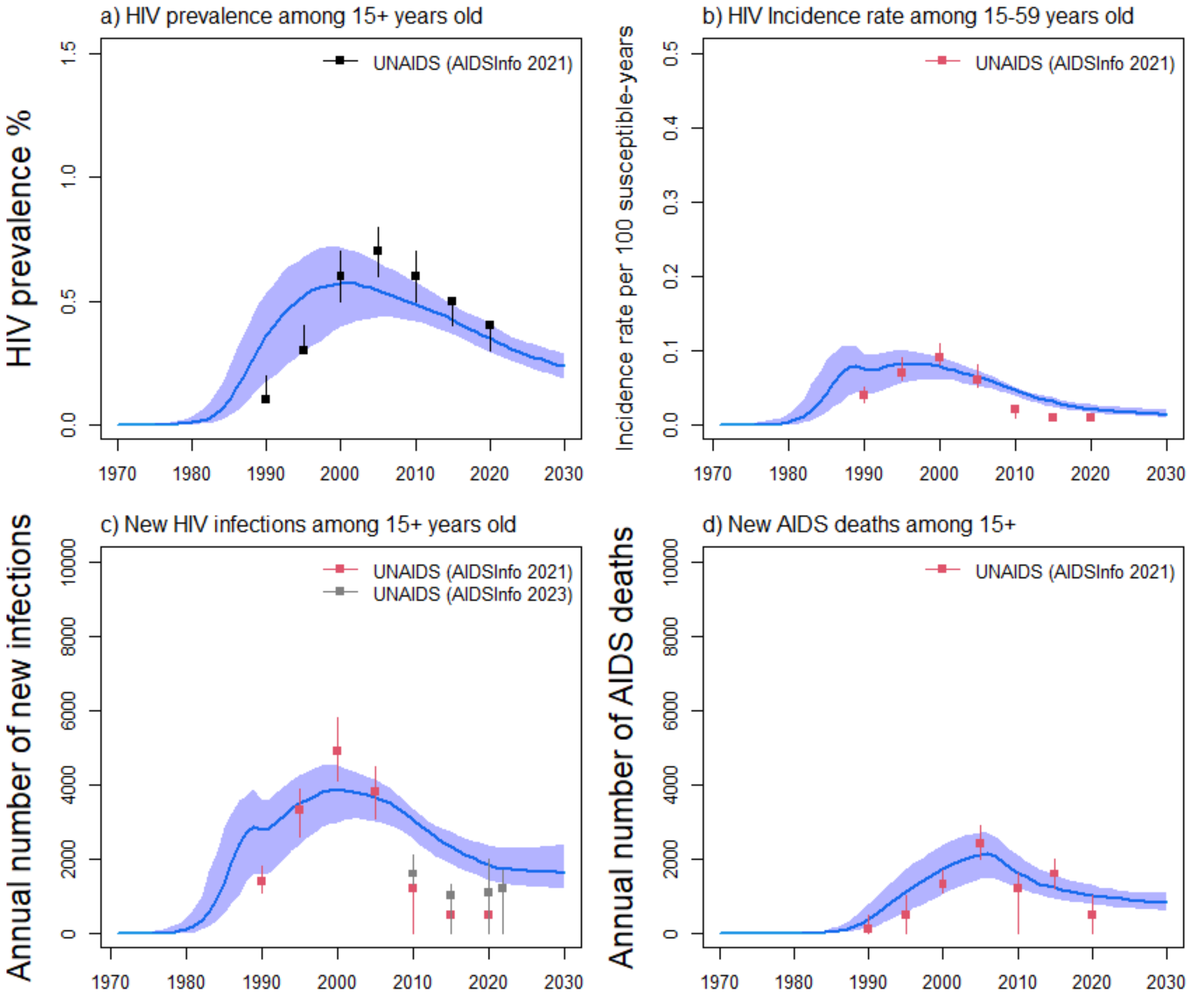

Figure S4e: Estimates of HIV prevalence among a) all adults aged over 15 years old, fits to b) HIV incidence rate, c) annual number of new HIV infections and d) annual HIV-related deaths in Senegal from UNAIDS (from the Spectrum/EPP model[146]). Blue curves and shades represent median and 90% UI (5th and 95th percentiles across model fits), red squares and intervals represent empirical estimates used for model fitting, whereas the dark squares and intervals in panel a) represent estimates from UNAIDS (also from the Spectrum/EPP model) only used for comparison, whereas grey squares and intervals in panel c) represent new estimates from UNAIDS (Spectrum/EPP) which were published in July 2023 and not available at the time of our analysis.

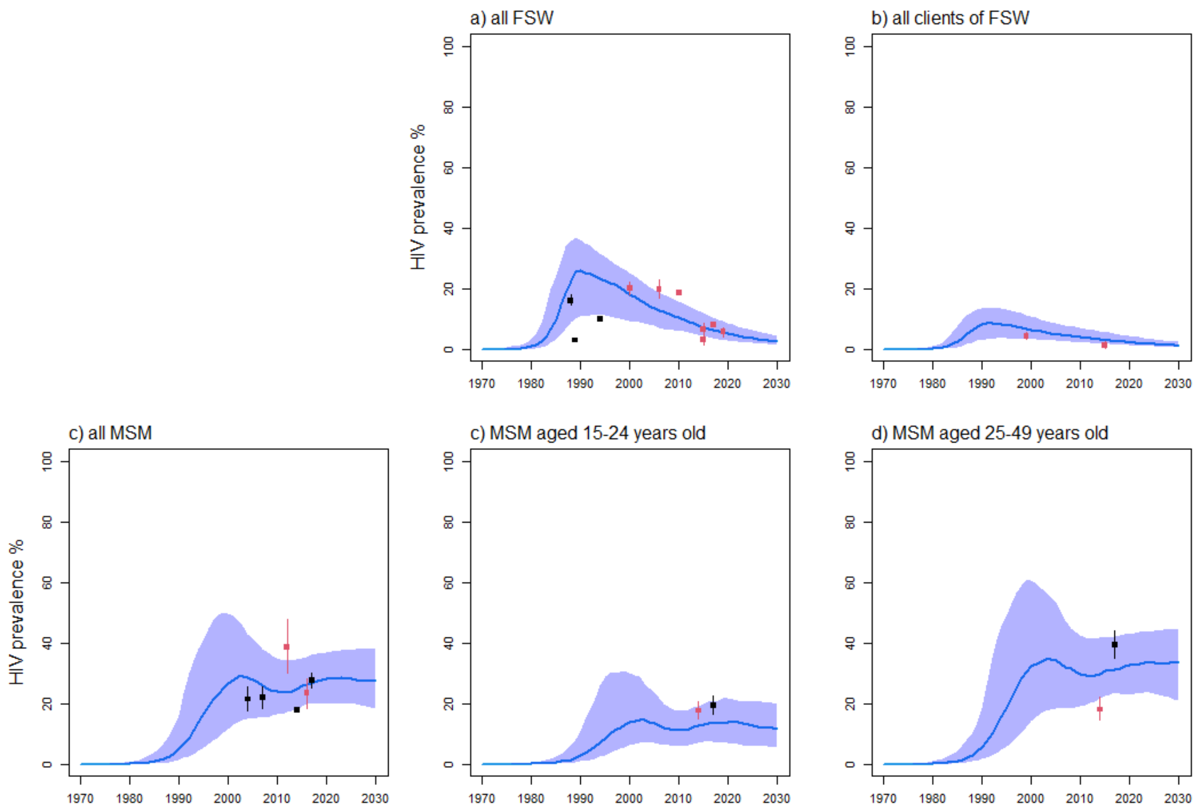

Figure S4f: Senegal model fitting to HIV prevalence estimates among a) all FSW, b) all clients of FSW, c) MSM, as well as c) MSM aged 15-24 years old, and d) aged 25-49 years old. Blue curves and shades represent median and 90% UI (5th and 95th percentiles across model fits), red squares and intervals represent empirical estimates used for model fitting. Dark squares for FSW correspond to estimates which could not be sourced to a particular study or report and were only used for comparison, whereas those on panel c-d) represent estimates fitted by age and for bisexuals/exclusive MSM separately.

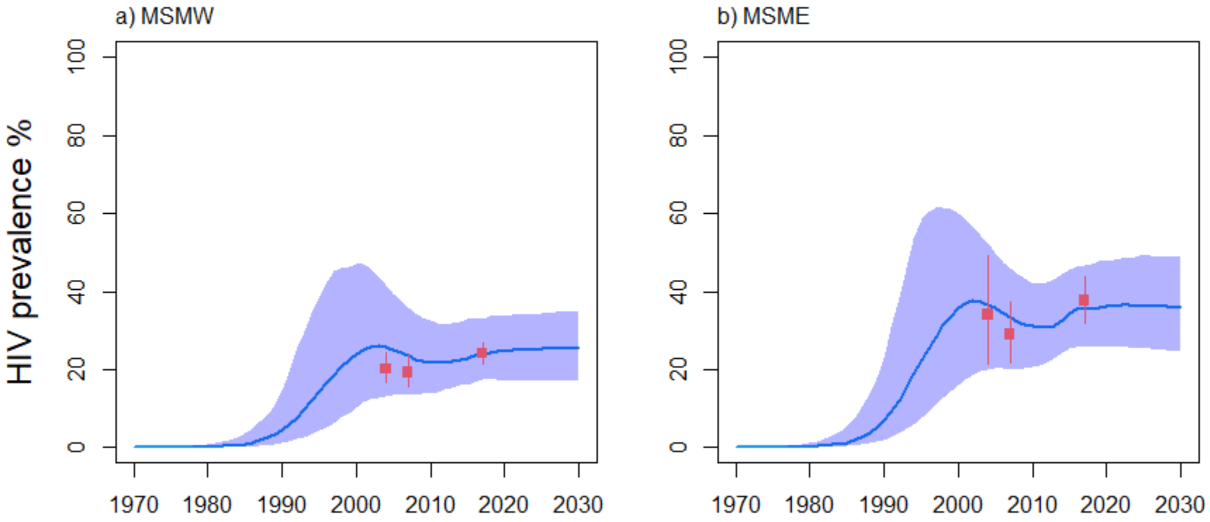

Figure S4g: Senegal model fitting to HIV prevalence estimates among all a) MSMW (men having sex with both men and women) and b) MSME (men having sex with other men exclusively) MSM. Blue curves and shades represent median and 90% UI (5th and 95th percentiles across model fits), whereas red squares and intervals represent empirical estimates.

HIV treatment cascade

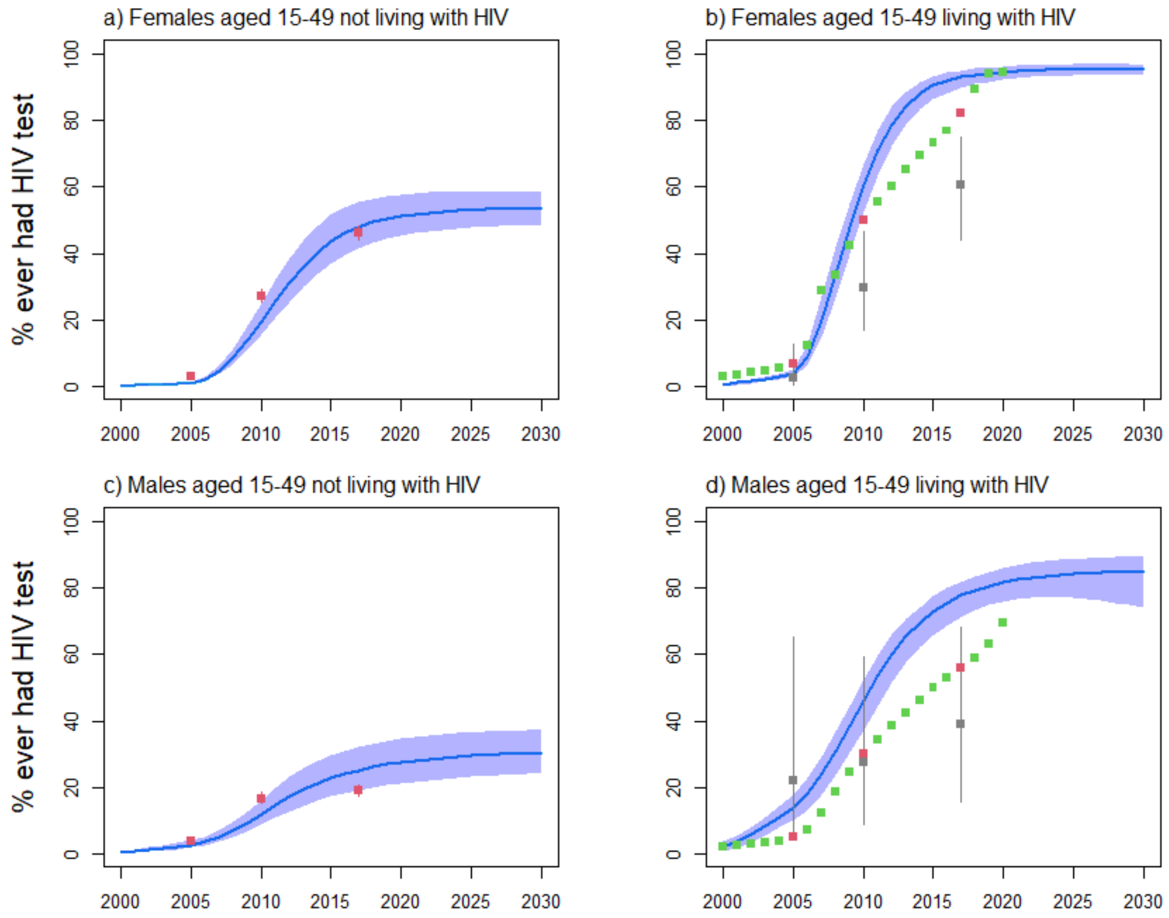

Figure S4h: Senegal model fitting to the fraction ever having tested for HIV among all females aged 15-49 years old a) not living with HIV, and b) living with HIV, and males aged 15-49 years old c) not living with HIV, and d) living with HIV. Blue curves and shades represent median and 90% UI (5th and 95th percentiles across model fits), red squares and intervals represent estimates from UNAIDS Shiny90 used for model fitting (green=those used for comparison), whereas grey squares represent estimates among PLHIV from DHS surveys only used for comparison. Our model estimates among PLHIV (panels b) and d)) were higher than empirical estimates because of high coverage of ART for the recent period being predicted by UNAIDS (see Figure S4l).

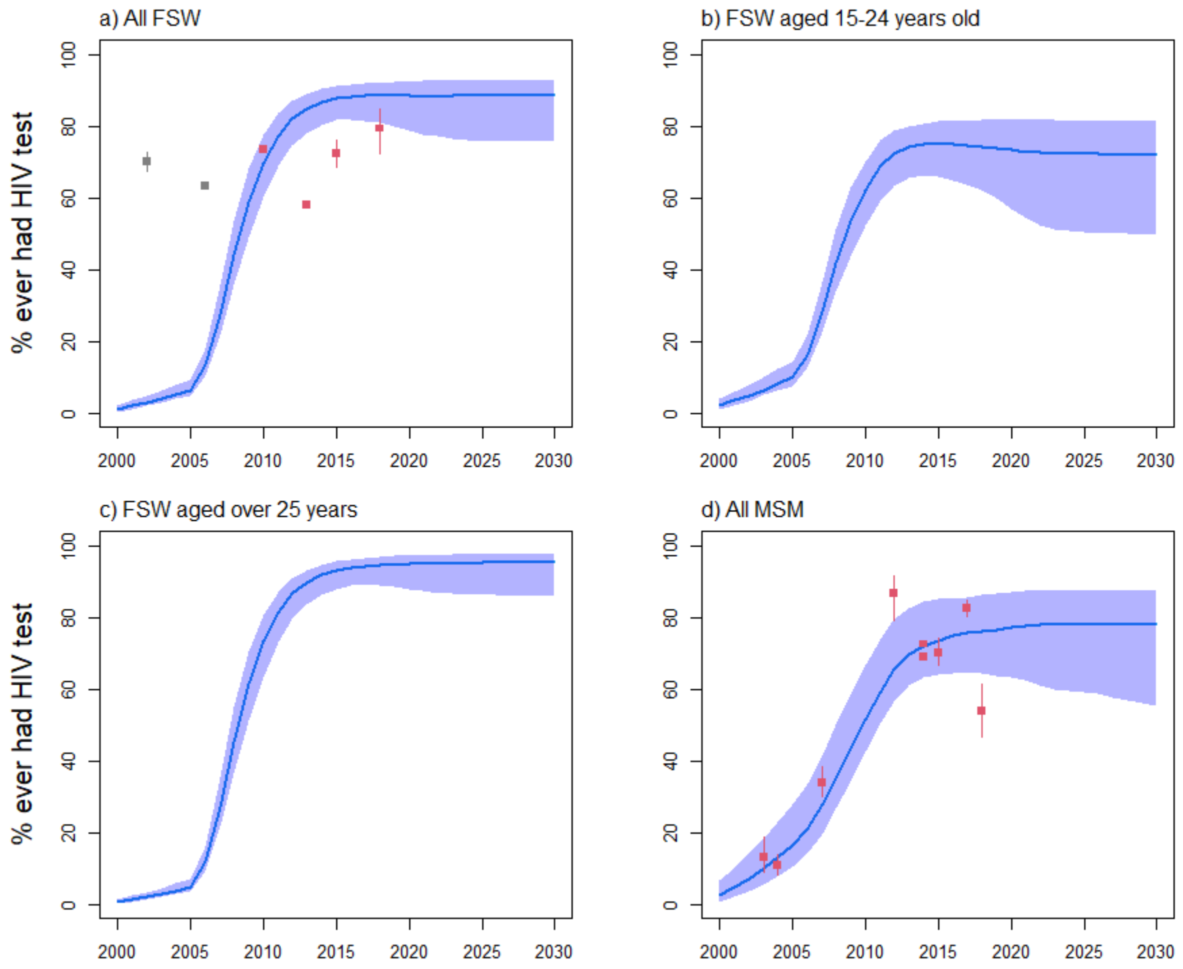

Figure S4i: Senegal model fitting to the fraction ever having tested for HIV among a) all FSW, b) FSW aged 15-24 years, c) FSW aged 25-49 years, and d) all MSM. Blue curves and shades represent median and 90% UI (5th and 95th percentiles across model fits), red squares and intervals represent empirical estimates used for model fitting, whereas the grey squares in panel a) corresponded to studies from STI clinics, which were only used for comparison.

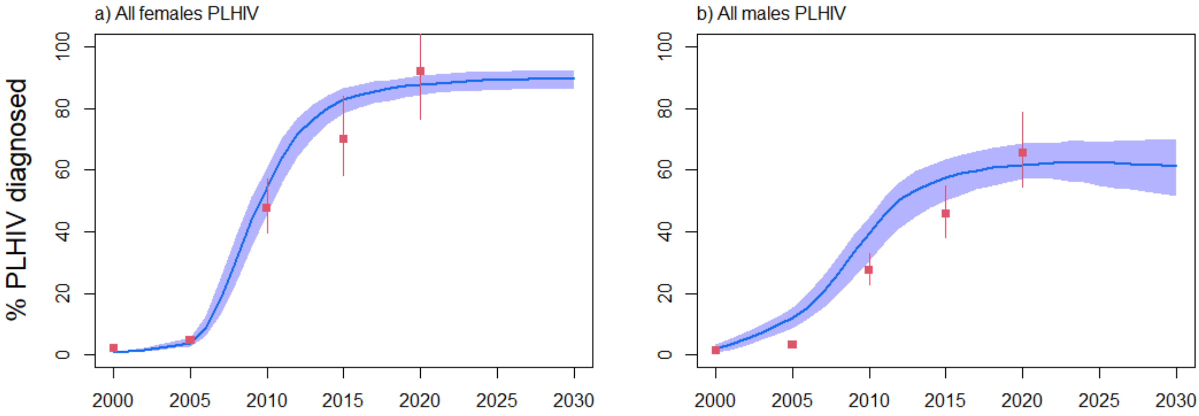

Figure S4j: Senegal model fitting to the fraction of a) all females living with HIV and b) all males living with HIV being diagnosed. Blue curves and shades represent median and 90% UI (5th and 95th percentiles across model fits), red squares and intervals represent estimates from UNAIDS Shiny90 used for model fitting.

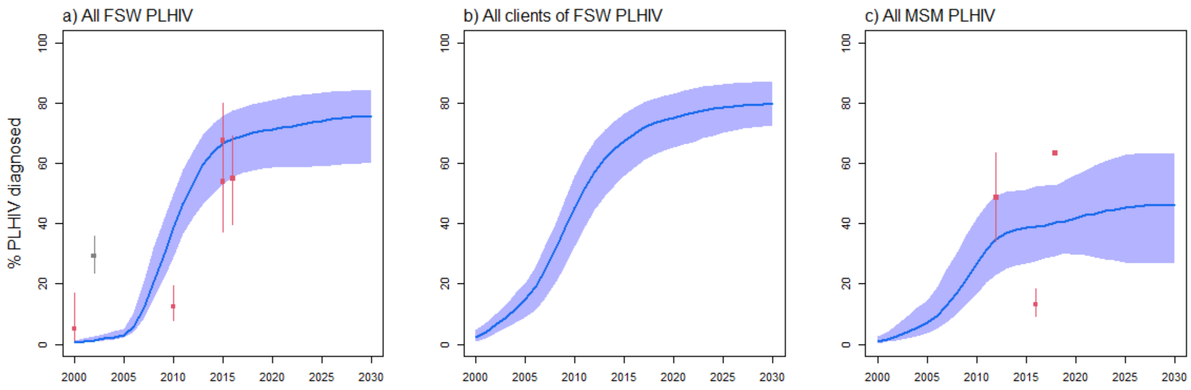

Figure S4k: Senegal model fitting to the fraction of a) all FSW living with HIV, b) all male clients of FSW living, and c) all MSM living with HIV being diagnosed. Blue curves and shades represent median and 90% UI (5th and 95th percentiles across model fits), red squares and intervals represent estimates from empirical surveys, whereas grey square in panel a) represent estimates from an STI clinic. Empirical estimates are self-reported and likely to be under-estimates.[147]

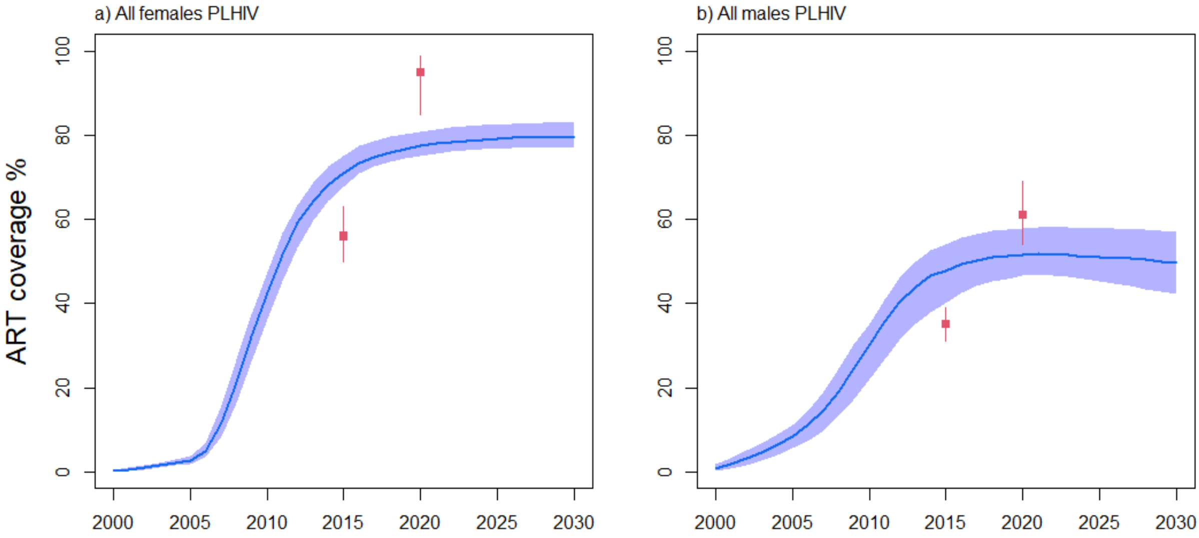

Figure S4l: Senegal model fitting to ART coverage among a) all females and b) all males. Blue curves and shades represent median and 90% UI (5th and 95th percentiles across model fits), whereas red squares and intervals represent estimates from UNAIDS (with 95%CI), using the Spectrum/EPP model. In panel a) our model predicts lower coverage of ART in 2020 compared to the Spectrum/EPP estimates as the latter are higher than proportions of female PLHIV which are diagnosed.

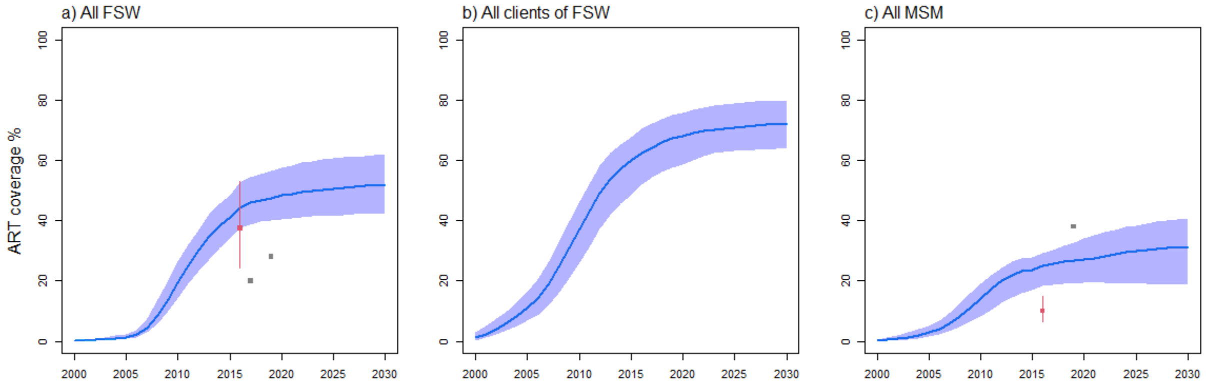

Figure S4m: Senegal model fitting to ART coverage among a) all FSW, b) all male clients of FSW, and c) all MSM. Blue curves and shades represent median and 90% UI (5th and 95th percentiles across model fits), whereas red squares and intervals represent empirical estimates from local surveys. Dark point and interval in panel c) represent self-reported use of ART (as opposed to estimates using viral load data), and all study participants reporting being on ART were virally suppressed (which was fitted by age in our model). Grey squares in panels a) and c) represent estimates from the UNAIDS key population atlas for which no report or underlying study could be identified. In panel c) our model predicted higher coverage of ART among MSM living with HIV than in Lyons et al. [78] as it was self-reported by study participants and does not agree well with estimates from all males.

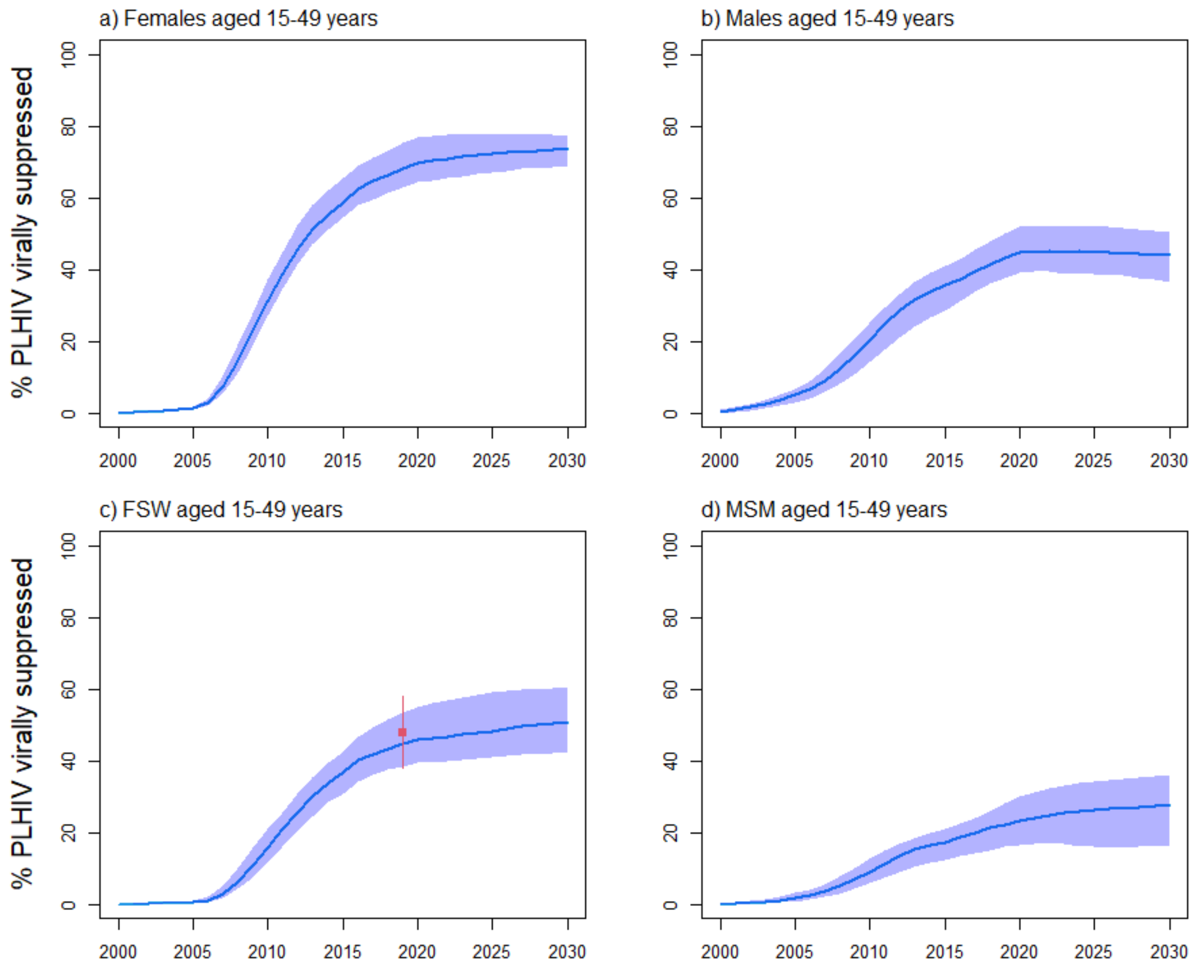

Figure S4n: Senegal model fitting to HIV viral load suppression coverage among a) all females, b) all males, c) all FSW, and d) all MSM aged 15-49 years living with HIV. Blue curves and shades represent median and 90% UI (5th and 95th percentiles across model fits), whereas red squares and intervals in panel c) represent empirical estimate from a local survey.

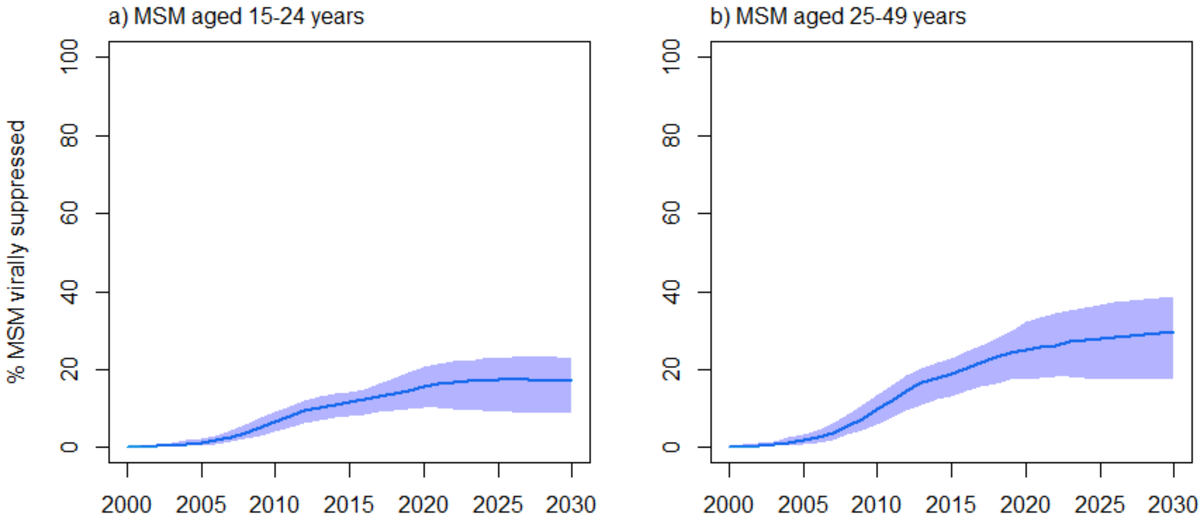

Figure S4o: Senegal model fitting to HIV viral load suppression coverage among all MSM aged a) 15-24 years and b) 25-49 years. Blue curves and shades represent median and 90% UI (5th and 95th percentiles across model fits. No data was available.

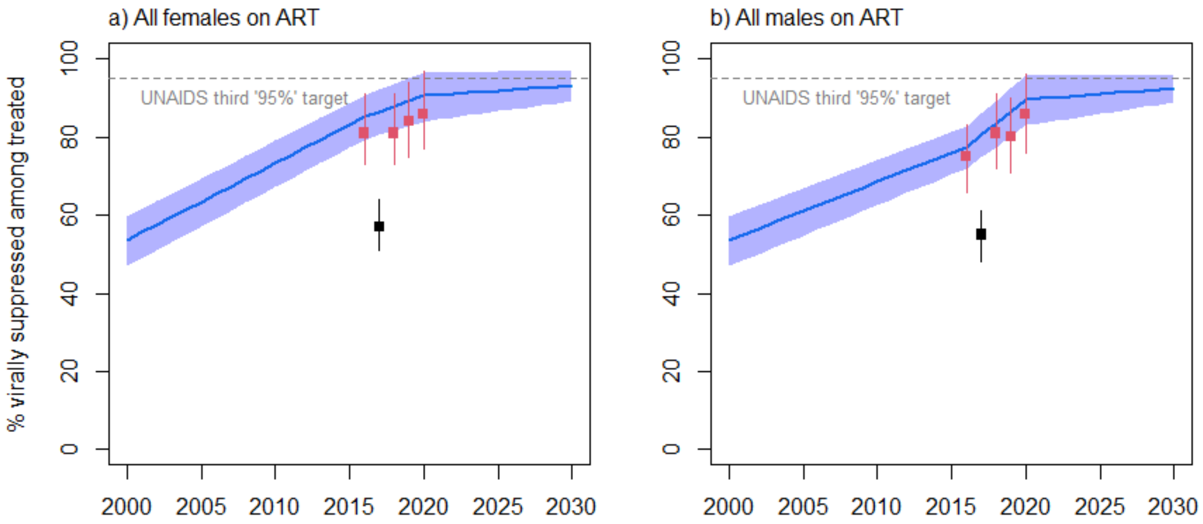

Figure S4p: Senegal model fitting to fractions of a) females and b) males living with HIV on ART which are virally suppressed (the third UNAIDSs "95%" indicator) over time. Blue curves and shades represent median and 95% UI (5th and 95th percentiles across model fits), whereas red squares and intervals represent estimates from UNAIDS used as parameters. The grey dashed line corresponds to the UNAIDS's third "95%" target whereby 95% of PLHIV on ART should be virally suppressed in 2025. The estimate for 2017 was assumed to be an outlier and not considered in our analysis.

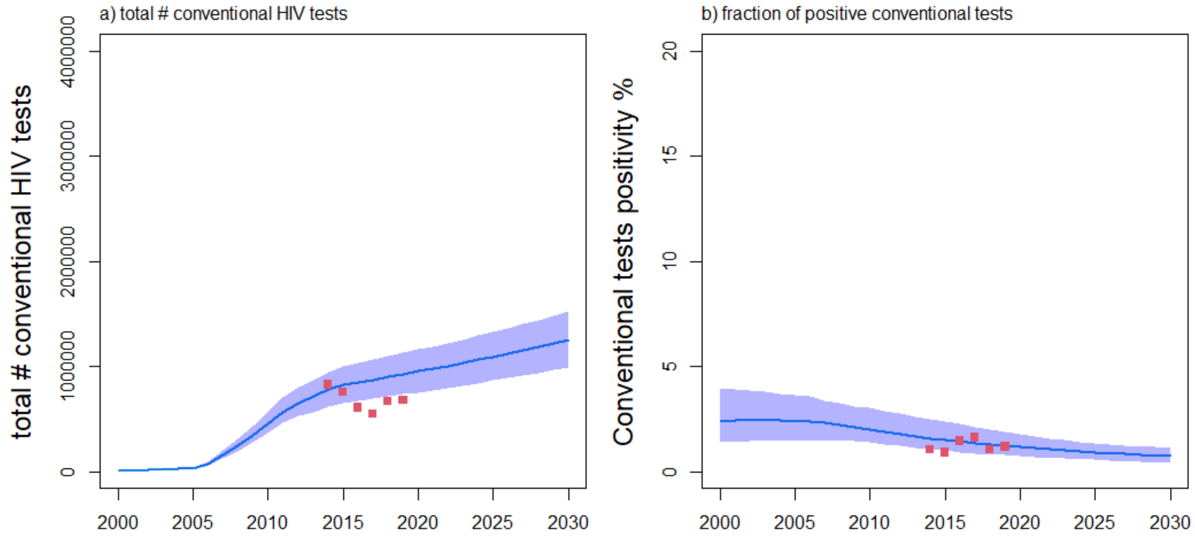

Figure S4q: Senegal model fitting to programmatic data on a) the total number of conventional tests among and b) proportion of these tests which were positive. Blue curves and shades represent median and 90% UI (5th and 95th percentiles across model fits), whereas red squares represent programmatic data communicated by UNAIDS.

References

1. Maheu-Giroux M, Vesga JF, Diabate S, Alary M, Baral S, Diouf D, et al. **Changing Dynamics of HIV Transmission in Cote d'Ivoire: Modeling Who Acquired and Transmitted Infections and Estimating the Impact of Past HIV Interventions (1976-2015)**. *J Acquir Immune Defic Syndr* 2017; 75(5):517-527.
2. Silhol R, Maheu-Giroux M, Soni N, Fotso AS, Rouveau N, Vautier A, et al. **Assessing the potential population-level impacts of HIV self-testing distribution among key populations in Cote d'Ivoire, Mali, and Senegal: a mathematical modelling analysis**. *medRxiv* 2023:2023.2008.2023.23294498.
3. Maheu-Giroux M, Diabate S, Boily MC, Jean-Paul N, Vesga JF, Baral S, et al. **Cost-Effectiveness of Accelerated HIV Response Scenarios in Cote d'Ivoire**. *J Acquir Immune Defic Syndr* 2019; 80(5):503-512.
4. Maheu-Giroux M, Vesga JF, Diabaté S, Alary M, Baral S, Diouf D, et al. **Population-level impact of an accelerated HIV response plan to reach the UNAIDS 90-90-90 target in Côte d'Ivoire: Insights from mathematical modeling**. *PLOS Medicine* 2017; 14(6):e1002321.
5. UNPD. **2019 Revision of World Population Prospects**. 2022; https://population.un.org/wpp/. (Date Accessed: 04/07/2021).
6. Vuylsteke B, Vandenhoudt H, Langat L, Semde G, Menten J, Odongo F, et al. **Capture-recapture for estimating the size of the female sex worker population in three cities in Côte d'Ivoire and in Kisumu, western Kenya**. *Trop Med Int Health* 2010; 15(12):1537-1543.
7. UNAIDS. **Key Populations Atlas**. 2021; https://kpatlas.unaids.org/dashboard. (Date Accessed: 07/11/2021).
8. Xu C, Jing F, Lu Y, Ni Y, Tucker J, Wu D, et al. **Summarizing methods for estimating population size for key populations: a global scoping review for human immunodeficiency virus research**. *AIDS Research and Therapy* 2022; 19(1):9.
9. Reniers G, Slaymaker E, Nakiyingi-Miiro J, Nyamukapa C, Crampin AC, Herbst K, et al. **Mortality trends in the era of antiretroviral therapy: evidence from the Network for Analysing Longitudinal Population based HIV/AIDS data on Africa (ALPHA)**. *Aids* 2014; 28 Suppl 4(4):S533-542.
10. Zanou B. **Rapport d'analyse du recensement général de la population et de l'habitation - 1998. Thème 2: Migrations**. Abidjan, Côte d'Ivoire: Institut national de la statistique; 2001.
11. République de Côte d'Ivoire. **Enquête Démographique et de Santé et à Indicateurs Multiples de Côte d'Ivoire 2011-2012**. Calverton, MD: Institut National de la Statistique et ICF International; 2012.
12. République de Côte d'Ivoire. **Enquête sur les Indicateurs du Sida, Côte d'Ivoire 2005**. Calverton, MD: Institut National de la Statistique (INS), Ministère de la Lutte contre le Sida [Côte d'Ivoire] et ORC Macro.; 2006.
13. République de Côte d'Ivoire. **Enquête Démographique et de Santé, Côte d'Ivoire 1998-1999**. Calverton, MD: Institute National de la Statistique et ORC Macro; 2001.
14. N'Cho S, Kouassi L, Koffi A, Schoemaker J, Barrère M, Barrère B, et al. **Enquête Démographique et de Santé, Côte d'Ivoire 1994**. Calverton, MD: Institut National de la Statistique et Macro International Inc.; 1995.
15. République du Mali. **Etude Bio-comportementale IST, VIH et tuberculose chez les femmes travailleuses du sexe et les routiers au Mali 2017-2019**. CELLULE SECTORIELLE DE LUTTE CONTRE LE VIH /SIDA LA TUBERCULOSE ET LES HEPATITES VIRALES; 2019 2019.
16. République du Mali. **Enquête Démographique et de Santé (EDSM V) Mali 2012-2013**. 2014.
17. République du Mali. **Enquête Démographique et de Santé du Mali 2006 (EDSM-IV)**. 2007.
18. République du Mali. **Mali Enquête Démographique et de Santé (EDSM-III) 2001**. 2002.
19. Coulibaly S, Dicko F, Traoré SM, Sidibé O, Seroussi M. **Mali Enquête Démographique et de Santé 1995-1996**. 1996.

20. Ndiaye S, Diouf PD, Ayad M. **Sénégal Enquête Démographique et de Santé (EDS-II) 1992/93**. Dakar, Sénégal: Ministère de l'Economie, des Finances et du Plan/Sénégal and Macro International; 1994. Available from: http://dhsprogram.com/pubs/pdf/FR55/FR55.pdf.
21. Ndiaye S, Ayad M, Gaye A. **Sénégal Enquête Démographique et de Santé (EDS-III) 1997**. Dakar, Sénégal: Ministère de l'Économie, des Finances et du Plan/Sénégal and Macro International; 1997. Available from: http://dhsprogram.com/pubs/pdf/FR89/FR89.pdf.
22. République du Sénégal. **Enquête Démographique et de Santé Sénégal 2005**. 2005. Available from: https://dhsprogram.com/pubs/pdf/FR177/FR177.pdf (Date Accessed: 14 April 2017).
23. République du Sénégal. **Enquête Démographique et de Santé à Indicateurs Multiples Sénégal (EDS-MICS) 2010-2011**. Claverton, Maryland, USA: ANSD and ICF International; 2012. Available from: http://dhsprogram.com/pubs/pdf/FR258/FR258.pdf.
24. Agence Nationale de la Statistique et de la Démographie/ANSD, ICF. **Senegal: Enquête Démographique et de Santé Continue (EDS-Continue 2018**. Dakar, Sénégal: ANSD/ICF; 2020. Available from: https://www.dhsprogram.com/pubs/pdf/FR367/FR367.pdf.
25. République du Sénégal. **Senegal: Enquête Démographique et de Santé Continue (EDS-Continue) 2017**. Dakar, Sénégal: ANSD and ICF; 2018. Available from: http://dhsprogram.com/pubs/pdf/FR345/FR345.pdf.
26. République du Sénégal. **Enquête Démographique et de Santé Continue (EDS-Continue) Senegal 2016**. Dakar, Sénégal: ANSD and ICF; 2017. Available from: http://dhsprogram.com/pubs/pdf/FR331/FR331.pdf.
27. République du Sénégal. **Enquête Démographique et de Santé Continue (EDS-Continue) Senegal 2015**. Rockville, Maryland, USA: ANSD/Sénégal and ICF; 2016. Available from: http://dhsprogram.com/pubs/pdf/FR320/FR320.pdf.
28. République du Sénégal. **Sénégal Enquête Démographique et de Santé Continue (EDS-Continue) 2014**. Rockville, Maryland, USA: ANSD/Senegal and ICF International; 2015. Available from: http://dhsprogram.com/pubs/pdf/FR305/FR305.pdf.
29. Agence Nationale de la Statistique et de la Démographie - ANSD/Sénégal, ICF International. **Senegal Enquête Démographique et de Santé Continue (EDS-Continue) 2012-2013**. Calverton, Maryland, USA: ANSD/Sénégal and ICF International; 2013. Available from: http://dhsprogram.com/pubs/pdf/FR288/FR288.pdf.
30. Vandepitte J, Lyerla R, Dallabetta G, Crabbé F, Alary M, Buvé A. **Estimates of the number of female sex workers in different regions of the world**. *Sex Transm Infect* 2006; 82 Suppl 3:iii18-25.
31. PEPFAR. **Côte d'Ivoire Country Operational Plan (COP/ROP) 2019 Strategic Direction Summary**. PEPFAR; 2019 2019.
32. Ministère de la Santé et de l'Hygiène Publique (MSHP). **Côte d'Ivoire Population-Based HIV Impact Assessment (CIPHIA) 2017-2018: Final Report**. Abidjan: MSHP; 2021. Available from: https://phia.icap.columbia.edu/wp-content/uploads/2021/05/CIPHIA-Final-Report_En.pdf.
33. République du Mali. **Cartographie et Estimation de la taille des Professionnelles de Sexe (PS) et des Hommes ayant des rapports Sexuels avec d'autres Hommes (HSH) couplées à l'étude bio-comportementale chez les HSH en matière de VIH au Mali**. MINISTERE DE LA LUTTE CONTRE LE SIDA; 2020 2020.
34. Mukandavire C, Walker J, Schwartz S, Boily MC, Danon L, Lyons C, et al. **Estimating the contribution of key populations towards the spread of HIV in Dakar, Senegal**. *J Int AIDS Soc* 2018; 21 Suppl 5:e25126.
35. Hawes SE, Critchlow CW, Faye Niang MA, Diouf MB, Diop A, Touré P, et al. **Increased risk of high-grade cervical squamous intraepithelial lesions and invasive cervical cancer among African women with human immunodeficiency virus type 1 and 2 infections**. *J Infect Dis* 2003; 188(4):555-563.
36. République de Côte d'Ivoire. **Étude sur le VIH et les facteurs de risques associés chez les hommes ayant des rapports sexuels avec des hommes à Abidjan, Côte d'Ivoire**. Abidjan, Côte d'Ivoire: Ministère de la Santé et de la Lutte contre le VIH/SIDA. Programme de lutte contre le Sida en direction des populations hautement vulnérables.; 2012.

37. République de Côte d'Ivoire. **Etude biologique et comportementale des IST, du VIH et du sida chez les hommes ayant des rapports sexuels avec des hommes (HSH) des villes d'Abidjan, Agboville, Bouaké, Gagnoa, et Yamoussoukro**. MSLS; 2016 2016.
38. République de Côte d'Ivoire. **Estimation De La Taille Des Travailleuses Du Sexe, Hommes Ayant Des Rapports Sexuels Avec D'autres Hommes, Usagers De Drogues Et Transgenres En Côte D'Ivoire**. 2020.
39. Inghels M, Kouassi AK, Niangoran S, Bekelynck A, Carillon S, Sika L, et al. **Cascade of Provider-Initiated Human Immunodeficiency Virus Testing and Counselling at Specific Life Events (Pregnancy, Sexually Transmitted Infections, Marriage) in Côte d'Ivoire**. *Sexually Transmitted Diseases* 2020; 47(1):54–61.
40. République du Sénégal. **Rapport de situation sur la riposte nationale a l'épidemie de VIH/SIDA Sénégal 2013-2014**. 2015. Available from: https://www.cnls-senegal.org/wp-content/uploads/2018/06/Rapport-2013-2014-de-situation-sur-la-riposte-nationale-%C3%A0-l%C3%A9pid%C3%A9mie-du-Vih-1.pdf.
41. Hakim AJ, Aho J, Semde G, Diarrassouba M, Ehoussou K, Vuylsteke B, et al. **The Epidemiology of HIV and Prevention Needs of Men Who Have Sex with Men in Abidjan, Cote d'Ivoire**. *PLoS One* 2015; 10(4):e0125218.
42. Aho J, Hakim A, Vuylsteke B, Semde G, Gbais HG, Diarrassouba M, et al. **Exploring Risk Behaviors and Vulnerability for HIV among Men Who Have Sex with Men in Abidjan, Cote d'Ivoire: Poor Knowledge, Homophobia and Sexual Violence**. *PLoS ONE* 2014; 9(6).
43. Moran A, Scheim A, Lyons C, Liestman B, Drame F, Ketende S, et al. **Characterizing social cohesion and gender identity as risk determinants of HIV among cisgender men who have sex with men and transgender women in Côte d'Ivoire**. *Annals of Epidemiology* 2020; 42:25-32.
44. Diabaté S, Kra O, Biékoua YJ, Pelletier SJ, Osso DG, Diané B, et al. **Pre-exposure prophylaxis among men who have sex with men in Côte d'Ivoire: a quantitative study of acceptability**. *AIDS Care* 2020:1-9.
45. USAID. **Cartographie Programmatique et Estimation de la Taille des HSH à Abidjan utilisant la méthode PLACE avancée**. USAID; 2017 2017.
46. Eubanks A, Dembélé Keita B, Anoma C, Dah TTE, Mensah E, Maradan G, et al. **Reaching a Different Population of MSM in West Africa With the Integration of PrEP Into a Comprehensive Prevention Package (CohMSM-PrEP ANRS 12369-Expertise France)**. *Journal of Acquired Immune Deficiency Syndromes (1999)* 2020; 85(3):292-301.
47. République de Côte d'Ivoire. **Enquête bio-comportementale chez les hommes ayant des rapports sexuels ave d'autres hommes (HSH) dans les villes de Divo, Daloa, Abengourou, Bouafle et Korhogo**. 2020.
48. Hakim AJ, Coy K, Patnaik P, Telly N, Ballo T, Traore B, et al. **An urgent need for HIV testing among men who have sex with men and transgender women in Bamako, Mali: Low awareness of HIV infection and viral suppression among those living with HIV**. *PloS One* 2018; 13(11):e0207363.
49. Kounta CH, Sagaon-Teyssier L, Coulaud P-J, Mora M, Maradan G, Bourrelly M, et al. **Transactional sex among men who have sex with men participating in the CohMSM prospective cohort study in West Africa**. *PLoS ONE* 2019; 14(11).
50. Lahuerta M., Patnaik P., Ballo T., Telly N., Knox J., Traore B., et al. **HIV Prevalence and Related Risk Factors in Men Who Have Sex with Men in Bamako, Mali: Findings from a Bio-behavioral Survey Using Respondent-Driven Sampling**. *AIDS and behavior* 2018; 22(7):2079-2088.
51. Wade AS, Kane CT, Diallo PA, Diop AK, Gueye K, Mboup S, et al. **HIV infection and sexually transmitted infections among men who have sex with men in Senegal**. *AIDS* 2005; 19(18):2133-2140.
52. Niang CK, Tapsoba P, Weiss E, Diagne M, Niang Y, Moreau MA, et al. **'It's raining stones': stigma, violence and HIV vulnerability among men who have sex with men in Dakar, Senegal**. *Culture, Health & Sexuality* 2003; 5(6):499-512.

53. Larmarange J, Wade AS, Diop AK, Diop O, Gueye K, Marra A, et al. **Men who have sex with men (MSM) and factors associated with not using a condom at last sexual intercourse with a man and with a woman in Senegal**. *PLoS One* 2010; 5(10).
54. Ndiaye P, Fall A, Tal-Dia A, Faye A, Diongue M. **Knowledge, attitudes and practices related to STD and HIV/AIDS: men having sex with men in Senegal**. *Rev Epidemiol Sante Publique* 2011; 59(5):305-311.
55. République du Sénégal. **Rapport Enquete Combinee Chez Les Hommes Ayant Des Rapports Sexuels Avec Des Hommes (HSH) Au Senegal (2014)**. 2014.
56. République du Sénégal. **Enquete Combinee Chez Les Hommes Ayant Des Rapports Sexuels Avec Des Hommes (HSH) Au Senegal**. 2017.
57. Lyons CE, Olawore O, Turpin G, Coly K, Ketende S, Liestman B, et al. **Intersectional stigmas and HIV-related outcomes among a cohort of key populations enrolled in stigma mitigation interventions in Senegal**. *AIDS* 2020; 34.
58. UNICEF. **LA SITUATION DES FEMMES ET DES ENFANTS EN CÔTE D'IVOIRE**. MINISTÈRE DU PLAN ET DU DÉVELOPPEMENT; 2016 2016.
59. République du Mali. **Demographic and Health Survey Mali 2018**. 2019.
60. République du Sénégal. **Senegal Enquête Démographique et de Santé Continue (EDS-Continue) 2012-2013**. Calverton, Maryland, USA: ANSD/Sénégal and ICF International; 2013. Available from: http://dhsprogram.com/pubs/pdf/FR288/FR288.pdf.
61. Garnett GP, Anderson RM. **Balancing sexual partnerships in an age and activity stratified model of HIV transmission in heterosexual populations**. *IMA J Math Appl Med Biol* 1994; 11(3):161-192.
62. Anderson RM, May RM. **Infectious diseases of humans**. Oxford University Press, USA; 1992.
63. Mackelprang RD, Baeten JM, Donnell D, Celum C, Farquhar C, de Bruyn G, et al. **Quantifying ongoing HIV-1 exposure in HIV-1-serodiscordant couples to identify individuals with potential host resistance to HIV-1**. *J Infect Dis* 2012; 206(8):1299-1308.
64. Naicker N, Kharsany AB, Werner L, van Loggerenberg F, Mlisana K, Garrett N, et al. **Risk Factors for HIV Acquisition in High Risk Women in a Generalised Epidemic Setting**. *AIDS Behav* 2015; 19(7):1305-1316.
65. Bellan SE, Dushoff J, Galvani AP, Meyers LA. **Reassessment of HIV-1 Acute Phase Infectivity: Accounting for Heterogeneity and Study Design with Simulated Cohorts**. *PLOS Medicine* 2015; 12(3):e1001801.
66. Rodger AJ, Cambiano V, Bruun T, Vernazza P, Collins S, Degen O, et al. **Risk of HIV transmission through condomless sex in serodifferent gay couples with the HIV-positive partner taking suppressive antiretroviral therapy (PARTNER): final results of a multicentre, prospective, observational study**. *The Lancet* 2019; 393(10189):2428-2438.
67. UNAIDS. **AIDSInfo**. 2021; https://aidsinfo.unaids.org/. (Date Accessed: 09/11/2021).
68. Ghys PD, Diallo MO, Ettiègne-Traoré V, Kalé K, Tawil O, Caraël M, et al. **Increase in condom use and decline in HIV and sexually transmitted diseases among female sex workers in Abidjan, Côte d'Ivoire, 1991–1998**. *AIDS* 2002; 16(2):251–258.
69. Bamba A, Grover E, Ezouatchi R, Thiam-Niangoin M, Papworth E, Grosso A, et al. **Étude biologique et comportementale des IST/VIH/SIDA chez les professionnelles du sexe du district d'Abidjan et examen des interventions en direction des populations clefs en Côte d'Ivoire**. Ministère de la Santé et de la Lutte contre le SIDA, ENDA Santé, Johns Hopkins University; 2014.
70. Lo Y, Sidibe C, Soro B, Kariburyo J. **Prévention et prise en charge de l'infection du VHI/SIDA et des autres IST ciblant les professionnels du sexe et leurs partenaires en Côte d'Ivoire: rapport de la mission technique d'appui OMS**. Geneva, Switzerland: Organisation mondiale de la Santé; 2009.
71. MSLS. **Analyse des connaissances, attitudes et pratiques des professionnels(les) du sexe dans dix-huit villes de Côte d'Ivoire**. Abidjan, Côte d'Ivoire: Ministère de la Santé et de la Lutte contre le Sida; 2012.
72. Ministère de la Santé du Mali. **Enquête Integrée Sur La Prévalence Et Les Comportements En Matière D'IST (ISBS) Menée Au Mali D'avril a Juin 2009, Rapport Final**. Bamako, Mali; 2010.

73. ENSC. **Enquete Nationale de Surveillance Combinee des IST et du VIH/SIDA (ENSC 2019), Composante Comportementale, Groupe Cible: PS**. 2020.
74. ENSC. **ENQUÊTE NATIONALE DE SURVEILLANCE COMBINEE DES IST ET DU VIH/SIDA, ENSC 2015**. 2015.
75. ENSC. **ENQUÊTE NATIONALE DE SURVEILLANCE COMBINEE DES ISTET DU VIH/SIDA, ENSC 2010**. 2010.
76. ENSC. **ENQUÊTE NATIONALE DE SURVEILLANCE COMBINEE DES IST ET DU VIH/SIDA, ENSC 2006**. 2006.
77. Vuylsteke BL, Ghys PD, Traoré M, Konan Y, Mah-Bi G, Maurice C, et al. **HIV prevalence and risk behavior among clients of female sex workers in Abidjan, Côte d'Ivoire**. *AIDS (London, England)* 2003; 17(11):1691-1694.
78. Lyons CE, Ketende S, Diouf D, Drame FM, Liestman B, Coly K, et al. **Potential Impact of Integrated Stigma Mitigation Interventions in Improving HIV/AIDS Service Delivery and Uptake for Key Populations in Senegal**. *J Acquir Immune Defic Syndr* 2017; 74 Suppl 1:S52-S59.
79. Larmarange J, Desgrées du Loû A, Enel C, Wade A. **Homosexualité et bisexualité au Sénégal : une réalité multiforme**. *Population* 2009; 64(4):723-756.
80. Wandel S, Egger M, Rangsin R, Nelson KE, Costello C, Lewden C, et al. **Duration from seroconversion to eligibility for antiretroviral therapy and from ART eligibility to death in adult HIV-infected patients from low and middle-income countries: collaborative analysis of prospective studies**. *Sex Transm Infect* 2008; 84 Suppl 1(Suppl_1):i31-i36.
81. Hughes JP, Baeten JM, Lingappa JR, Magaret AS, Wald A, de Bruyn G, et al. **Determinants of per-coital-act HIV-1 infectivity among African HIV-1-serodiscordant couples**. *J Infect Dis* 2012; 205(3):358-365.
82. Boily MC, Baggaley RF, Wang L, Masse B, White RG, Hayes RJ, et al. **Heterosexual risk of HIV-1 infection per sexual act: systematic review and meta-analysis of observational studies**. *Lancet Infect Dis* 2009; 9(2):118-129.
83. Patel P, Borkowf CB, Brooks JT, Lasry A, Lansky A, Mermin J. **Estimating per-act HIV transmission risk: a systematic review**. *Aids* 2014; 28(10):1509-1519.
84. Boily MC, Dimitrov D, Abdool Karim SS, Masse B. **The future role of rectal and vaginal microbicides to prevent HIV infection in heterosexual populations: implications for product development and prevention**. *Sex Transm Infect* 2011; 87(7):646-653.
85. Weller S, Davis K. **Condom effectiveness in reducing heterosexual HIV transmission**. *Cochrane Database Syst Rev* 2001; (3):CD003255.
86. DIPE. **Rapport annuel VIH/Sida du secteur santé en Côte d'Ivoire 2007-2008**. Abidjan, Côte d'Ivoire: Direction de l'Information, de la Planification et de l'Évaluation. Ministère de la Santé et de l'Hygiène Publique; 2009.
87. DIPE. **Rapport annuel des indicateurs VIH du secteur santé en Côte d'Ivoire 2009**. Abidjan, Côte d'Ivoire: Direction de l'Information, de la Planification et de l'Évaluation. Ministère de la Santé et de l'Hygiène Publique.; 2010.
88. DIPE. **Rapport annuel des indicateurs VIH du secteur Santé en Côte d'Ivoire 2010**. Abidjan, Côte d'Ivoire: Direction de l'Information, de la Planification et de l'Évaluation. Ministère de la Santé et de l'Hygiène Publique.; 2011.
89. DIPE. **Rapport annuel des indicateurs VIH du secteur santé en Côte d'Ivoire 2011**. Abidjan, Côte d'Ivoire: Direction de l'Information, de la Planification, et de l'Évaluation. Ministère de la Santé et de la Lutte contre le Sida; 2012.
90. DIPE. **Rapport annuel des indicateurs VIH du secteur santé en Côte d'Ivoire 2012**. Abidjan, Côte d'Ivoire: Direction de l'Information, de la Planification et de l'Évaluation. Ministère de la Santé et de la Lutte contre le SIDA.; 2013.
91. DIPE. **Rapport annuel des indicateurs VIH du secteur santé en Côte d'Ivoire 2013**. Abidjan, Côte d'Ivoire: Direction de l'Information, de la Planification et de l'Évaluation; 2014.

92. DIPE. **Rapport annuel des indicateurs VIH du secteur santé en Côte d'Ivoire 2014 - Non consolidé**. Abidjan, Côte d'Ivoire: Direction de l'Information, de la Planification et de l'Évaluation. Ministère de la santé et de la lutte contre le sida.; 2015.
93. Ekouevi DK, Balestre E, Ba-Gomis FO, Eholie SP, Maiga M, Amani-Bosse C, et al. **Low retention of HIV-infected patients on antiretroviral therapy in 11 clinical centres in West Africa**. *Trop Med Int Health* 2010; 15 Suppl 1(Suppl 1):34-42.
94. Vuylsteke B, Semdé G, Auld AF, Sabatier J, Kouakou J, Ettiègne-Traoré V, et al. **Retention and Risk Factors for Loss to Follow-up of Female and Male Sex Workers on Antiretroviral Treatment in Ivory Coast: A Retrospective Cohort Analysis**. *Journal of acquired immune deficiency syndromes (1999)* 2015; 68(Suppl 2):S99-S106.
95. MEF. **Enquête Ivoirienne sur la fécondité 1980-81 - Rapport Principal. Volume I.** Abidjan, Côte d'Ivoire: Ministère de l'Économie et des Finances. Direction de la Statistique; 1984.
96. MEF. **Enquête Ivoirienne sur la fécondité 1980-81 - Rapport Principal. Volume II.** Abidjan, Côte d'Ivoire: Ministère de l'Économie et des Finances. Direction de la Statistique.; 1984.
97. Schwartz S, Papworth E, Thiam-Niangoin M, Abo K, Drame F, Diouf D, et al. **An Urgent Need for Integration of Family Planning Services Into HIV Care: The High Burden of Unplanned Pregnancy, Termination of Pregnancy, and Limited Contraception Use Among Female Sex Workers in Côte d'Ivoire**. *JAIDS Journal of Acquired Immune Deficiency Syndromes* 2015; 68:S91.
98. Diallo D, Sangare O, Traore M, Dolo A. **FRÉQUENCE DES MST/SIDA CHEZ LES PROSTITUÉES A BAMAKO**. *Médecine d'Afrique Noire* 1997; 44(6).
99. Mulanga-Kabeya C, Morel E, Patrel D, Delaporte E, Bougoudogo F, Maiga YI, et al. **Prevalence and risk assessment for sexually transmitted infections in pregnant women and female sex workers in Mali: is syndromic approach suitable for screening?** *Sexually Transmitted Infections* 1999; 75(5):358-359.
100. Trout CH, Dembélé O, Diakité D, Bougoudogo F, Doumbia B, Mathieu J, et al. **West African female sex workers in Mali: reduction in HIV prevalence and differences in risk profiles of sex workers of differing nationalities of origin**. *Journal of Acquired Immune Deficiency Syndromes (1999)* 2015; 68 Suppl 2:S221-231.
101. Keita A. **Etude Integree Sur La Prevalence Des IST/VIH et Des Comportements Sexuels De Cinq Populations Cibles Au Mali**: UNIVERSITE DU MALI; 2005.
102. Keita M., Coulibaly S., Coulibaly Y.I., Dicko I., Sogoba S., Traore F.B., et al. **First indirect measure of incidence and risk factors for recent infections with HIV-1 among female sex workers in the district of Bamako, Mali**. *American Journal of Tropical Medicine and Hygiene* 2013; 89(5 SUPPL. 1):233.
103. Tounkara FK, Téguété I, Guédou FA, Keita B, Alary M. **Prevalence and Factors Associated With HIV and Sexually Transmitted Infections Among Female Sex Workers in Bamako, Mali**. *Sexually Transmitted Diseases* 2020; 47(10):679-685.
104. Ndoye I, Mboup S, De Schryver A, Van Dyck E, Moran J, Samb ND, et al. **Diagnosis of sexually transmitted infections in female prostitutes in Dakar, Senegal**. *Sex Transm Infect* 1998; 74 Suppl 1:S112-117.
105. Hakim A, Patnaik P, Telly N, Ballo T, Traore B, Doumbia S, et al. **High Prevalence of Concurrent Male-Male Partnerships in the Context of Low Human Immunodeficiency Virus Testing Among Men Who Have Sex With Men in Bamako, Mali**. *Sexually transmitted diseases* 2017; 44(9):565-570.
106. Moreau A, Placide Tapsoba, Abdoulaye Ly, Cheikh Ibrahima Niang, Diop AK. **Implementing STI/HIV prevention and care interventions for men who have sex with men in Senegal**. *Horizons Research Summary* 2007.
107. Aho J, Koushik A, Diakité SL, Loua KM, Nguyen VK, Rashed S. **Biological validation of self-reported condom use among sex workers in Guinea**. *AIDS Behav* 2010; 14(6):1287-1293.
108. Woolf-King SE, Muyindike W, Hobbs MM, Kusasira A, Fatch R, Emenyonu N, et al. **Vaginal Prostate Specific Antigen (PSA) Is a Useful Biomarker of Semen Exposure Among HIV-Infected Ugandan Women**. *AIDS Behav* 2017; 21(7):2141-2146.

109. Lépine A, Treibich C, Ndour CT, Gueye K, Vickerman P. **HIV infection risk and condom use among sex workers in Senegal: evidence from the list experiment method**. *Health Policy Plan* 2020; 35(4):408-415.
110. The World Bank. **West Africa HIV/AIDS Epidemiology and Response Synthesis: Implications for prevention**. Washington, DC, USA; 2008. Available from: https://openknowledge.worldbank.org/server/api/core/bitstreams/5362e1a3-bba8-5bf4-9d0d-76ff0e825a15/content (Date Accessed: 04/11/2020).
111. Benoit SN, Gershy-Damet GM, Coulibaly A, Koffi K, Sangare VS, Koffi D, et al. **Seroprevalence of HIV infection in the general population of the Côte d'Ivoire, West Africa**. *Journal of Acquired Immune Deficiency Syndromes* 1990; 3(12):1193-1196.
112. Ministry of Women - Family - and Children. **Violence Against Children and Youth In Côte d'Ivoire. Findings from a National survey**. 2020. Available from: https://www.togetherforgirls.org/wp-content/uploads/2020/10/RAPPORT-VACS-CI-ANGLAIS-final-06-10-2020.pdf.
113. Koffi K, Gershy-Damet GM, Peeters M, Soro B, Rey JL, Delaporte E. **Rapid spread of HIV infections in Abidjan, Ivory Coast, 1987-1990**. *European Journal of Clinical Microbiology & Infectious Diseases: Official Publication of the European Society of Clinical Microbiology* 1992; 11(3):271-273.
114. Mastro TD. **Increase in condom use and decline in HIV and sexually transmitted diseases among female sex workers in Abidjan, Côte d'Ivoire, 1991-1998, by Ghys et al**. *AIDS* 2003; 17 Suppl 4:S121-122.
115. Bastien V. **Espace Confiance: des services orientés vers la santé sexuelle. Dispositifs innovants des associations de lutte contre le VIH/sida en Afrique de l'Ouest**. Paris, France: SIDACTION; 2013.
116. Vuylsteke B, Semdé G, Sika L, Crucitti T, Ettiègne Traoré V, Buvé A, et al. **HIV and STI Prevalence among Female Sex Workers in Côte d'Ivoire: Why Targeted Prevention Programs Should Be Continued and Strengthened**. *PLoS ONE* 2012; 7(3).
117. République de Côte d'Ivoire. **Enquête bio-comportementale et cartographique en direction des travailleuses de sexe dans cinq localités de la Côte d'Ivoire : Katiola, Yamoussoukro, Soubré, Aboisso, Agboville.**; 2020.
118. République de Côte d'Ivoire. **PLAN STRATEGIQUE NATIONAL DE LA SURVEILLANCE DU VIH ET DES IST 2020-2024**. DIRECTION DE L'INFORMATIQUE ET DE ET DE L'INFORMATION SANITAIRE; 2019 2019.
119. USAID. **LINKAGES CÔTE D'IVOIRE Summary of Achievements**. USAID; 2019 2019.
120. UNAIDS. **UNAIDS data 2018**. https://www.unaids.org/sites/default/files/media_asset/unaids-data-2018_en.pdf.
121. UNICEF. **Enquête à Indicateurs Mulitples MICS2000 - Côte d'Ivoire**. Abidjan, Côte d'Ivoire: UNICEF; 2000.
122. UNICEF. **UNICEF Multiple Indicator Cluster Surveys (MICS)**. 2021; https://microdata.worldbank.org/index.php/catalog/MICS. (Date Accessed: 15/03/2021).
123. INS, UNFPA. **Enquête sur les connaissances, les attitudes et les pratiques des jeunes de 10-24 ans en matière de santé de la reproduction et d'insertion socio-économique**. Abidjan, Côte d'Ivoire: Institut National de la Statistique, Fonds des Nations Unies pour la Population; 2009.
124. Sika G, N'Guessan K. **Comportements sexuels des PS face au risque du VIH/SIDA: une étude de la situation dans 8 villes de Côte d'Ivoire**. In: *Sixth African Population Conference*. Ouagadougou, Burkina Faso; 2011.
125. Thomann M, Grosso A, Wilson PA, Chiasson MA. **'The only safe way to find a partner': rethinking sex and risk online in Abidjan, Côte d'Ivoire**. *Critical Public Health* 2020; 30(1):53-67.
126. UNAIDS. **UNAIDS Shiny90**. 2021; https://shiny90.unaids.org/. 2021).
127. Peeters M, Koumare B, Mulanga C, Brengues C, Mounirou B, Bougoudogo F, et al. **Genetic Subtypes of HIV Type 1 and HIV Type 2 Strains in Commercial Sex Workers from Bamako, Mali**. *AIDS Research and Human Retroviruses* 1998; 14(1):51-58.

128. Sullivan GP, Camara N, Dembele B, Guédou F, Thera I, Tounkara FK, et al. **P703 Pregnancy intention and prevalence according to HIV status among female sex workers in mali**. *Sexually Transmitted Infections* 2019; 95(Suppl 1):A307-A307.
129. UNICEF. **Enquête par grappes à indicateurs multiples (MICS), Rapport Final**. 2010.
130. UNICEF. **Enquête par grappes à Indicateurs Multiples (MICS), Rapport de Résultats Clés**. 2016.
131. Kanki P, M'Boup S, Marlink R, Travers K, Hsieh CC, Gueye A, et al. **Prevalence and risk determinants of human immunodeficiency virus type 2 (HIV-2) and human immunodeficiency virus type 1 (HIV-1) in west African female prostitutes**. *Am J Epidemiol* 1992; 136(7):895-907.
132. UNAIDS. **Epidemiological fact sheets: Senegal**. 2004.
133. République du Sénégal. **Bulletin Epidemiologique n° 9 de Surveillance du VIH/SIDA**. 2002.
134. Gomes Do Espirito Santo ME, Etheredge GD. **How to reach clients of female sex workers: a survey "by surprise" in brothels in Dakar, Senegal**. *Bull World Health Organ* 2002; 80:709-713.
135. Drame FM, Crawford EE, Diouf D, Beyrer C, Baral SD. **A pilot cohort study to assess the feasibility of HIV prevention science research among men who have sex with men in Dakar, Senegal**. *J Int AIDS Soc* 2013; 16 Suppl 3:18753.
136. Gouvernement du Sénégal. **Rapport de l'enquete sur les objectifs de la fin de decennie sur l'enfance (MICS - II - 2000)**. 2000.
137. DHS-MICS. **2010-11 Demographic and Health Survey and Multiple Indicator Cluster Survey**. 2010. Available from: https://dhsprogram.com/pubs/pdf/SR192/SR192.pdf (Date Accessed: 17 April 2017).
138. Lyons CE, Coly K, Bowring AL, Liestman B, Diouf D, Wong VJ, et al. **Use and Acceptability of HIV Self-Testing Among First-Time Testers at Risk for HIV in Senegal**. *AIDS and Behavior* 2019; 23(2):130-141.
139. Poteat T, Ackerman B, Diouf D, Ceesay N, Mothopeng T, Odette K-Z, et al. **HIV prevalence and behavioral and psychosocial factors among transgender women and cisgender men who have sex with men in 8 African countries: A cross-sectional analysis**. *PLoS Medicine* 2017; 14(11).
140. Laurent C, Seck K, Coumba N, Kane T, Samb N, Wade A, et al. **Prevalence of HIV and other sexually transmitted infections, and risk behaviours in unregistered sex workers in Dakar, Senegal**. *AIDS* 2003; 17(12):1811-1816.
141. Wang C, Hawes SE, Gaye A, Sow PS, Ndoye I, Manhart LE, et al. **HIV prevalence, previous HIV testing, and condom use with clients and regular partners among Senegalese commercial sex workers**. *Sex Transm Infect* 2007; 83(7):534-540.
142. Twahirwa Rwema JO, Lyons CE, Ketende S, Bowring AL, Rao A, Comins C, et al. **Characterizing the Influence of Structural Determinants of HIV Risk on Consistent Condom Use Among Female Sex Workers in Senegal**. *J Acquir Immune Defic Syndr* 2019; 81(1):63-71.
143. Mishra S, Silhol R, Knight J, Phaswana-Mafuya R, Diouf D, Wang L, et al. **Estimating the epidemic consequences of HIV prevention gaps among key populations**. *J Int AIDS Soc* 2021; 24 Suppl 3:e25739.
144. Cote AM, Sobela F, Dzokoto A, Nzambi K, Asamoah-Adu C, Labbe AC, et al. **Transactional sex is the driving force in the dynamics of HIV in Accra, Ghana**. *AIDS* 2004; 18(6):917-925.
145. Stone J, Mukandavire C, Boily M-C, Fraser H, Mishra S, Schwartz S, et al. **Estimating the contribution of key populations towards HIV transmission in South Africa**. *Journal of the International AIDS Society* 2021; 24(1):e25650.
146. Stover J, Glaubius R, Teng Y, Kelly S, Brown T, Hallett TB, et al. **Modeling the epidemiological impact of the UNAIDS 2025 targets to end AIDS as a public health threat by 2030**. *PLoS Med* 2021; 18(10):e1003831.
147. Soni N, Giguère K, Boily MC, Fogel JM, Maheu-Giroux M, Dimitrov D, et al. **Under-Reporting of Known HIV-Positive Status Among People Living with HIV: A Systematic Review and Meta-analysis**. *AIDS Behav* 2021; 25(12):3858-3870.
