# Supplementary material for: The impact of past HIV interventions and diagnosis gaps on new HIV acquisitions, transmissions, and HIV-related deaths in Côte d’Ivoire, Mali, and Senegal
Source: AIDS. 2024 Jul 1;38(12):1783–93. doi: 10.1097/QAD.0000000000003974 (PMC11356671; doi:10.1097/QAD.0000000000003974)
Supplement: Supplementary file 2 [file aids-38-1783-s002.pdf]

The impact of past HIV interventions and diagnosis gaps on new HIV acquisitions, transmissions, and HIV-related deaths in Côte d'Ivoire, Mali, and Senegal. Supplementary results.

Contents

Supplementary Results *2*

Impact of condom use and ART uptake on HIV outcomes over time (AFs). 2

HIV infections acquired and transmitted 6

HIV diagnosis and treatment gaps, and transmission PAF of PLHIV with undiagnosed infection 14

References *18*

Supplementary Results

Impact of condom use and ART uptake on HIV outcomes over time (AFs).

The fractions of new HIV infections and deaths averted by condom use, ART uptake, and both ($AF_{t_0-t_1}$), among all and specific risk groups were estimates by comparing the cumulative number of new HIV acquisitions and deaths over periods $[t_0 - t_1]$ between the base-case scenario (with current coverages of intervention) and counterfactual scenarios where condoms and/or ART do not protect against acquisition/transmission and/or do not reduce disease progression among the relevant risk groups.

$$AF_{t_0-t_1} = \frac{CI_{t_0-t_1}(without\ intervention) - CI_{t_0-t_1}(with\ intervention)}{CI_{t_0-t_1}(without\ intervention)}$$

Because separate interventions might avert the same secondary transmission$AF_{t_0-t_1}$, estimates for both condom use and ART combined are lower than the sum of the $AF_{t_0-t_1}$ for condom use and ART separately.

Our results suggest that condom use has averted high fractions of new infections in all modelled countries, with $AF_{2012-2021}$ estimates up to 74% (63-82%) in Côte d'Ivoire, 75% (66.0-85%) in Mali, and 82% (74-87%) in Senegal (Table 2a, Figure S5a), while consistently averting around a quarter of HIV-related deaths over each decade, with $AF_{2012-2021}$ of 22% (14-33%) in Côte d'Ivoire, 19% (13-32%) in Mali, and 30% (19-44%) in Senegal (Table 2b, Figure S5b). The estimated increases in the fractions of new HIV infections and deaths averted by ART over time reflected country differences in its progressive scale-up, which was faster in Senegal ($AF_{2012-2021}$ of 48% (43-54)) for new HIV infections, and 48% (35-55) for HIV-related deaths, with ART coverage increasing from 51% to 67% over the period) and Côte d'Ivoire ($AF_{t_0-t_1}$ of 42% (39-50) for new HIV infections, and 40% (31-48) for HIV-related deaths, with ART coverage increasing from 39% to 65% over the period) than in Mali (with $AF_{t_0-t_1}$ of 27% (22-34) of new HIV infections and 29% (21-37) of HIV-related deaths averted, with ART coverage increasing from 27% to 45% over the period). Our model results suggest that condoms and ART may continue averting an increasing proportion of new infections and deaths over the next decade (Figures S5a,b).

We estimated that the impact of condom use on HIV infections and deaths in Mali and Senegal over 2012-2021 was almost entirely due to its use by FSW and clients with all their partners (with >90% of FSW reporting using a condom at last paid sex, and ~80% in our model which assumes over-

reporting), with condom use among non-KP averting negligible proportions of new HIV infections and deaths (reflecting the low (<5% at last sex) reported use of condoms in national household surveys). In Côte d'Ivoire, condom use among non-KP (~16% at last sex[1]) might have averted 14% of new infections and 2% of HIV deaths over the period 2012-2021 (Table 2). In contrast, most of the impact on ART on new HIV infections in Côte d'Ivoire was due to its scale-up among non-KP (40%, vs 11% for the scale-up among KP), whereas ART scale-up among KP averted similar (Senegal) or higher (Mali) fractions of new infections than scale-up among non-KP. The fractions of HIV-related deaths averted by ART uptake by non-KP was always greater than the fractions averted by scale-up among KP.

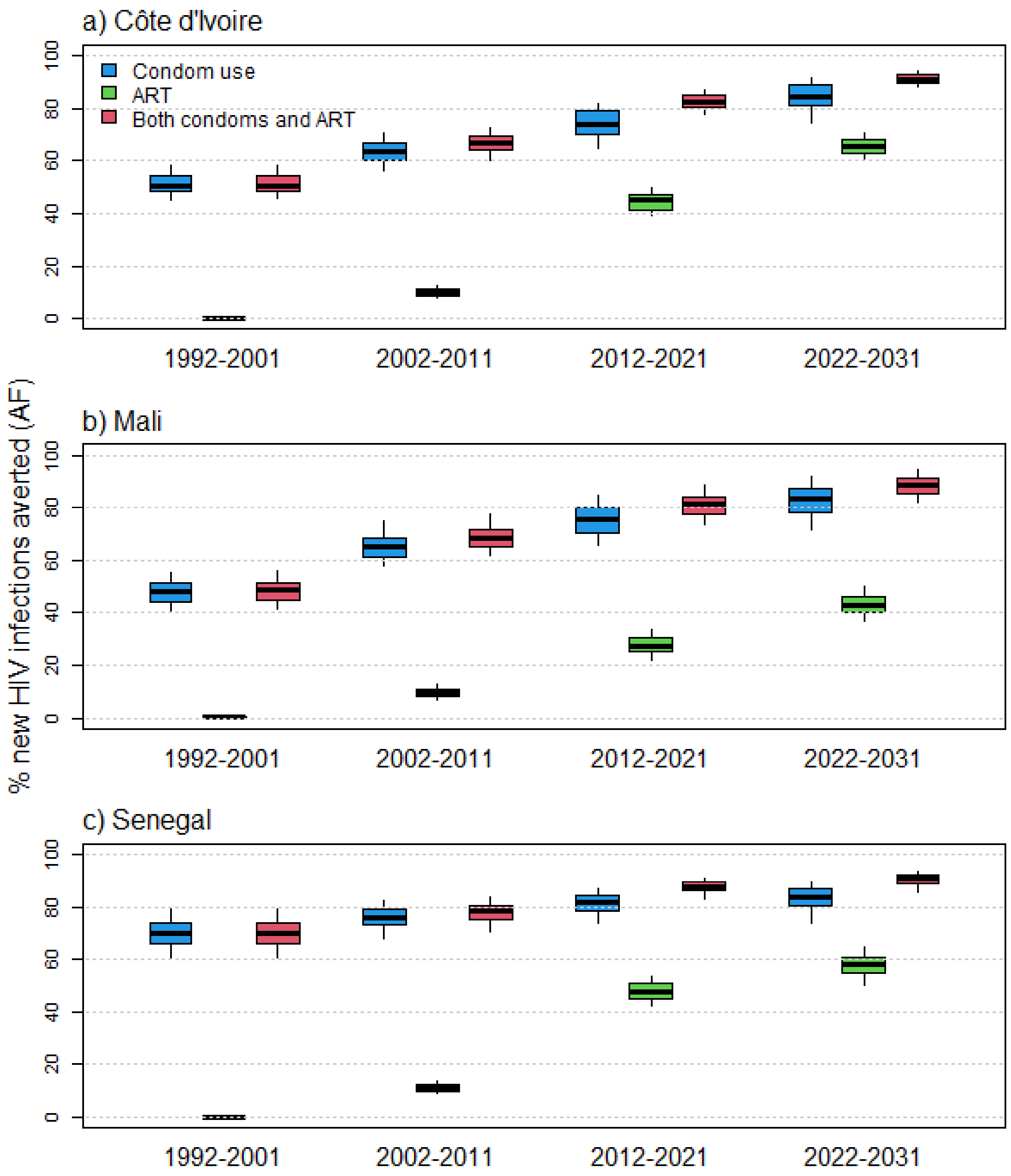

Figure S5a: Fraction of new HIV infections that may have been averted by the use of condoms (blue boxes and intervals), ART (green boxes and intervals), and both (red boxes and intervals) over successive decades (AFs). Boxes represent median and interquartile range, whereas error bars represent 90% uncertainty interval (UI) (5th and 95th percentiles) of AF estimates across all posterior parameter sets.

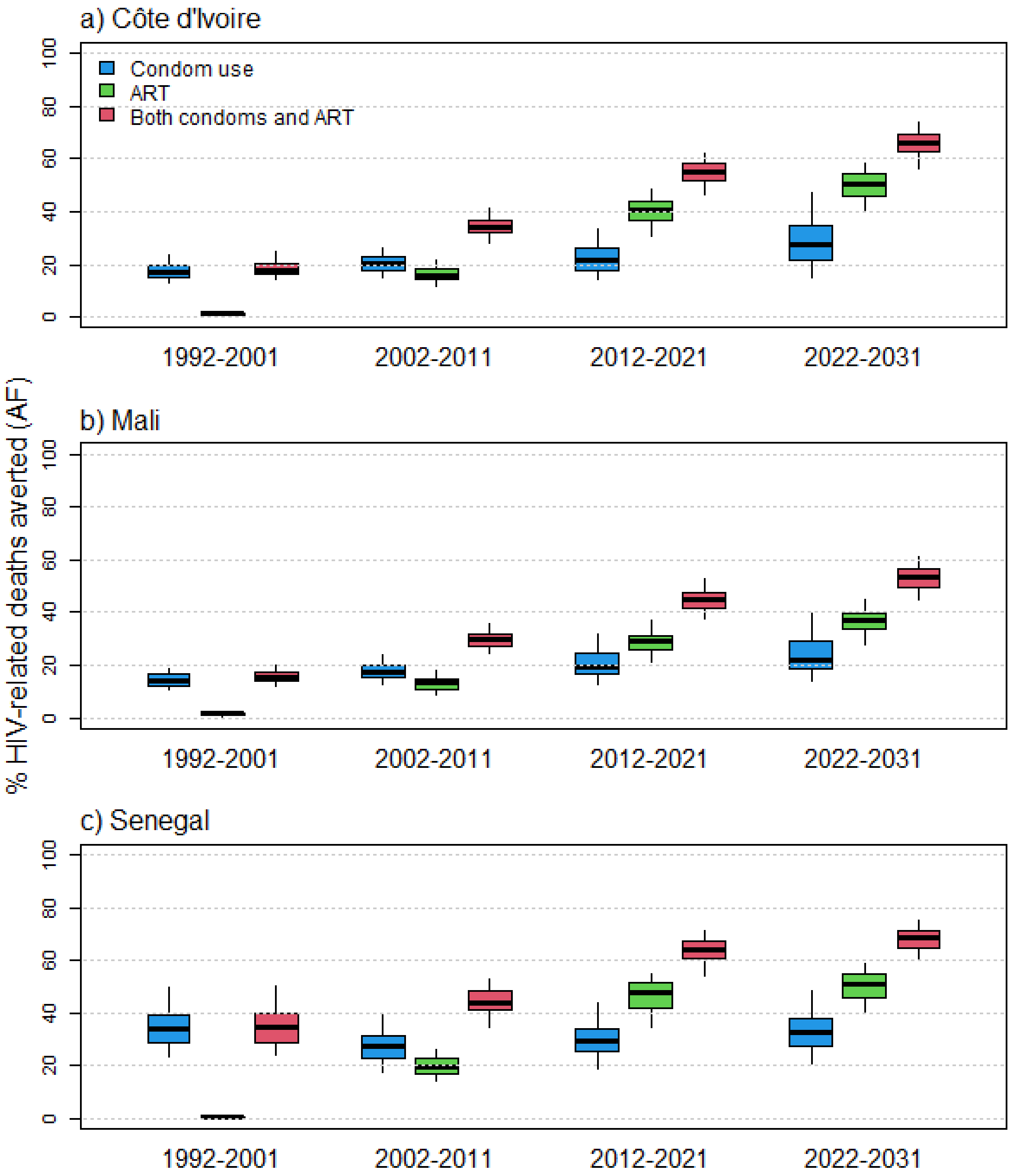

Figure S5b: Fraction of HIV-related deaths that may have been averted by the use of condoms (blue boxes and intervals), ART (green boxes and intervals), and both (red boxes and intervals) over successive decades (AFs). Boxes represent median and interquartile range, whereas error bars represent 90% uncertainty interval (UI) (5th and 95th percentiles) of AF estimates across all posterior parameter sets.

HIV infections acquired and transmitted

Table S3. Estimated fractions of all new HIV infections which were acquired, directly transmitted, or directly and indirectly transmitted by specific risk groups (tPAF) over 2012-2021. Median and 90%UI (5th and 95th percentiles) of indicators are shown.

	Fractions of infections acquired	Fractions of infections directly transmitted	Fractions of infections directly and indirectly transmitted (10-year tPAF)
**Côte d'Ivoire**			
All females	57% (52-63)	40% (35-47)	65% (61-70)
All males	43% (37-48)	60% (53-65)	78% (74-82)
FSW	3% (1-5)	3% (1-8)	7% (3-13)
Clients	8% (5-15)	13% (7-24)	20% (12-35)
MSM	2% (1-4)	3% (2-5)	3% (2-6)
Key populations[a]	14% (9-20)	20% (12-32)	27% (16-40)
Non-key populations females	54% (48-60)	37% (31-43)	59% (53-66)
Non-key populations males	32% (26-37)	43% (32-51)	57% (44-67)
**Mali**			
All females	58% (51-65)	38% (32-45)	61% (56-67)
All males	42% (35-49)	62% (55-68)	79% (75-83)
FSW	8% (5-14)	13% (6-24)	24% (13-39)
Clients	17% (10-28)[b]	35% (24-44)[b]	50% (35-63)[b]
MSM	3% (2-6)	5% (3-9)	6% (3-11)
Key populations[a]	29% (17-42)	54% (38-70)	63% (45-77)
Non-key populations females	50% (42-59)	25% (19-32)	39% (29-49)
Non-key populations males	21% (15-27)	21% (10-33)	25% (12-40)
**Senegal**			
All females	52% (43-60)	22% (16-29)	38% (28-48)
All males	48% (40-57)	78% (71-84)	89% (86-92)
FSW	7% (4-14)	7% (3-14)	17% (8-31)
Clients	9% (5-15)	27% (15-39)	36% (20-52)
MSM	25% (16-38)	37% (23-54)	39% (24-58)
Key populations[a]	43% (32-55)	72% (61-84)	79% (69-89)
Non-key populations females	44% (35-52)	15% (9-19)	22% (13-30)
Non-key populations males	13% (8-17)	13% (6-21)	15% (7-24)

[a] Includes FSW, their clients, and MSM

[b] Estimate which should be interpreted with caution due to the absence of specific survey among clients of FSW in Mali allowing to measure the size of this population, the prevalence of HIV or the levels of diagnosis/treatment (see discussion).

FSW: female sex workers; Clients: clients of FSW; MSM: men who have sex with men

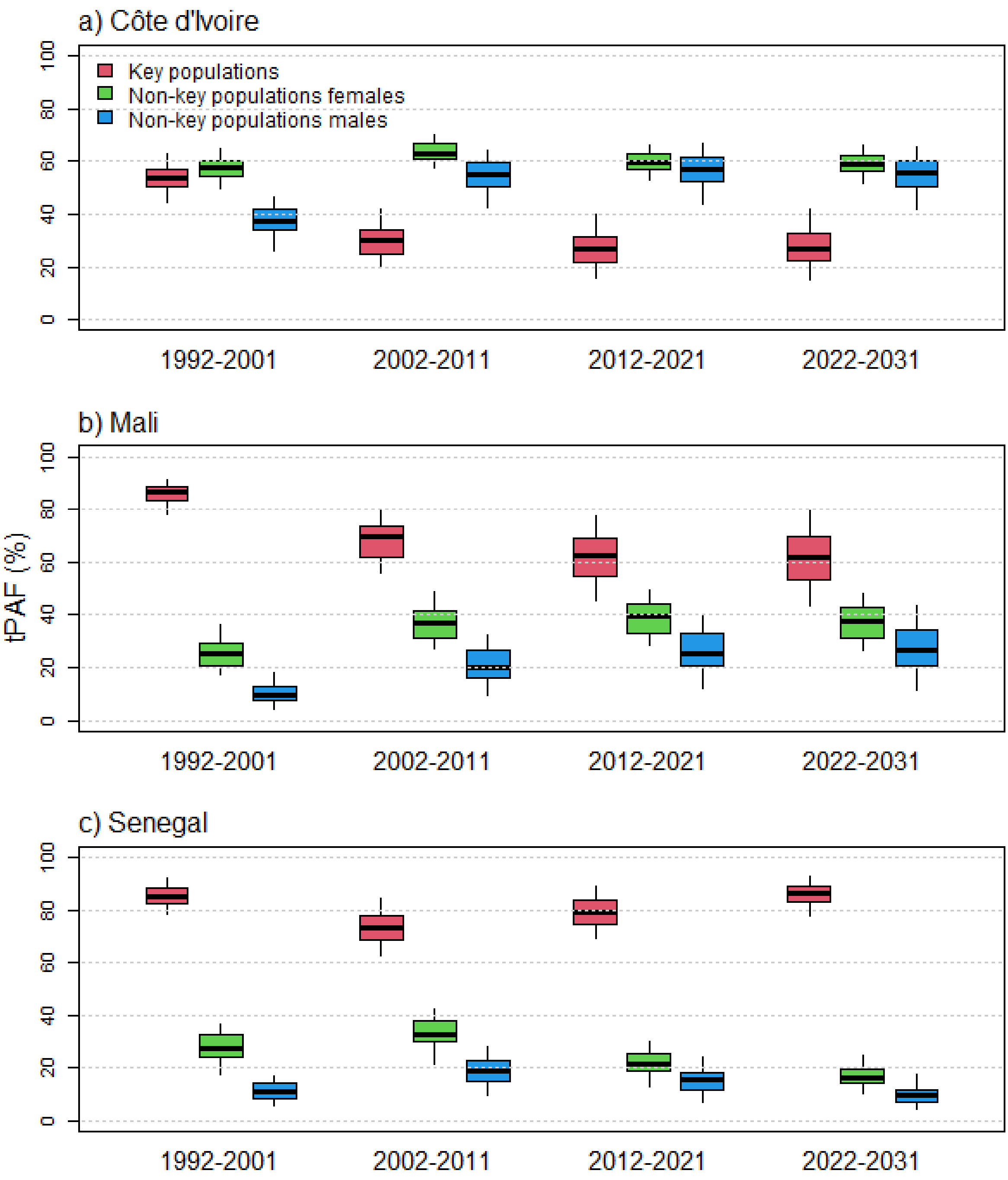

Figure S6: Changes in transmission PAF (tPAF) of key populations (female sex workers, their clients, and men who have sex with men, red boxes and intervals), non-key population females (green boxes and intervals), and non-key population males (blue boxes and intervals) over successive decades. Boxes represent median and interquartile range, whereas error bars represent 90% uncertainty interval (UI) (5th and 95th percentiles) of tPAF estimates across all posterior parameter sets.

Table S4a: Fraction of new HIV infections in Côte d'Ivoire over 2012-2021 acquired by all or specific groups (columns) which were directly and indirectly transmitted by specific groups (rows), commonly referred to as the transmission population attributable fraction (tPAFs). This can be interpreted as the fractions of new infections acquired in a population group (column) that would be averted by a 100% effective intervention blocking all transmission from another population (row)[a]. The sum of tPAF estimates from separate risk groups (columns) may exceed 100% as it accounts for onward secondary transmissions that may overlap for different groups[2]; as one can acquire HIV from more than one population group.

	All	All females	All males	Key Populations (including FSW clients)[b]	Non-Key Populations	Non-Key Populations females	Non-Key Populations males	Female sex workers	Clients of female sex workers	All men who have sex with men	Men who have sex with men and women	Men who have sex with men exclusively
All females and males	100%	100%	100%	100%	100%	100%	100%	100%	100%	100%	100%	100%
All females	65% (61-70)	43% (39-47)	95% (91-97)	74% (62-80)	64% (60-69)	43% (39-47)	100%	48% (43-53)	100%	7% (3-15)	11% (6-20)	2% (1-5)
All males	78% (74-82)	100%	49% (45-53)	68% (61-75)	80% (76-83)	100%	45% (41-50)	100%	48% (43-54)	97% (94-99)	95% (91-97)	100%
Key Populations (including FSW clients)[b]	27% (16-40)	28% (16-45)	24% (16-36)	68% (53-81)	18% (11-34)	22% (13-42)	11% (7-19)	100%	48% (31-68)	95% (89-98)	92% (85-95)	100%
Non-Key Populations	84% (73-90)	81% (69-90)	88% (80-93)	48% (31-65)	90% (82-95)	85% (73-92)	100%	28% (17-40)	66% (44-84)	7% (3-15)	11% (6-20)	2% (1-5)
Non-Key Populations females	59% (53-66)	39% (33-46)	88% (79-93)	46% (30-64)	61% (56-68)	40% (34-46)	100%	27% (16-40)	64% (41-83)	7% (3-15)	11% (6-20)	2% (1-5)
Non-Key Populations males	57% (44-67)	77% (61-87)	31% (22-40)	17% (11-26)	64% (52-72)	82% (65-90)	35% (27-43)	9% (5-15)	24% (15-35)	2% (1-6)	3% (2-8)	1% (0-1)
Female sex workers	7% (3-13)	5% (2-9)	10% (4-20)	30% (16-46)	3% (1-5)	3% (1-7)	2% (1-3)	21% (11-31)	42% (22-64)	0% (0-0)	0% (0-0)	0% (0-0)

Clients of female sex workers	20% (12-35)	25% (14-44)	13% (7-22)	36% (24-50)	17% (9-33)	20% (11-41)	10% (6-19)	100%	24% (14-38)	1% (0-2)	1% (1-3)	0% (0-0)
All men who have sex with men	3% (2-6)	2% (1-3)	5% (3-10)	15% (9-29)	2% (1-3)	2% (1-3)	1% (0-2)	0% (0-0)	1% (0-1)	94% (89-97)	91% (83-95)	100%
Men who have sex with men and women	2% (1-5)	2% (1-3)	3% (2-6)	9% (5-15)	2% (1-3)	2% (1-3)	1% (0-2)	0% (0-0)	1% (0-1)	52% (37-71)	50% (34-69)	56% (38-77)
Men who have sex with men exclusively	1% (1-3)	0% (0-1)	3% (1-7)	9% (4-21)	0% (0-1)	0% (0-1)	0% (0-0)	0% (0-0)	0% (0-0)	59% (35-75)	56% (34-72)	62% (39-77)

[a] As an example, 11% (6-20) of all new HIV infections among men who have sex with men and women over 2012-2021 could be averted by blocking all transmissions from all females, whereas 2% (1-3) of all new HIV infections among all females over 2012-2021 could be averted by blocking all transmissions from men who have sex with men and women.

[b] Key populations combine female sex workers, their clients, and men who have sex with men.

Table S4b: Fraction of new HIV infections in Mali over 2012-2021 acquired by all or specific groups (columns) which were directly and indirectly transmitted by specific groups (rows), commonly referred to as the transmission population attributable fraction (tPAFs). This can be interpreted as the fractions of new infections acquired in a population group (column) that would be averted by a 100% effective intervention blocking all transmission from another population (row)[a]. The sum of tPAF estimates from separate risk groups (columns) may exceed 100% as it accounts for onward secondary transmissions that may overlap for different groups[2]; as one can acquire HIV from more than one population group.

	All	All females	All males	Key Populations (including FSW clients)[b]	Non-Key Populations	Non-Key Populations females	Non-Key Populations males	Female sex workers	Clients of female sex workers	All men who have sex with men	Men who have sex with men and women	Men who have sex with men exclusively
All females and males	100%	100%	100%	100%	100%	100%	100%	100%	100%	100%	100%	100%
All females	61% (56-67)	39% (33-45)	92% (87-96)	71% (60-80)	57% (51-62)	39% (33-44)	100%	42% (35-48)	100%	2% (1-4)	3% (1-5)	1% (0-2)
All males	79% (75-83)	100%	49% (43-57)	72% (65-78)	82% (77-87)	100%	40% (32-48)	100%	50% (43-59)	99% (98-100)	99% (98-99)	100%
Key Populations (including FSW clients)[b]	63% (45-77)	69% (48-83)	56% (38-71)	91% (79-96)	50% (34-66)	62% (42-78)	25% (18-35)	100%	83% (69-93)	99% (98-100)	98% (97-99)	100%
Non-Key Populations	51% (35-66)	43% (25-59)	63% (45-80)	18% (7-37)	64% (49-77)	48% (31-66)	100%	8% (3-18)	26% (12-49)	2% (1-4)	3% (1-5)	1% (0-2)
Non-Key Populations females	39% (29-49)	22% (14-31)	62% (44-80)	17% (7-36)	47% (38-55)	24% (17-33)	100%	8% (3-18)	25% (11-49)	2% (1-4)	3% (1-5)	1% (0-2)
Non-Key Populations males	25% (12-40)	36% (21-56)	10% (4-17)	3% (1-7)	34% (21-50)	43% (25-63)	15% (8-25)	1% (0-3)	4% (2-9)	0% (0-1)	0% (0-1)	0% (0-0)
Female sex workers	24% (13-39)	18% (10-27)	33% (17-53)	55% (39-69)	12% (7-20)	15% (9-24)	6% (3-9)	33% (23-41)	78% (57-91)	0% (0-0)	0% (0-0)	0% (0-0)

Clients of female sex workers	50% (35-63)	64% (44-80)	31% (21-41)	57% (46-66)	46% (30-64)	56% (37-74)	23% (16-33)	100%	46% (36-57)	0% (0-1)	1% (0-1)	0% (0-0)
All men who have sex with men	6% (3-11)	4% (1-8)	9% (5-15)	12% (6-21)	3% (1-8)	4% (1-9)	1% (1-4)	0% (0-0)	0% (0-1)	99% (97-100)	98% (96-99)	100%
Men who have sex with men and women	5% (2-9)	4% (1-8)	6% (3-12)	8% (3-16)	3% (1-8)	4% (1-9)	1% (1-4)	0% (0-0)	0% (0-1)	70% (31-89)	70% (31-89)	71% (31-91)
Men who have sex with men exclusively	2% (1-5)	1% (0-1)	4% (1-10)	6% (2-13)	1% (0-1)	1% (0-1)	0% (0-0)	0% (0-0)	0% (0-0)	48% (19-81)	47% (19-80)	48% (20-82)

[a] As an example, 3% (1-5) of all new HIV infections among men who have sex with men and women over 2012-2021 could be averted by blocking all transmissions from all females, whereas 4% (1-8) of all new HIV infections among all females over 2012-2021 could be averted by blocking all transmissions from men who have sex with men and women.

[b] Key populations combine female sex workers, their clients, and men who have sex with men.

Table S4c: Fraction of new HIV infections in Senegal over 2012-2021 acquired by all or specific groups (columns) which were directly and indirectly transmitted by specific groups (rows), commonly referred to as the transmission population attributable fraction (tPAFs). This can be interpreted as the fractions of new infections acquired in a population group (column) that would be averted by a 100% effective intervention blocking all transmission from another population (row)[a]. The sum of tPAF estimates from separate risk groups (columns) may exceed 100% as it accounts for onward secondary transmissions that may overlap for different groups[2]; as one can acquire HIV from more than one population group.

	All	All females	All males	Key Populations (including FSW clients)[b]	Non-Key Populations	Non-Key Populations females	Non-Key Populations males	Female sex workers	Clients of female sex workers	All men who have sex with men	Men who have sex with men and women	Men who have sex with men exclusively
All females and males	100%	100%	100%	100%	100%	100%	100%	100%	100%	100%	100%	100%
All females	38% (28-48)	30% (24-38)	47% (32-63)	30% (16-46)	44% (36-52)	27% (21-36)	100%	43% (36-49)	100%	0% (0-1)	0% (0-1)	0% (0-0)
All males	89% (86-92)	100%	77% (69-85)	90% (85-94)	88% (85-91)	100%	47% (41-53)	100%	54% (44-62)	100%	100%	100%
Key Populations (including FSW clients)[b]	79% (69-89)	78% (65-89)	81% (69-88)	98% (95-99)	66% (53-78)	74% (60-86)	36% (29-43)	100%	89% (76-96)	100%	100%	100%
Non-Key Populations	30% (17-44)	29% (16-41)	30% (19-44)	5% (2-11)	48% (33-61)	33% (19-47)	100%	7% (2-16)	19% (8-41)	0% (0-1)	0% (0-1)	0% (0-0)
Non-Key Populations females	22% (13-30)	14% (7-20)	30% (19-44)	5% (2-11)	35% (25-42)	15% (9-22)	100%	7% (2-16)	19% (7-41)	0% (0-1)	0% (0-1)	0% (0-0)
Non-Key Populations males	15% (7-24)	26% (13-39)	4% (1-8)	1% (0-2)	26% (15-38)	29% (17-45)	12% (7-19)	1% (0-3)	3% (1-8)	0% (0-0)	0% (0-0)	0% (0-0)
Female sex workers	17% (8-31)	16% (8-29)	18% (8-34)	25% (12-42)	11% (5-21)	13% (6-24)	6% (3-11)	36% (27-46)	84% (65-94)	0% (0-0)	0% (0-0)	0% (0-0)

Clients of female sex workers	36% (20-52)	54% (33-73)	16% (7-29)	28% (14-51)	40% (24-60)	45% (27-66)	24% (14-34)	100%	48% (35-59)	0% (0-0)	0% (0-0)	0% (0-0)
All men who have sex with men	39% (24-58)	25% (10-40)	57% (40-73)	60% (38-79)	25% (10-40)	29% (12-45)	11% (5-21)	1% (0-2)	3% (1-8)	100%	100% (99-100)	100%
Men who have sex with men and women	31% (17-47)	25% (10-40)	39% (25-56)	40% (23-62)	25% (10-40)	29% (12-45)	11% (5-21)	1% (0-2)	3% (1-8)	69% (46-86)	70% (48-86)	69% (44-86)
Men who have sex with men exclusively	19% (11-32)	8% (4-14)	33% (19-49)	36% (21-55)	7% (4-13)	9% (5-15)	3% (2-6)	0% (0-1)	1% (0-2)	62% (38-82)	62% (39-83)	61% (37-80)

[a] As an example, 0% (0-1) of all new HIV infections among men who have sex with men and women over 2012-2021 could be averted by blocking all transmissions from all females, whereas 25% (10-40) of all new HIV infections among all females over 2012-2021 could be averted by blocking all transmissions from men who have sex with men and women.
[b] Key populations combine female sex workers, their clients, and men who have sex with men.

HIV diagnosis and treatment gaps, and transmission PAF of PLHIV with undiagnosed infection

Table S5. Estimated fraction of people living with HIV (PLHIV) with an undiagnosed infection in January 2022, fraction of PLHIV with a diagnosed but untreated infection in January 2022, fraction of PLHIV which are on ART, fraction of PLHIV with diagnosed infection which are on ART (UNAIDS 2nd "95%" target), distribution of PLHIV with an untreated infection in January 2022 in each country. Median and 90%UI (5th and 95th percentiles) of outcomes are shown.

	Fractions of PLHIV with an undiagnosed infection (2022)	Fractions of PLHIV with a diagnosed infection but not on ART (2022)	Fractions of PLHIV on ART (2022)	Fractions of PLHIV diagnosed that are on ART (2022)	Distribution (%) of PLHIV not on ART by risk group (2022)
**Côte d'Ivoire**					
All	25% (22-28)	7% (6-11)	67% (64-69)	90% (86-92)	100% (100-100)
All females	18% (15-21)	8% (6-12)	73% (71-75)	90% (86-92)	49% (44-56)
All males	37% (32-41)	6% (5-9)	56% (53-60)	90% (86-92)	51% (44-56)
FSW	31% (22-39)	4% (2-10)	64% (57-75)	95% (86-98)	3% (1-4)
Clients	37% (32-42)	6% (5-9)	56% (52-61)	90% (86-92)	10% (6-17)
MSM	44% (37-57)	3% (1-7)	52% (40-60)	95% (87-98)	3% (2-6)
Key populations (KP)	38% (33-42)	5% (4-8)	57% (53-61)	91% (87-94)	16% (12-23)
Non-KP females	18% (14-21)	8% (6-12)	73% (72-76)	90% (85-92)	47% (41-53)
Non-KP males	36% (32-41)	5% (3-8)	56% (53-62)	90% (86-92)	48% (39-56)
**Mali**					
All	48% (43-51)	6% (4-8)	46% (43-51)	89% (84-92)	100% (100-100)
All females	42% (38-44)	7% (5-9)	52% (49-55)	89% (84-92)	55% (48-63)
All males	58% (50-63)	4% (3-7)	38% (33-45)	89% (85-93)	45% (37-52)
FSW	45% (33-60)	4% (2-13)	49% (37-63)	93% (78-97)	4% (2-7)
Clients	58% (49-64)	5% (3-7)	38% (32-46)	89% (85-93)	19% (11-32)
MSM	40% (30-51)	3% (1-12)	56% (44-67)	94% (80-98)	4% (2-6)
Key populations (KP)	51% (46-58)	4% (3-8)	44% (38-51)	91% (84-94)	28% (16-41)
Non-KP females	42% (37-44)	7% (5-9)	52% (49-56)	89% (84-92)	51% (43-59)
Non-KP males	62% (54-68)	3% (2-6)	34% (28-42)	89% (84-92)	11% (7-16)

Senegal					
All	22% (18-27)	10% (7-14)	68% (63-71)	87% (83-91)	100% (100-100)
All females	11% (9-15)	10% (7-13)	79% (76-82)	89% (85-92)	39% (29-47)
All males	38% (31-43)	10% (7-16)	52% (47-58)	83% (76-89)	61% (53-71)
FSW	28% (18-41)	21% (11-35)	50% (41-59)	71% (55-84)	6% (3-10)
Clients	23% (16-33)	7% (5-10)	70% (61-77)	91% (87-93)	10% (5-17)
MSM	57% (41-71)	13% (7-27)	28% (20-36)	68% (50-81)	37% (24-56)
Key populations (KP)	42% (34-51)	12% (8-22)	45% (37-51)	78% (66-86)	54% (42-66)
Non-KP females	10% (7-13)	9% (7-13)	81% (77-84)	90% (86-93)	32% (24-40)
Non-KP males	24% (17-33)	6% (4-9)	68% (60-76)	90% (87-93)	27% (19-41)

[a] Estimate which should be interpreted with caution due to the absence of specific survey among clients of FSW in Mali allowing to measure the size of this population, the prevalence of HIV or the levels of diagnosis/treatment.

FSW: female sex workers; clients: clients of FSW; MSM: men who have sex with men; Key populations: FSW, clients, and MSM combined

Table S6. Estimated fraction of people living with HIV (PLHIV) with an undiagnosed infection in January 2012, distribution of PLHIV with an undiagnosed infection in January 2012, and fraction of all new HIV infections which were directly and indirectly transmitted (tPAF) by specific risk groups of PLHIV with an undiagnosed HIV infection over 2012-2021 in Cote d'Ivoire, Mali, and Senegal. Median and 90%UI (5th and 95th percentiles) of outcomes are shown.

	**Fractions of PLHIV with an undiagnosed infection (2012)**	**Distribution (%) of PLHIV with undiagnosed infections by risk group (2012)[a]**	**Transmission PAF of PLHIV with undiagnosed infection (2012-2021)**
**Côte d'Ivoire**			
All	53% (49-59)	100% (100-100)	74% (68-82)
All females	48% (42-53)	51% (46-58)	41% (36-47)
All males	61% (55-67)	49% (42-54)	59% (52-67)
FSW	59% (51-66)	3% (2-4)	5% (2-10)
Clients	58% (51-64)	9% (6-15)	15% (9-26)
MSM	71% (60-79)	4% (2-7)	3% (2-5)
Key populations (KP)	61% (55-67)	15% (12-22)	20% (12-31)
Non-KP females	47% (42-53)	49% (43-56)	37% (30-44)
Non-KP males	62% (55-68)	35% (30-41)	43% (31-53)
**Mali**			
All	68% (64-71)	100% (100-100)	82% (78-87)
All females	66% (63-70)	58% (50-65)	47% (42-54)
All males	70% (64-75)	42% (35-50)	67% (60-71)
FSW	55% (41-63)	4% (2-7)	18% (9-33)
Clients	69% (62-76)[b]	20% (12-31)	42% (28-54)
MSM	47% (40-60)	3% (2-4)	4% (2-8)
Key populations (KP)	63% (58-69)	28% (16-39)	51% (34-63)
Non-KP females	67% (64-71)	53% (46-61)	30% (21-39)
Non-KP males	76% (70-81)	19% (14-26)	22% (11-34)
**Senegal**			
All	37% (33-43)	100% (100-100)	72% (66-77)
All females	28% (23-36)	45% (37-53)	21% (15-29)
All males	50% (44-59)	55% (47-63)	65% (59-70)
FSW	47% (36-58)	7% (4-11)	10% (5-18)
Clients	43% (33-55)	16% (9-24)	23% (12-36)
MSM	65% (51-77)	18% (9-33)	32% (19-49)
Key populations (KP)	52% (46-59)	41% (30-54)	59% (49-68)
Non-KP females	26% (21-35)	39% (30-47)	11% (6-17)
Non-KP males	45% (36-58)	20% (13-28)	10% (5-19)

[a] Takes into account the size of the risk population as well as the proportion of PLHIV in the relevant subgroup with undiagnosed infection.

[b] Estimates for Mali should be interpreted with caution due to the absence of specific survey among clients of FSW in Mali allowing to measure the size of this population, the prevalence of HIV or the levels of diagnosis/treatment (see discussion).

FSW: female sex workers; clients: clients of FSW; MSM: men who have sex with men; Key populations: FSW, clients, and MSM combined

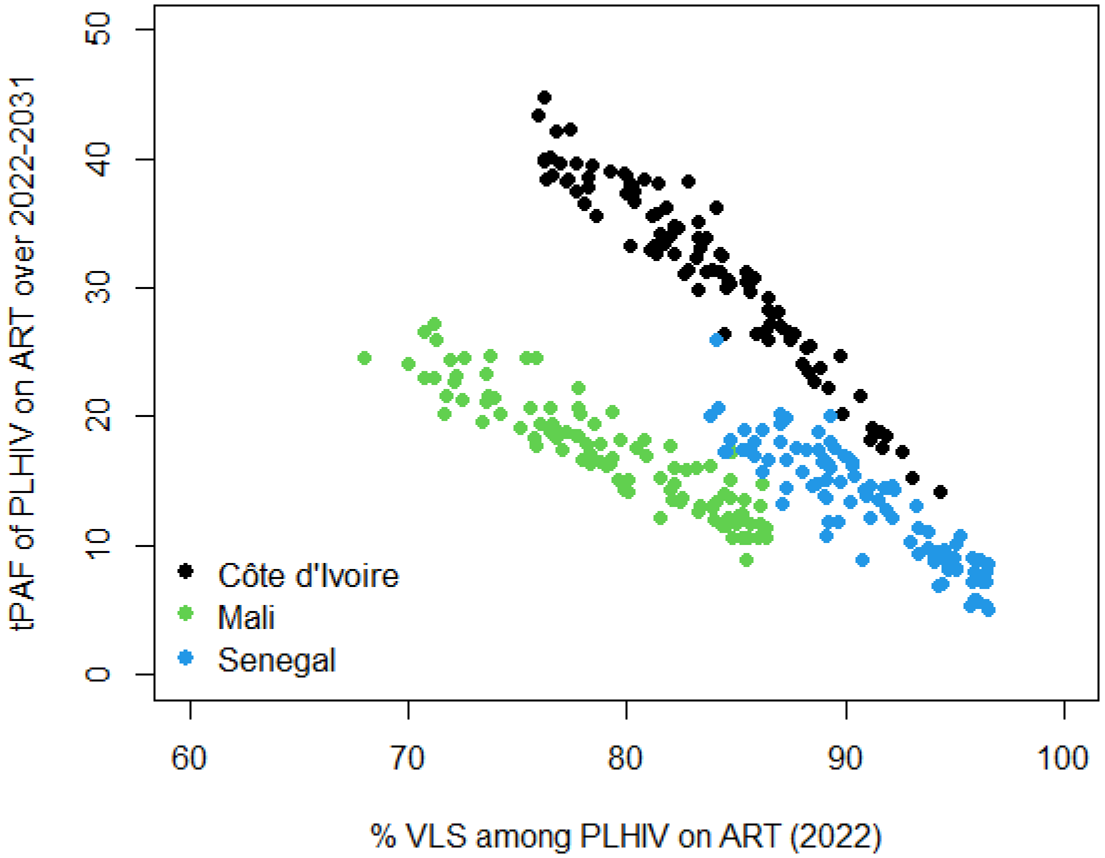

Figure S7: Fractions of new HIV infections (directly or indirectly) transmitted by PLHIV on ART in Côte d'Ivoire (black dots), Mali (green dots), and Senegal (blue dots) over 2022-2031, as a function of the average fraction of people living with HIV (PLHIV) on ART which have a suppressed viral load (VLS) in 2022. Each point represents the estimate from one single fitted simulation. The fraction of PLHIV with a suppressed viral load is a parameter informed by UNAIDS estimates assumed not to depend on the coverage of ART, and varies across simulations.

References

1. Ministère de la Santé et de l'Hygiène Publique (MSHP). **Côte d'Ivoire Population-Based HIV Impact Assessment (CIPHIA) 2017-2018: Final Report**. Abidjan: MSHP; 2021. Available from: https://phia.icap.columbia.edu/wp-content/uploads/2021/05/CIPHIA-Final-Report_En.pdf.

2. Mishra S, Silhol R, Knight J, Phaswana-Mafuya R, Diouf D, Wang L, et al. **Estimating the epidemic consequences of HIV prevention gaps among key populations**. *J Int AIDS Soc* 2021; 24 Suppl 3:e25739.
